# Importance of Anion−π Interactions in RNA GAAA and GGAG Tetraloops: A Combined MD and QM Study

**DOI:** 10.1021/acs.jctc.1c00756

**Published:** 2021-09-29

**Authors:** Reza Esmaeeli, María de las Nieves Piña, Antonio Frontera, Alberto Pérez, Antonio Bauzá

**Affiliations:** † Chemistry Department, University of Florida, Gainesville, Florida 32611, United States; ‡ Department of Chemistry, Universitat de les Illes Balears, Crta. de Valldemossa km 7.5, 07122 Palma, Baleares, Spain

## Abstract

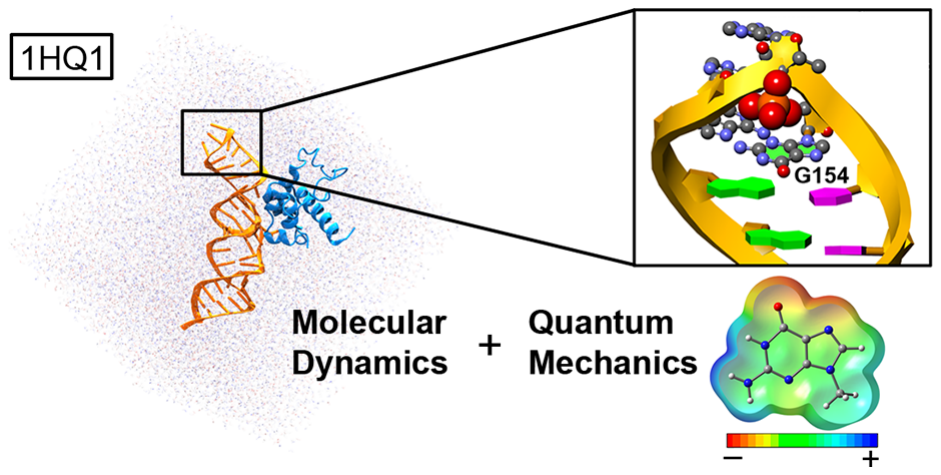

In this study, we demonstrate that anion−π interactions (an attractive noncovalent force between electron deficient π-systems and anions) are involved in the stabilization of GAAA and GGAG RNA tetraloops. Using the single recognition particle (SRP)–RNA complexes as a case of study, we combined molecular dynamics (MD) and quantum mechanics (QM) calculations to shed light on the structural influence of phosphate–G anion−π interactions and hydrogen bonds (HBs) involving K^+^/Mg^2+^ water clusters. In addition, the RNA assemblies herein were further characterized by means of the “atoms in molecules” (AIM) and noncovalent interactions plot (NCIplot) methodologies. We believe the results derived from this study might be important in the fields of chemical biology (RNA folding and engineering) and supramolecular chemistry (anion−π interactions) as well as to further expand the current knowledge regarding RNA structural motifs.

## Introduction

Unveiling and understanding the driving forces lying beneath RNA folding and recognition is of crucial importance to biology. The RNA functionality is intimately related to its ability to assemble into complex three-dimensional architectures, thus forming specific sites implicated in molecular recognition and catalysis phenomena.^1^ RNA structure is known to be of modular and hierarchical nature, where secondary structural elements (i.e., double stranded helices, single-stranded loops and hairpins) are linked by tertiary interactions driving the assembly process.^2−4^ More in particular, hairpins are often involved as RNA secondary structure motifs and exhibit diverse structures and biological and physical functionalities.^5^ For instance, they are involved in RNA tertiary contacts^6−8^ and play a pivotal role in transcription, regulation,^9,10^ mRNA degradation,^11−13^ and RNA interference.^14−16^ In this context, tetraloops are the most common RNA hairpins (∼50% of the hairpin structures in rRNA are tetraloops).^17,18^ While most tetraloop structures belong to the UNCG or GNRA motifs (N = any nucleotide and R = purine), d’Ascenzo and collaborators^19^ have recently proposed a more general identification scheme encompassing on one side the classical and well-studied U-turn^20^ and on the other a newly defined “Z-turn,” which is based on the UNCG tetraloop fold. In this regard, one of the key structural descriptors of the U-turn proposed by d’Ascenzo et al. was the formation of an oxygen−π or phosphate−π stacking contact between the first nucleobase of the loop and an OP atom from the third nucleotide.^21^ This intramolecular contact between the phosphate group and G has been well described and characterized in the field of supramolecular chemistry as an anion−π interaction (an attractive noncovalent force between an electron deficient π-system and an anion).^22^ While anion−π interactions have been contextualized in many chemistry-related fields of research^23−25^ (e.g., crystal engineering, materials science, catalysis), as well as in biology^26−28^ (protein–ligand interactions and enzyme chemistry), few studies have analyzed their implications in RNA folding motifs to date.^29−31^

Herein, our main goal was to analyze the influence of phosphate–G anion−π interactions on the structural stability of “U turn” RNA tetraloops, as well as the energetic and geometrical implications of protein–RNA binding on the interaction. To achieve this, we have used three single recognition particle (SRP)–RNA complexes as a case study (PDB codes 1HQ1, 1DUL, and 1JID). SRP–RNA complexes are present in all three kingdoms of life and are involved in the recognition and transport of specific proteins to cellular membranes for insertion or secretion,^32^ thus playing a crucial role in cell communication processes in both eukaryote and prokaryote organisms. Among the three systems studied, two of them (structures 1HQ1 and 1DUL) are part of the ribonucleoprotein core of *E. coli* and exhibit a GAAA tetraloop. The third one (structure 1JID) is present in *Homo sapiens* and shows a less common GGAG folding motif (see Computational Methods for structure selection details) linked to specific protein–RNA interactions.^33^ The GAAA motif is known to favor long-range RNA contacts.^34^ The experimental structures 1HQ1 and 1DUL containing this motif introduced a mutation to the wild-type GGAA to favor crystal lattice contacts leading to experimental determination.

**Figure 1:**
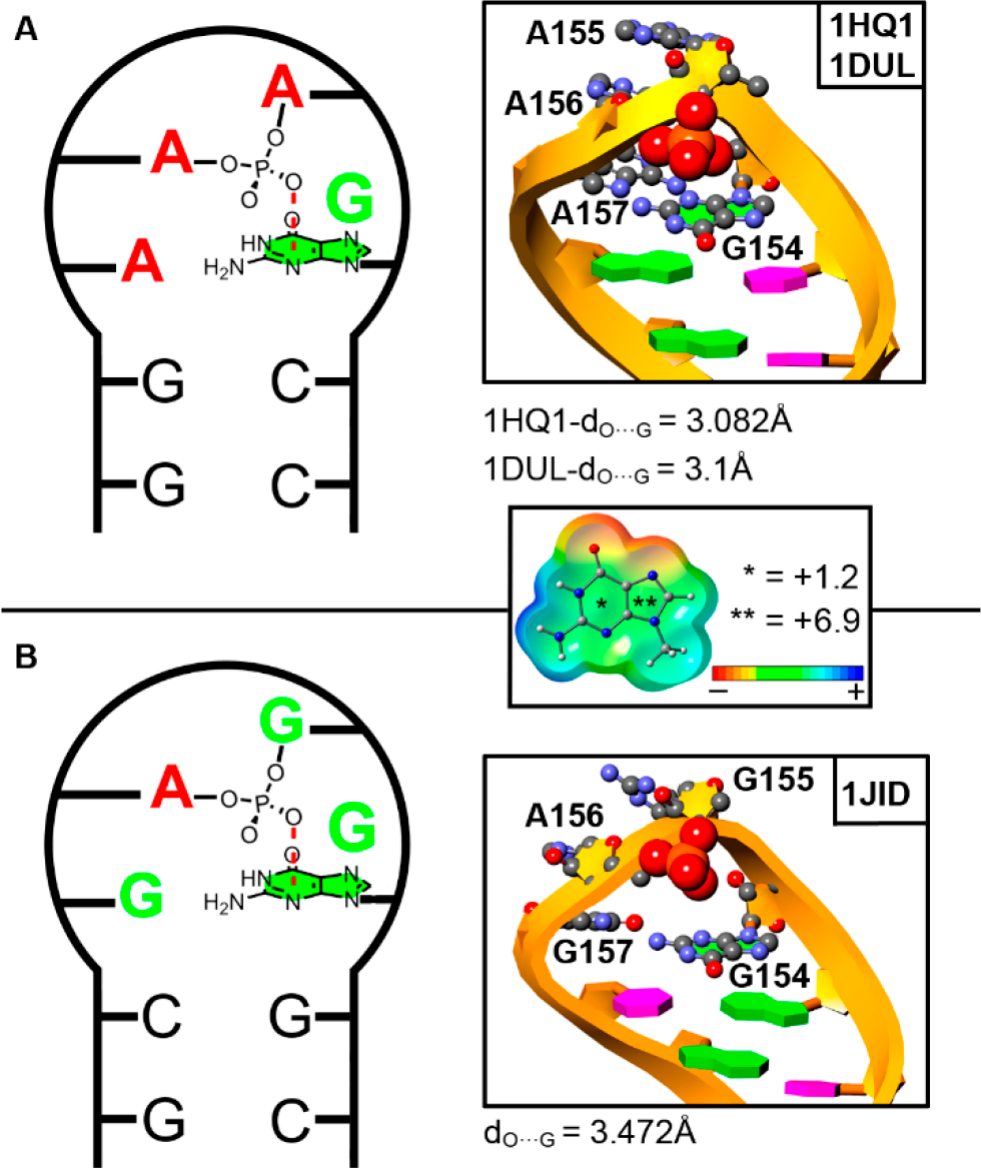
Schematic representation of the RNA tetraloop present in (A) 1HQ1 and 1DUL and (B) 1JID structures. The anion−π interaction is magnified in the right part of the figure. Middle: Electrostatic potential map of guanine. Energy values at concrete regions (* and **) of the surface are given in kcal/mol (0.002 au).

The ribonucleoprotein core, which we model, consists of the M domain of Ffh protein (*E. coli*) or SRP54 protein (*H. sapiens*) bound to the minor groove of domain IV of 4.5S RNA protein (*E. coli*) or 7S RNA (*H. sapiens*). This core region is the most conserved part of SRP across the three kingdoms of life. In fact, human SRP is functional even if its RNA is replaced by that of *E. coli*. SRP will identify a signal peptide sequence emerging from ribosomes early in the translation process. If identified, SRP will bind to that ribosome and stop the translation process while transporting the whole ribosome to specific receptors on the endoplasmic reticulum (eukaryotes) or cytoplasmic membrane (prokaryotes) where translation will resume. The M domain contains the recognition site for both the RNA and the peptide. As noticed in Figure 1, all three RNA loop assemblies exhibit an anion−π interaction between the phosphate group located between the second (A155/G155) and third (A156) nucleotides of the loop and the first base of the assembly (G154). The computed molecular electrostatic potential (MEP) surface of G revealed a positive electrostatic potential area covering both 5- and 6-membered rings (+6.9 and +1.2 kcal/mol, respectively), denoting a π-acidic character.

Our approach combines classical molecular dynamics (MD) simulations with quantum mechanics (QM) calculations at the RI-MP2/def2-TZVPD level of theory. More precisely, explicit solvent MD simulations of both the isolated and protein bound RNA structure were carried out to understand (i) the influence of the phosphate···G anion−π interaction on the tetraloop structure, (ii) the stabilizing role of K^+^ and Mg^2+^ water clusters, and (iii) the structural effects upon mutation of G. On the other hand, QM calculations shed light on the strength and directionality of anion−π and K^+^/Mg^2+^ water cluster hydrogen bonds (HBs). Finally, Bader’s theory of “atoms in molecules” (AIM) and NCIplot (noncovalent interactions plot) analyses further characterized the interactions studied herein from a charge-density perspective. As far as our knowledge extends, this report represents the first computational study of anion−π interactions in RNA tetraloop folding motifs. Hence, the results derived from this study might be useful for both chemical biologists (RNA folding and engineering) and supramolecular chemists, as well as to increase the visibility of the anion−π interaction among the RNA community.

## Results and Discussion

### Molecular Dynamics Simulations

**Figure 2:**
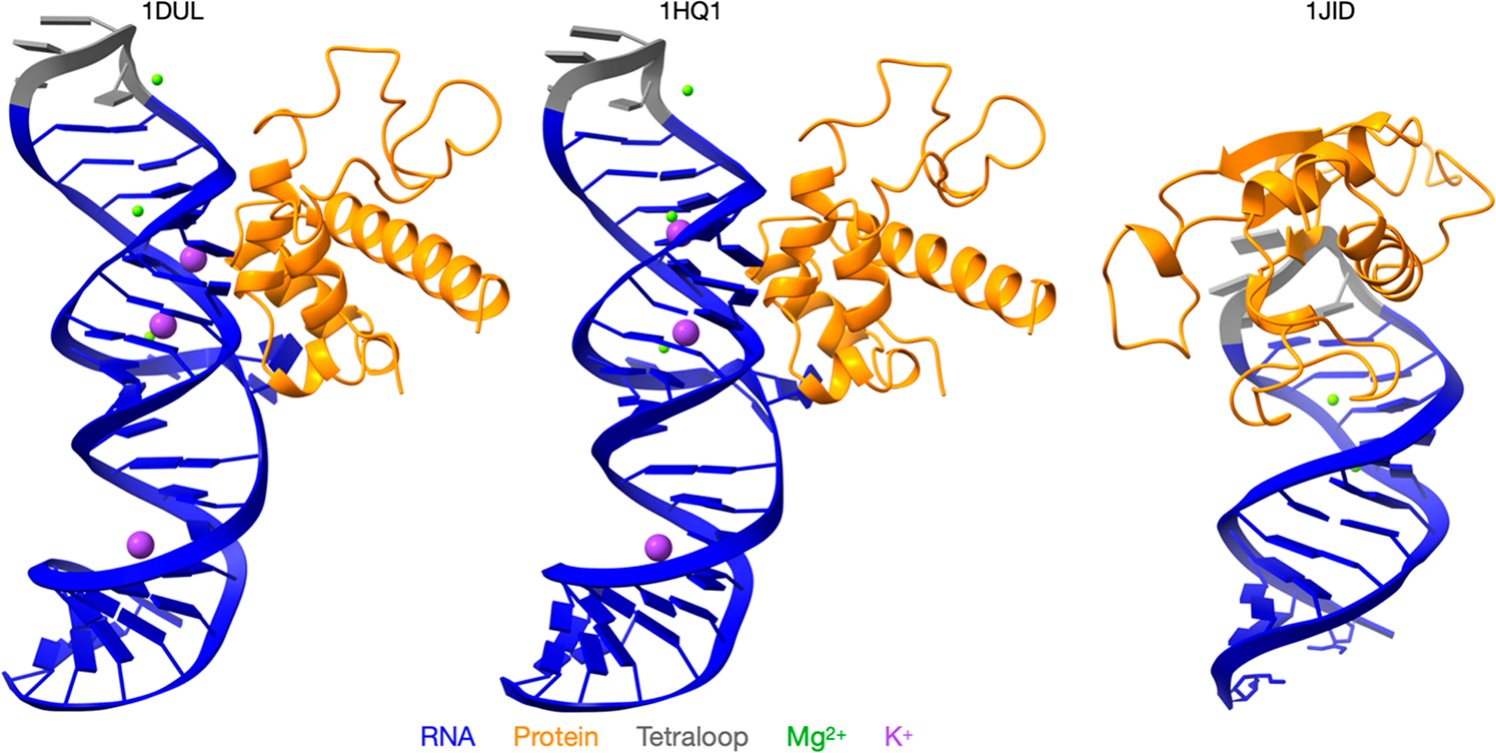
Three SRP systems of this study. 1DUL, left; IHQ1, middle; and 1JID, right. RNA is shown in blue and protein in orange and tetraloop is highlighted in gray. Mg^2+^ and K^+^ are denoted in green and magenta, respectively. Unlike the other two systems, in 1JID, tetraloop is in direct interaction with the protein.

**Figure 3:**
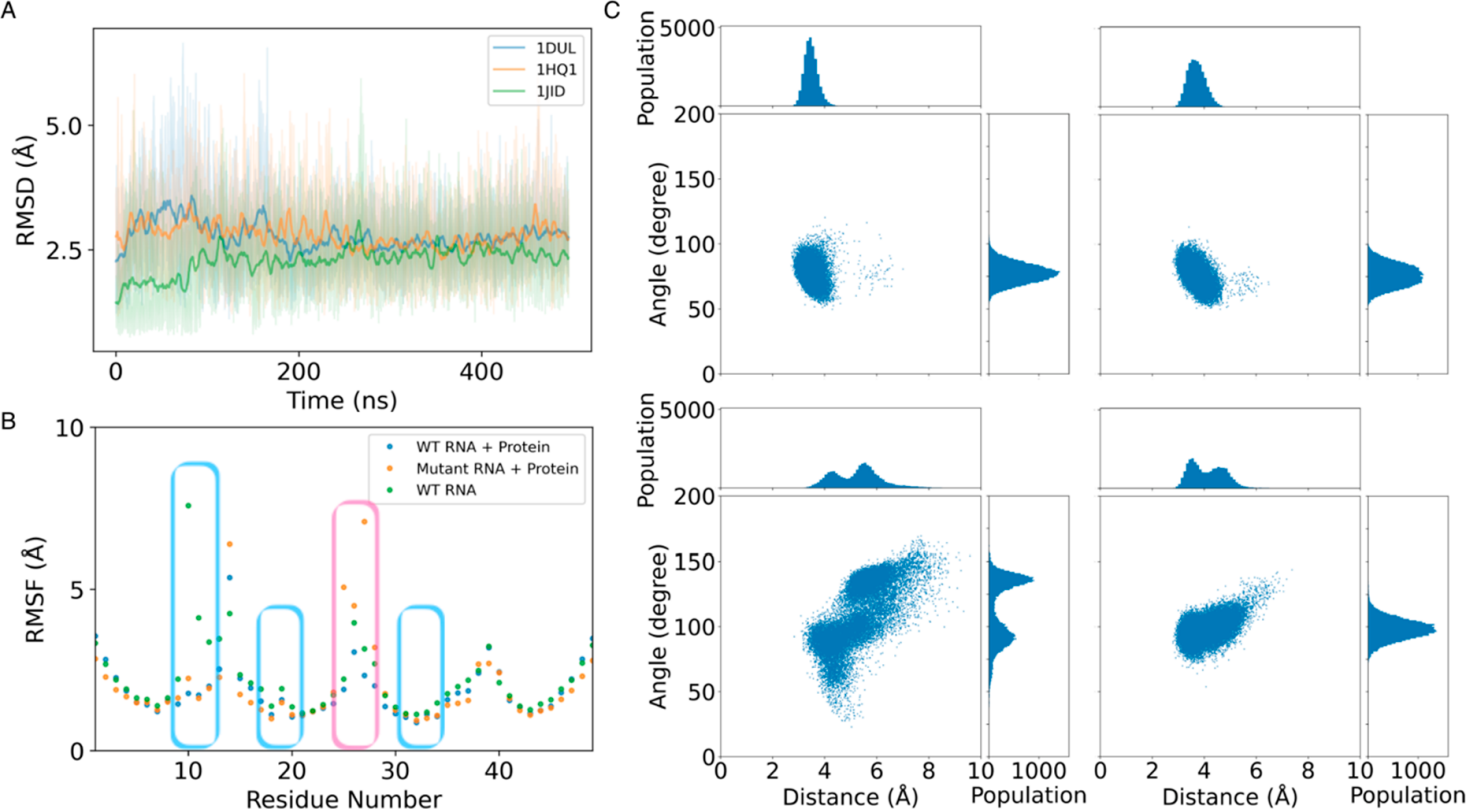
Tetraloop and hairpin stability. (A) RNA RMSD vs time for the three wild-type systems (RNA + protein). The plot shows the average (500 ps sliding window) as well as maximum and minimum values over three replicates for each system. (B) Side chain RMSF for the RNA residues for 1HQ1 in the presence and absence of protein (1HQ1-noPro) and with the G → C mutant sequence (1HQ1-G25C) (from one of the three replicates). The blue box identifies residues in contact with the protein and the red box the tetraloop region. The first residue in the tetraloop is the position where the mutation G → C is performed. (C) Ring center vs oxygen angle–distance correlations identify the presence of the anion−π interaction (∼3.7 Å, 90°).

The combination of force fields and molecular dynamics has often been used to describe noncanonical DNA and RNA structures for which the force fields were not initially parametrized. The success in representing those systems is a testament to the transferability of such potentials. Tetraloop motifs are ubiquitous in RNA structure as a building block during RNA folding and hairpin formation. They are also highly functional, involved in translation processes and have been proposed to mediate long-range RNA–RNA interactions.^33−36^ Recognition by proteins and RNA is believed to follow a shape complementarity mechanism in which the tetraloop plays an important role. The recognition is facilitated by the accessibility of the Watson and Crick hydrogen-bonding patterns in three of the bases in the tetraloop as well as by a determined backbone conformation.^34^ In recent years, the AMBER family of RNA force fields has seen significant changes to overcome known limitations for the modeling of RNA systems.^37−39^ While current force fields are not always able to fold all tetraloops correctly,^37^ they can correctly stabilize the native conformation. Triplicate sets of simulations starting from the experimental structure (see Figure 2) retained the global starting conformation for the RNA (see Figure S1, top), as well as the local details of the backbone (see Figure S2, top) and side chain (see Figure S3, top) tetraloop region. For 1HQ1 and 1DUL, the protein is bound far from the tetraloop region, whereas for 1J1D, the protein binding region encompasses the tetraloop (see Figure 2). To quantify the anion−π interaction stabilizing the tetraloop in finer detail, we monitored the phosphate oxygen to guanine ring (see Computational Methods) distance and orientation. All three systems showed the same distributions in both distance and orientation profiles (see Figure 3 and Figures S4 and S5). Correlations between angle and distances showed that shorter distances correlate with a 90° angle between the π-system of G and the phosphate group, as expected from a canonical anion−π interaction (see Figure S6).

The presence of the protein in the tetraloop region did not influence the RNA loop conformational preferences in 1JID with respect to the other two systems. We further tested the effect of simulating all three RNA systems in the absence of their binding partners. All three systems remained stable (see SI Figures S1 and S2) for the length of the trajectory with an overall similar RMSD to the initial simulations. However, RNA nucleotide fluctuations increased (see Figure 3b and Figure S6), especially in regions where the protein was previously bound. This is to be expected, reflecting the higher entropic freedom and flexibility of the free RNA (see SI Figure S1). The RMSF of the tetraloop in 1J1D remained stable despite the absence of the protein and the RMSF increased to similar ranges as in the other two systems (see Figures S7 and S8). The angle and distance distributions in the absence of the protein also behaved the same as when the protein was present (see Figures S4–6).

We further tested the ability of a guanine to cytosine mutation in the tetraloop region to disrupt the anion−π interaction in all three systems. We simulated all three systems with their protein binding partners in place. Although the overall RNA structure is maintained (see Figure S1), the structure of the tetraloop is significantly affected by this mutation (see Figures S2 and S3). While fluctuations in the backbone RMSD are small in magnitude, they represent distinct states, correlated with the larger changes in the side chain RMSD. Transitions happen beyond the 100 ns time scale, with some replicates being stable for the duration of the simulation (500 ns). However, once a transition happens, they are irreversible in the simulated time scales, compatible with more energetically favorable metastable states. To further quantify this effect, we have calculated the backbone torsional preferences (Figures S9–11) and entropy (see Computational Methods and Figure S12) of different dihedral parameters dictating backbone and side chain conformations. The analysis shows the greater conformational space available to the mutants (1DUL and 1HQ1) consistent with losing the tetraloop structure (see also Figure S6). For the 1JID case, the presence of the protein in the active site narrows down the conformational space, resulting in similar entropies before and after the mutation. Interestingly, for 1HQ1 we observe a large entropy increase for all bases in the tetraloop, while for 1DUL, only the two central bases have a significant behavior change. In all cases, the anion−π interaction is no longer present and the distance and angle distribution mapping the oxygen to the cytosine ring presents a very different distribution than the canonical case for 1DUL and 1HQ1 (see Figures S4–6). For these two systems, finding the anion near the ring is a low probability event, with the phosphate having a greater propensity to interact with the solvent environment than with the base. The case of 1J1D tells a different story: the presence of the protein forces the anion and π-ring to remain in close proximity. The interaction is stabilized by hydrogen bonds from Cys43, Arg60, and Arg127, which directly interact with both anion−π partners. In addition, the longer tail in the distance and angle distributions is reminiscent of 1HQ1 and 1DUL systems, and only the presence of the protein stops the tetraloop from following the same behavior. As a final analysis, we looked at the effect of the mutation in the conservation of protein–RNA contacts (see Figure S13). We notice that in 1HQ1 and 1DUL, where the protein binds far from the tetraloop region, the number of contacts fluctuates around the same values in the wild-type and mutant. For the case of 1JID, the mutation leads to a steady reduction of native contacts over the first 100 ns of simulation, most of which are not re-established in the simulation time. Taken together, the G to C mutation provokes a large backbone and side chain rearrangement in the current systems, which will likely affect shape recognition by proteins and long-range RNA interactions. Further tests on other tetraloops will reveal if this is a more generalizable property.

Despite the use of fixed point charged force fields, which cannot capture the polarization effects required to describe anion−π systems properly, we find that the force field is performing well at maintaining these interactions. To further analyze the interaction, we selected five representative structures randomly from the peak of the distance distribution for further assessment using a QM approach. We also identified structures representative of the extremes in the distance/angle anion−π distributions. Short distances were facilitated by the presence of Mg^2+^ or K^+^ ions at the interaction site, although these interactions were short-lived. At the opposite end, the anion−π interaction was disrupted by a water molecule between the anion and ring, which was stabilized through hydrogen bonds with the phosphate group. However, further analysis of the microsolvation environments did not reveal any high residence waters or ions near the tetraloop region.

### Quantum Mechanics Calculations

With the purpose to understand the energetics and directionality of the anion−π interaction, a series of MD snapshots were selected for QM calculations (see Supporting Information for details regarding the creation of the theoretical models). Each value gathered in Table 1 is given as an average of 5 snapshots, corresponding to short phosphate···G distances (denoted as “close”) and long phosphate···G distances (denoted as “far”). In addition, single point calculations on the X-ray crystal structures (using the same theoretical model, see Supporting Information) were also performed for comparison purposes. From the inspection of the results several interesting conclusions can be extracted.

**Table 1:** Average BSSE Corrected Anion−π Interaction Energy Values (Δ*E*_BSSE_, kcal/mol), Distances (*R*, Å), and Angles (*A* in deg) at the RI-MP2/def2-TZVPD Level of Theory Including RNA Bound (1HQ1, 1DUL, and 1JID) and Unbound (1HQ1-NP, 1DUL-NP, and 1JID-NP) Systems^a^

complex	Δ*E*_BSSE_	*R*^b^	*A*^c^
1HQ1			
X-ray	–3.1	3.082	75.1
1HQ1-close	–2.7	2.881	81.9
1HQ1-far	–1.0	4.325	93.2
1HQ1-NP-close	–2.9	2.859	84.9
1HQ1-NP-far	+0.5	4.224	95.0
1DUL			
X-ray	–3.8	3.1	76.4
1DUL-close	–2.2	2.886	82.5
1DUL-far	–0.9	3.771	86.6
1DUL-NP-close	–2.7	2.844	80.4
1DUL-NP-far	–0.3	3.802	93.1
1JID			
X-ray	–4.0	3.472	86.5
1JID-close	–4.3 (−8.3)	2.956 (2.944)	89.0 (89.4)
1JID-far	–0.7 (−3.6)	4.088 (4.005)	84.3 (84.8)
1JID-NP-close	–2.9 (−6.0)	2.839 (2.780)	83.8 (84.1)
1JID-NP-far	–0.3 (−1.3)	4.401 (4.375)	77.7 (78.2)

a The values of their respective X-ray structures are also indicated. Values in parentheses correspond to a geometry relaxation at the BP86-D3/def2-SVP//RI-MP2/def2-TZVPD level of theory.

b Distance measured from the closest O atom from the phosphate group to the 6-membered ring centroid.

c Angle measured including the closest O atom from the phosphate group, the 6-membered ring centroid, and the C4 atom from the guanine ring.

First, the anion−π interaction is favorable in all cases (except for 1HQ1-NP-far), ranging between −0.3 and −4.3 kcal/mol. Second, geometries tagged as “close” exhibit larger anion−π interaction energy values than their “far” analogous, as expected. Third, both the energetics and equilibrium distance of the anion−π interaction exhibit good agreement between the selected snapshots and the X-ray structures, thus indicating that the force field effectively samples conformations fluctuating around the initial X-ray crystal structure. This is observed in (i) 1JID structure, which achieved interaction energy values above (1JID-close, −4.3 kcal/mol) and below (1JID-far, −0.7 kcal/mol) those obtained for the X-ray structure, and (ii) 1HQ1, where the “close” conformation (1HQ1-close, −2.7 kcal/mol) obtained a similar value compared to the experimental structure (−3.1 kcal/mol). Finally, in 1DUL a more discrepant picture between the force field and the X-ray structure was obtained (−2.2 kcal/mol in 1DUL-close and −3.8 kcal/mol for 1DUL X-ray model).

While comparing both “close” and “far” dispositions an interesting picture is revealed. That is, in the “close” conformation of 1HQ1 and 1DUL structures, the anion−π interaction is slightly reinforced without the presence of the protein (e.g., −2.7 kcal/mol in 1HQ1-close and −2.9 kcal/mol in 1HQ1-NP-close). This is contrary to what is observed in case of 1JID, where the presence of the protein bound to RNA results in a strengthening of the anion−π interaction, in agreement with the results obtained from the MD simulations (−4.3 kcal/mol in 1JID-close and −2.9 kcal/mol in 1JID-NP-close). On the other hand, in all “far” geometries, the anion−π interaction is further stabilized when the protein is bound to the RNA molecule (e.g., −0.9 kcal/mol in 1DUL-far and −0.3 kcal/mol in 1DUL-NP-far).

Moreover, in the case of 1HQ1-NP and 1DUL-NP systems, we observed a more pronounced strengthening of the anion−π upon going from “far” to “close” conformations (ΔΔ*E* = −3.4 and −2.4 kcal/mol, respectively) compared to their protein-complexed analogous (ΔΔ*E* = −1.7 and −1.6 kcal/mol, respectively). The opposite behavior was obtained for 1JID complex (ΔΔ*E* 1JID-close/far = −3.6 kcal/mol, ΔΔ*E* 1JID-NP-close/far = −2.6 kcal/mol). Finally, the anion−π interaction angles range between 77.7° and 95°, thus exhibiting a certain directionally, being close to an ideal anion−π interaction (90°), as was also indicated in the MD study.

We finally optimized (BP86-D3/def2-SVP) and evaluated energetically (single points at RI-MP2/def2-TZVPD level of theory) a set of anion−π complexes corresponding to 1JID structure in order to analyze the quality of the structures gathered directly from the MD trajectory. To preserve the existence of the anion−π interaction, a set of distance and dihedral restraints was used (see Supporting Information for more details), and the results are gathered in Table 1. Interestingly, the behavior obtained is similar to that previously observed, that is, the anion−π interaction is strengthened by the presence of the protein (e.g., −8.3 kcal/mol in 1JID-close and −6.0 kcal/mol in 1JID-NP-close) and those geometries exhibiting “close” distances between the phosphate and the G ring achieved larger interaction energy values that their “far” analogous (e.g., −6.0 kcal/mol in 1JID-NP-close and −1.3 kcal/mol in 1JID-NP-far). The same picture is observed for the anion−π distances and angles, which lie within the same range as the ones retrieved directly from the MD snapshots without further geometry optimization.

### Additional Loop Interactions

**Figure 4:**
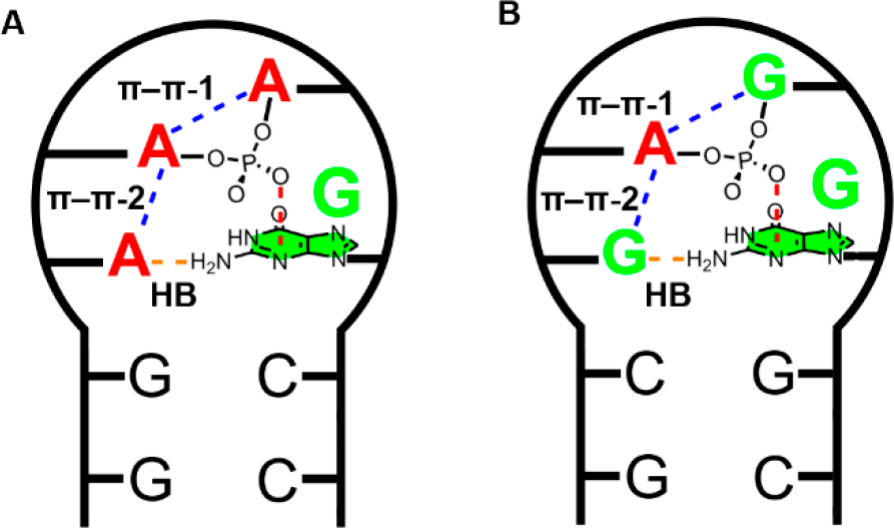
Additional base–base interactions in 1HQ1 (A) and 1JID (B) tetraloop structures. π–π-1 and 2 refer to stacking 1 (A–A and G–A) and 2 (A–A and A–G), in blue. HB refers to A–G and G–G base–base N–H···N hydrogen bonds (in orange).

We have also expanded our study to additional loop interactions between the A and G bases for 1HQ1 and 1JID structures (Figure 4). Concretely, we evaluated (i) the base stacking and (ii) the base hydrogen bonding interactions in the presence and absence of the protein using an average structure from the MD trajectory (see Supporting Information for Cartesian coordinates of the theoretical models used). The results are gathered in Table 2 and from their inspection several interesting conclusions can be extracted.

**Table 2:** BSSE Corrected Interaction Energy Values of Additional Loop Interactions (Δ*E*_BSSE_, kcal/mol) and Distances (*R*, Å) at the RI-MP2/def2-TZVPD Level of Theory Including RNA Bound (1HQ1 and 1JID) and Unbound (1HQ1-NP and 1JID-NP) Systems

complex	Δ*E*_BSSE_	*R*
1HQ1		
1HQ1-stacking-1	–8.4	3.652^a^
1HQ1-stacking-2	–6.5	3.576^a^
1HQ1-hydrogen bond	–3.4	2.395
1HQ1-NP-stacking-1	–6.7	3.538^a^
1HQ1-NP-stacking-2	–9.4	3.293^a^
1HQ1-NP-hydrogen bond	–4.9	2.271
1JID		
1JID-stacking-1	–7.5	3.684^a^
1JID-stacking-2	–7.7	3.658^a^
1JID-hydrogen bond	–3.7	2.287
1JID-NP-stacking-1	–5.8	3.866^a^
1JID-NP-stacking-2	–8.9	3.039^a^
1HQ1-NP-hydrogen bond	–5.3	2.293

a Shortest distance value between both rings.

In the case when the protein is bound to RNA, the 1HQ1-stacking-1 interaction achieved a larger interaction energy value than 1HQ1-stacking-2 complex, while in 1JID structure both stacking interactions achieved a similar strength. Additionally, for both systems the base stacking interactions showed more favorable interaction energy values than the N–H···N hydrogen bonds (e.g., −7.7 kcal/mol for 1JID-stacking-2 and −3.7 kcal/mol for 1JID-hydrogen bond). On the other hand, a different picture is observed upon removal of the protein, that is, complexes named as stacking-2 achieved a stronger interaction energy than their stacking-1 analogues, in agreement with a shorter base–base distances. Finally, the N–H···N hydrogen bond interaction is reinforced upon removal of the protein for both systems (e.g., −3.4 and −4.9 kcal/mol for 1HQ1-hydrogen bond and 1HQ1-NP-hydrogen bond, respectively), owing to a decrease in the intermolecular N–H···N distance.

### AIM and NCIplot Analyses

**Figure 5:**
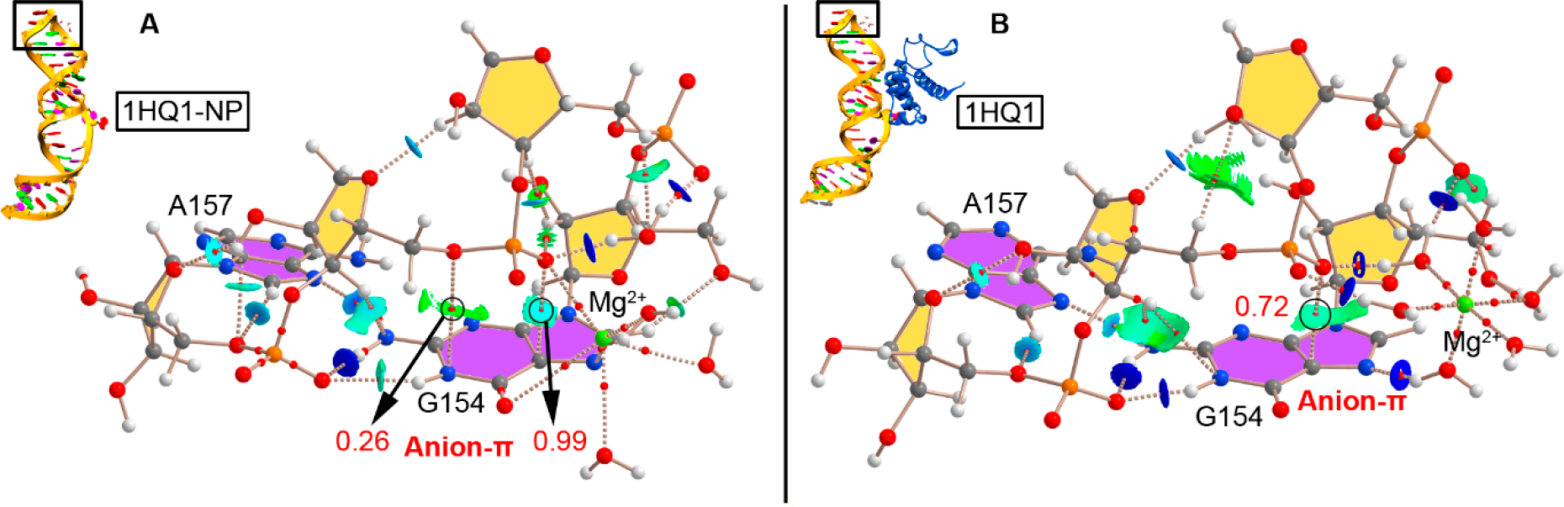
AIM distribution of bond critical points (BCPs in red spheres) and bond paths in (A) 1HQ1-NP and (B) 1HQ1 model structures. Only the first (G154) and last (A157) base of the tetraloop structure are shown, and only noncovalent BCPs were considered for sake of clarity. The values of density at the BCPs (ρ × 10^2^) characterizing the anion−π interaction (denoted in red) are also indicated in au. The NCIplot surfaces are also shown for both intra- and intermolecular interactions. NCIplot color range −0.02 au ≤ (sign λ_2_)ρ ≤ +0.02 au.

**Figure 6:**
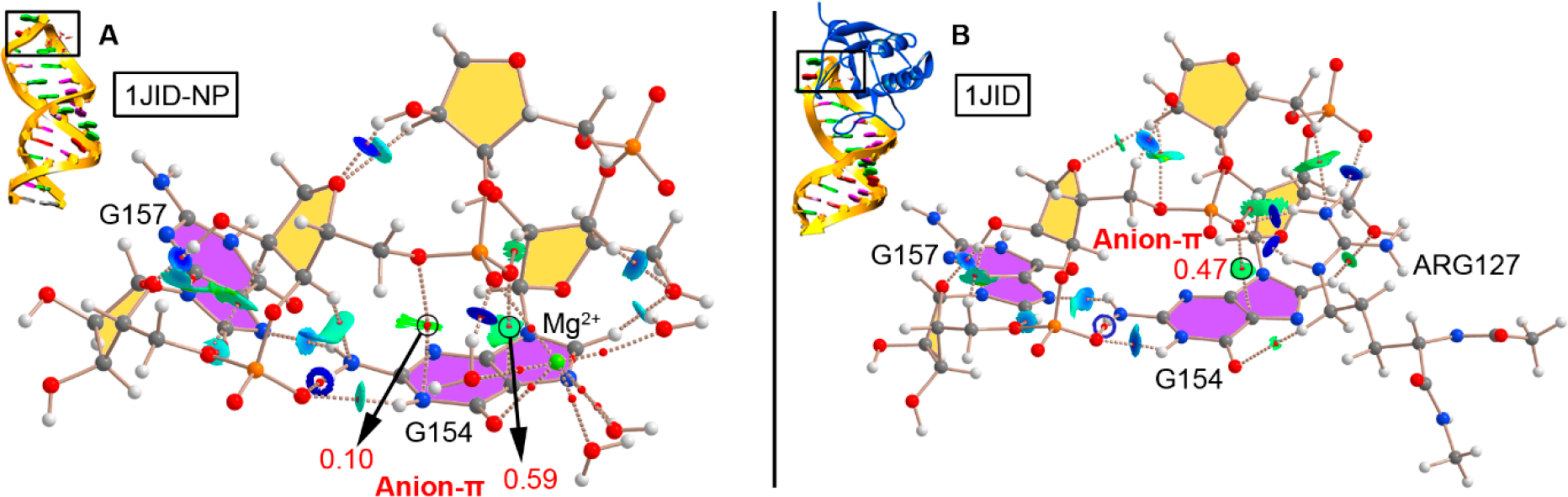
AIM distribution of bond critical points (BCPs in red spheres) and bond paths in (A) 1JID-NP and (B) 1JID model structures. Only the first (G154) and last (A157) bases of the tetraloop structure are shown and only noncovalent BCPs were considered for sake of clarity. The values of density at the BCPs (ρ × 10^2^) characterizing the anion−π interaction (denoted in red) are also indicated in au. The NCIplot surfaces are also shown for both intra- and intermolecular interactions. NCIplot color range −0.02 au ≤ (sign λ_2_)ρ ≤ +0.02 au.

To further complement the results derived from the energetic study, in Figure 5 and Figure 6 the AIM and NCIplot analyses for 1HQ1 and 1JID (both isolated RNA and protein bound) structures, respectively, are shown. These analyses were performed on average structures (see ESI for more details) from the whole trajectory, thus being useful to further characterize both the anion−π interaction and the interactions between the RNA tetraloop and the K^+^ and Mg^2+^ water clusters. In both analyses, the two tetraloop middle bases (A155–A156 in case of 1HQ1 and G155–A156 in case of 1JID) have been omitted for clarity purposes (see Figure S14 for AIM and NCIplot analyses regarding 1DUL structure).

As noted, in the case of 1HQ1-NP (Figure 5A), two bond critical points (BCPs) connect the phosphate O atoms with the G N1 and C5 atoms, thus characterizing the anion−π interaction. Also, the interaction between the RNA tetraloop and the Mg^2+^ water cluster is denoted by (i) the presence of two BCPs connecting an O atom from the phosphate moiety and an O from the carbonyl group of G to the Mg^2+^ ion, (ii) the presence of two BCPs connecting a Mg^2+^ coordinated water molecule and two phosphate O atoms (strong HBs) and (iii) a BCP connecting a −CH group from the RNA backbone with an O atom from a Mg^2+^ coordinated water (moderate HB).

On the other hand, once the protein is bound to the RNA (1HQ1 in Figure 5B), only one bond path connecting the O atom from the phosphate group and the C5 atom of G characterizes the anion−π interaction. In this case, an octahedral water cluster is formed, with no metal coordination from either the phosphate or the G base. Consequently, several strong HBs are established between the RNA assembly and the Mg^2+^ water cluster, which particularly involve (i) two O atoms from the phosphate moiety, (ii) the N1 atom from G, and (iii) a phosphate backbone group from RNA. QM calculations of these HBs resulted in an average interaction energy value of −52.8 kcal/mol per HB. This value is noticeably strong owing to the charged nature of both HB donor and acceptor partners. Other intramolecular interactions within the RNA assembly common to both systems mainly encompass HBs involving (i) two sugar moieties, (ii) G154-NH_2_ and A157-N7, and (iii) phosphate and N1 from G154.

In Figure 6, the AIM analysis of 1JID structure (both RNA isolated and protein bound) is shown. In the case of the 1JID-NP structure (Figure 6A), the anion−π interaction is characterized by the presence of two BCPs (similarly to 1HQ1-NP in Figure 5A) that connect the phosphate O atoms with the N1 and C5 atoms from G. In addition, metal coordination is also observed, with two bond paths connecting an O atom from the phosphate and an O atom from the carbonyl group of G with the Mg^2+^ ion, in a similar fashion to 1HQ1-NP. Finally, a BCP characterizes a strong HB established between a Mg^2+^ coordinated water molecule and an O atom from the phosphate group.

In the case of the protein–RNA complex (Figure 6B), an Arg127 residue is located in the vicinity of the RNA tetraloop and its guanidinium group is involved in a bifurcated HB with the phosphate group, as denoted by the two bond paths connecting the two −NH groups from the guanidinium moiety with an O atom from the phosphate group. In addition, two BCPs connect a −CH group from the Arg127 side chain and the −NH_2_ group from its guanidinium moiety with the carbonyl group of G and a −CH group from the RNA backbone, respectively, therefore characterizing the presence of two ancillary HBs. The computed strength of the HBs established between Arg127 and the RNA loop resulted in −56.5 kcal/mol per HB. Other intramolecular interactions within the RNA assembly common to 1JID-NP and 1JID systems mainly involve HBs involving (i) two sugar moieties, (ii) G154-NH_2_ and G157-N7, and (iii) phosphate and N1 from G154.

Finally, the NCIplot analyses of all four tetraloop structures were computed, owing to their usefulness to analyze noncovalent interactions accurately and unveil their location in real space. As noticed in Figure 5 and Figure 6, greenish and bluish isosurfaces indicate the nature and position of the noncovalent interactions established within the RNA tetraloop and between the RNA and either the metal water clusters or Arg127 moieties. In all cases, the anion−π interaction is described by a greenish surface between an O atom from the phosphate group and G, while strong HBs involving Mg^2+^ water clusters (1HQ1-NP, 1HQ1, and 1JID-NP) and Arg127 residue (1JID) are denoted by blue isosurfaces between both HB counterparts (involving −OH and −NH groups as donors and O and N atoms as acceptors). Lastly, in case of 1JID structure, the ancillary HBs are characterized by a greenish surface, indicating a weaker nature than those involving charged counterparts.

## Conclusions

In conclusion, we have evaluated the structural and energetic implications of phosphate···G anion−π interactions in GAAA and GGAG RNA tetraloops through a combined MD and QM study. Using as a case study three SRP–RNA complexes, MD simulations indicate that these tetraloops are highly stable, even in the absence of the protein bound to RNA. K^+^ and Mg^2+^ ions did not show remarkable residence times in either isolated or complexed RNA systems near the tetraloop region. Furthermore, mutation of the G base resulted in total disruption of both the HB base pair structure and the phosphate···G anion−π interaction. On the other hand, QM analyses (RI-MP2/def2-TZVPD level of theory) of selected MD snapshots revealed that anion−π interaction strength ranges between −0.3 and −4.3 kcal/mol and the interaction is directional in these systems. The energetic study was also extended to the additional noncovalent interactions present in the RNA tetraloop structure (π–π stacking and HB). Finally, the RNA tetraloops were further characterized by means of AIM and NCIplot analyses, being useful for characterizing the noncovalent partners involved in formation of the RNA assemblies as well as the intermolecular interactions with K^+^/Mg^2+^ water clusters and vicinal protein residues. We hope the findings gathered in this work will be useful for scientists working in the fields of RNA folding and engineering, as well as to increase the visibility of anion−π interactions among the protein–RNA community.

## Computational Methods

### PDB Inspection and Selection

A preliminary PDB survey was carried out for SRP-RNA complexes. As a result, 23 structures were found: 2PXQ, 2PXP, 2PXL, 2PXK, 2PXV, 2PXU, 2PXT, 2PXF, 2PXE, 2PXD, 2PXB, 1HQ1, 1MFQ, 1QZW, 1L9A, 1LNG, 1JID, 1DUL, 3UCZ, 3UD3, 3UD4, 3UCU, and 3NDB. From these, three systems (1HQ1, 1DUL, and 1JID) were selected for MD simulations owing to the high quality of the X-ray crystal structures (below 2 Å resolution).

### Molecular Dynamics Simulations

Initial structures were retrieved from the Protein Data Bank using PDB codes 1DUL, 1HQ1, and 1JID.^40^ We modeled unresolved residues in the protein for 1DUL and 1HQ1 using SwissModel, using 1HQ1 as a template for both.^41^ From each modeled PDB file, three systems were created (nine in total): (1) protein and RNA with its wild-type sequence; (2) protein and RNA with a guanine to cytosine mutation at position 25 for 1DUL and 1HQ1 and at position 13 for 1JID; (3) RNA-only with the wild-type sequence.

Proteins were treated with the ff19SB force field,^42^ while RNA was defined using the OL3 force field.^43,44^ The system was solvated in a cubic box using the TIP3P water model with a minimum 12 Å clearance between the edge of the macromolecules and any side of the box.^45^ Crystallographic waters and ions were retained. K^+^ and Cl^–^ ions^46^ were added to neutralize the system and reach a 150 mM concentration similar to physiological conditions. K^+^ and Mg^2+^ ions present in the experimental structure were included in the simulations.^47^ All systems were minimized in five stages with decreasing restraint weights of 25, 20, 15, 10, and 5 kcal/mol on heavy atoms. Each minimization stage comprised 1000 steps using the steepest descent algorithm^48^ followed by 1000 steps using the conjugate gradient algorithm.^49^ The systems were then gradually heated to 298.15 K using the Langevin thermostat over 50 ps and then equilibrated under NVT conditions for 950 ps with a time step of 2 fs and a collision frequency of 2.0 ps. We use SHAKE to constrain bonds involving hydrogen atoms.^50^ The systems were then subjected to 2 ns of equilibration under NPT conditions to stabilize the pressure using the Berendsen barostat with a relaxation time of 1.0 ps.^51^ The final production run was carried out using the GPU-enabled version of pmemd^52^ for 500 ns using the leapfrog integrator. Each simulation protocol was carried out in triplicate for each system. A 10 Å cutoff was used to approximate long-range electrostatic interactions using the particle mesh Ewald method.^53^ The first 50 ns of each simulation were disregarded as equilibration for analysis purposes unless otherwise specified. CPPTRAJ, MDTRAJ, VMD, and UCSF ChimeraX were employed for analysis and visualization.^54−57^

We monitored the anion−π interaction by looking at the distance between the oxygen in the phosphate facing the first guanine in the tetraloop and the center of mass of the heavy atoms in the 6 membered ring of guanine. We further looked at the orientation by using the angle defined by adding the C4 heavy atom on the ring to the atoms used in the distance calculation. Based on these distributions, we selected five structures close to the average value as well as representative cases of the extremes in the distribution. Finally, we analyzed high residency waters and ions by using VMD’s volumetric map tool, which analyzes the whole trajectory for the presence of ions or waters at each grid point. The program then calculates the density at each grid point and saves the results as a grid file to be used in data visualization.

#### Entropy Estimation

We characterize backbone torsion differences in the tetraloop region by using a simplified dimensionless measure of entropy (∑_*p*_ log *p*). We use 10° angle intervals for binning and add a plus one count on all bin to avoid log(0) issues in regions of dihedral space with no population.

### Quantum Mechanics Calculations

The energies of all complexes included in this study were computed at the RI-MP2^58^/def2-TZVPD^59^ level of theory. In case of calculations involving X-ray crystal structures, the H atoms were initially relaxed at the BP86^60^-D3^61^/def2-SVP^59^ level of theory to ensure a proper disposition prior to evaluating the interaction energy. In force field-derived structures, no H optimization was performed before the calculation of the interaction energies. On the other hand, in the case of the optimized 1JID set of structures, the BP86-D3/def2-SVP level of theory was used. The resulting geometries were used for single point calculations at the RI-MP2/def2-TZVPD level of theory. The calculations have been performed using the program TURBOMOLE, version 7.0.^62^ In addition, calculations for the molecular electrostatic potential (MEP) surfaces and wave function analysis have been carried out using Gaussian 16 software.^63^ The Bader’s “atoms in molecules” theory has been used to study the interactions discussed herein by means of the AIMall calculation package.^64^ The wave function analysis has been performed at the B3LYP/def2-TZVP level of theory. The NCIplot^65^ isosurfaces correspond to both favorable and unfavorable interactions, as differentiated by the sign of the second density Hessian eigenvalue and defined by the isosurface color. The color scheme is a red–yellow–green–blue scale with red for repulsive (ρ_cut_^+^) and blue for attractive (ρ_cut_^–^) NCI interaction density. Yellow and green surfaces correspond to weak repulsive and weak attractive interactions, respectively.

## Supporting Information Available

Additional data regarding MD (Figures S1 to S13) and QM calculations (AIM and NCIplot analyses of 1DUL structure and additional information on QM calculations) and Cartesian coordinates of anion−π complexes and of additional loop interactions (PDF)
