# Supplementary material for: Importance of Anion−π Interactions in RNA GAAA and GGAG Tetraloops: A Combined MD and QM Study
Source: J Chem Theory Comput. 2021 Sep 29;17(10):6624–33. doi: 10.1021/acs.jctc.1c00756 (PMC8515804; doi:10.1021/acs.jctc.1c00756)
Supplement: Supplementary file 1 — ct1c00756_si_001.pdf [file ct1c00756_si_001.pdf]

On the importance of anion-π interactions in RNA GAAA and GGAG tetraloops: A combined MD and QM study.

Reza Esmaeeli,[a] María de las Nieves Piña,[b] Antonio Frontera,[b] Alberto Pérez*,[a] Antonio Bauzá*,[b]

[a]Chemistry Department, University of Florida, Gainesville, FL 32611, USA; E-mail: perez@chem.ufl.edu; Tel.: +1-352 3927009
[b]Department of Chemistry, Universitat de les Illes Balears, Crta. de Valldemossa km 7.5, 07122 Palma (Baleares), SPAIN; Fax: (+) 34 971 173426; E-mail: antonio.bauza@uib.es

Electronic Supporting Information

Figure S1 Page 2
Figure S2 Page 2
Figure S3 Page 3
Figure S4 Page 3
Figure S5 Page 4
Figure S6 Page 5
Figure S7 Page 6
Figure S8 Page 6
Figure S9 Page 7
Figure S10 Page 7
Figure S11 Page 8
Figure S12 Page 8
Figure S13 Page 9
AIM and NCIplot analyses of 1DUL tetraloop structure Page 10
Additional information on QM calculations Page 11
Cartesian coordinates of anion-π models Page 15
Cartesian coordinates of additional loop interactions Page 68

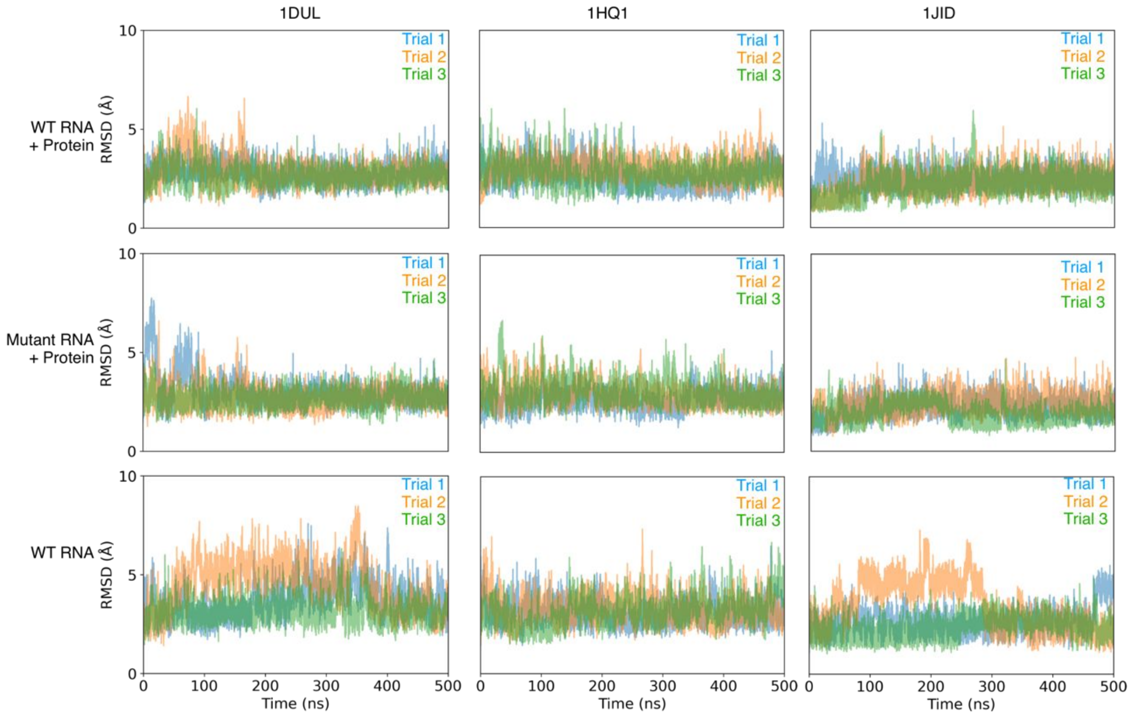

Figure S1. RMSD of RNA backbone for all simulations performed in this study. Each column represents a different system. Each system was performed in triplicates, showing that the overall structure of RNA was stable, but the removal of the protein increased events of higher RMSD in regions of bulge RNA.

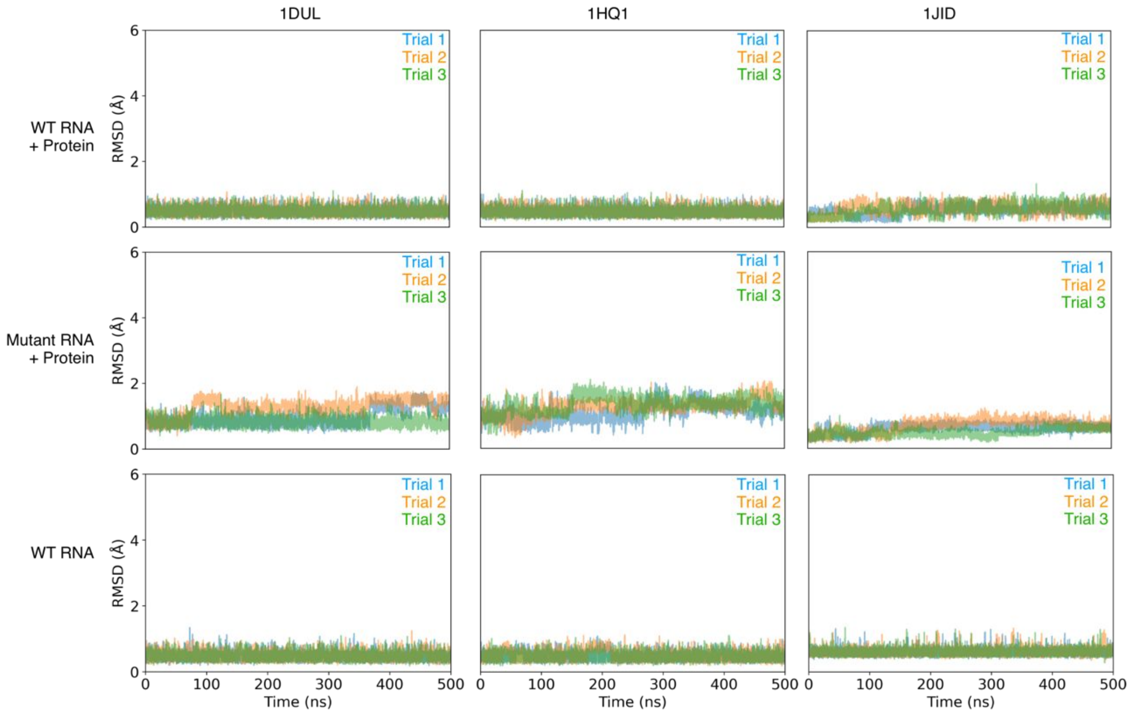

Figure S2. RMSD of RNA backbone for the tetraloop region. The G->C mutation in the tetraloop (middle panel) provokes a conformational change in the tetraloop region that is not observed in 1J1D, where the presence of the protein binding stabilizes the backbone of this tetraloop near its initial conformation.

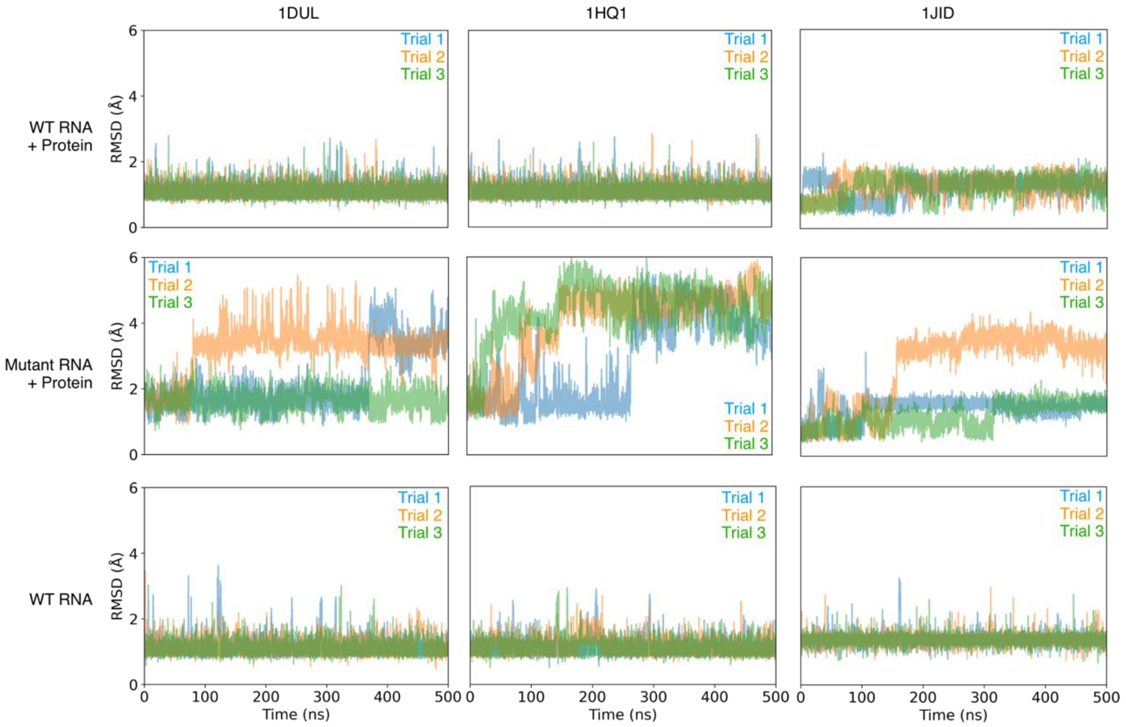

Figure S3. RMSD of RNA sidechain for the tetraloop region. The mutation of a G->C mutation in the tetraloop (middle panel) provokes a conformational change in the tetraloop region that is not observed in 1J1D. Despite keeping the RNA tetraloop backbone near its initial conformation (see FIG SI2)

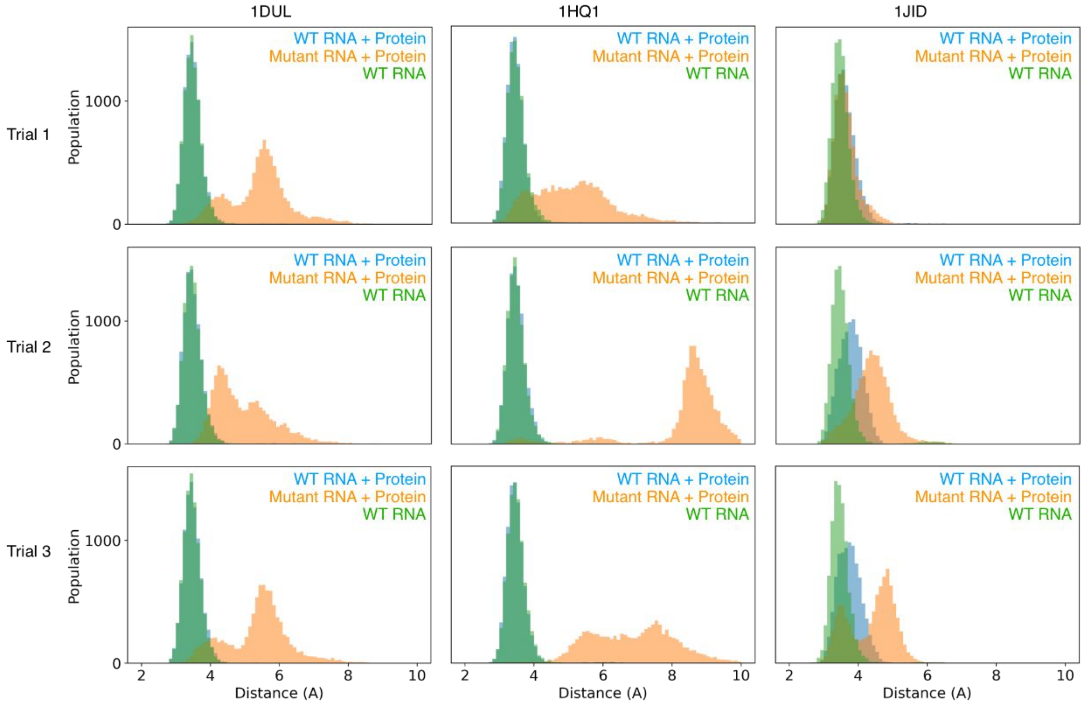

Figure S4. Anion-π distance distribution sampled during simulations. The G->C mutation of the interaction partner widened the distribution from the average of 3.5Å significantly. However, stabilization of the tetraloop structure by the protein environment in 1JID presented a narrower

distribution than the other two systems. Removal of the protein from canonical system had negligible impact on anion-π distance.

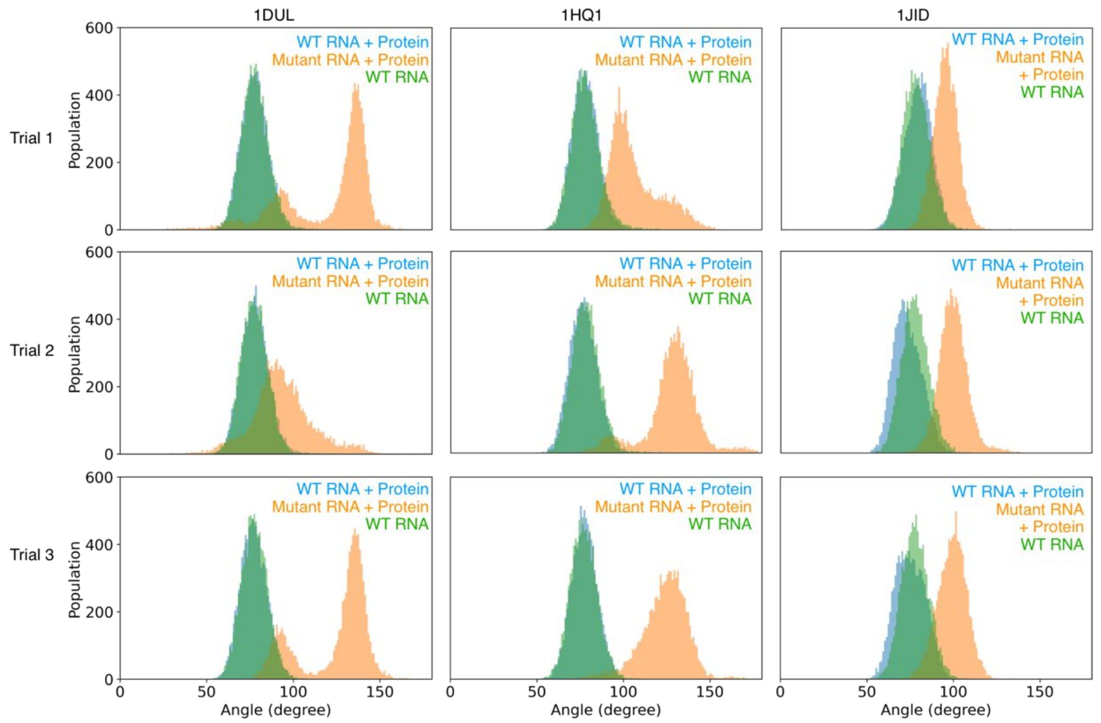

Figure S5. Anion-π angle distribution sampled during simulations. In canonical systems the angle of C4-ring center-OP2 was distributed around 80°. As a result of mutation, the distribution was focused on a higher angle, meaning the orientation of the anion-π partners was changed. Even in case of 1JID in which the conformation of the tetraloop was enforced by the protein, there is a shift in the orientation.

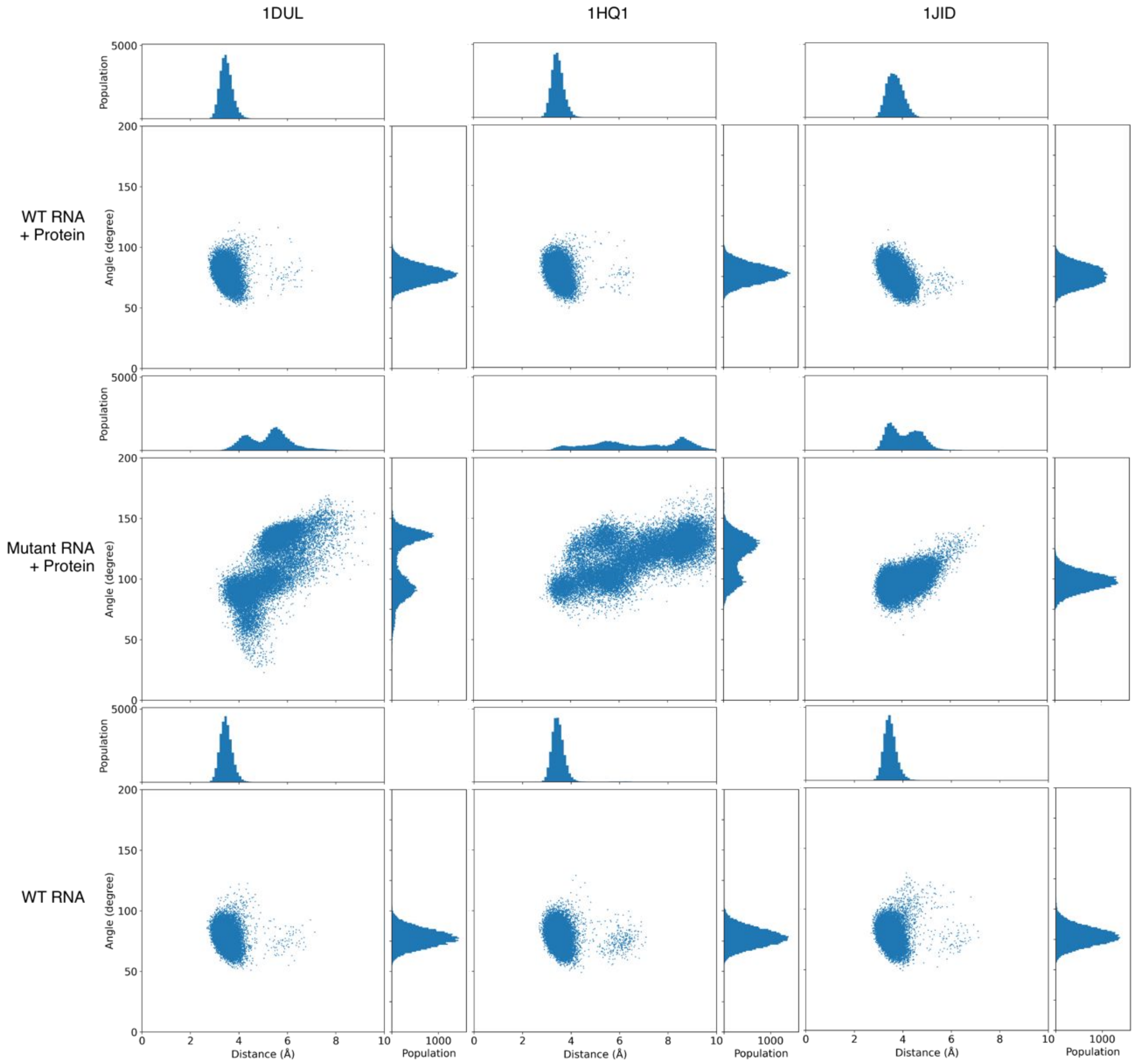

Figure S6. Angle-distance correlation of anion-π partners. Aggregated results from three independent runs for each panel. Mutation results in sampling of a larger angle and further distance in contrast to wild-type cases. There is a slight dispersion in the case of 1HQ1 WT RNA, suggesting more flexibility in the tetraloop.

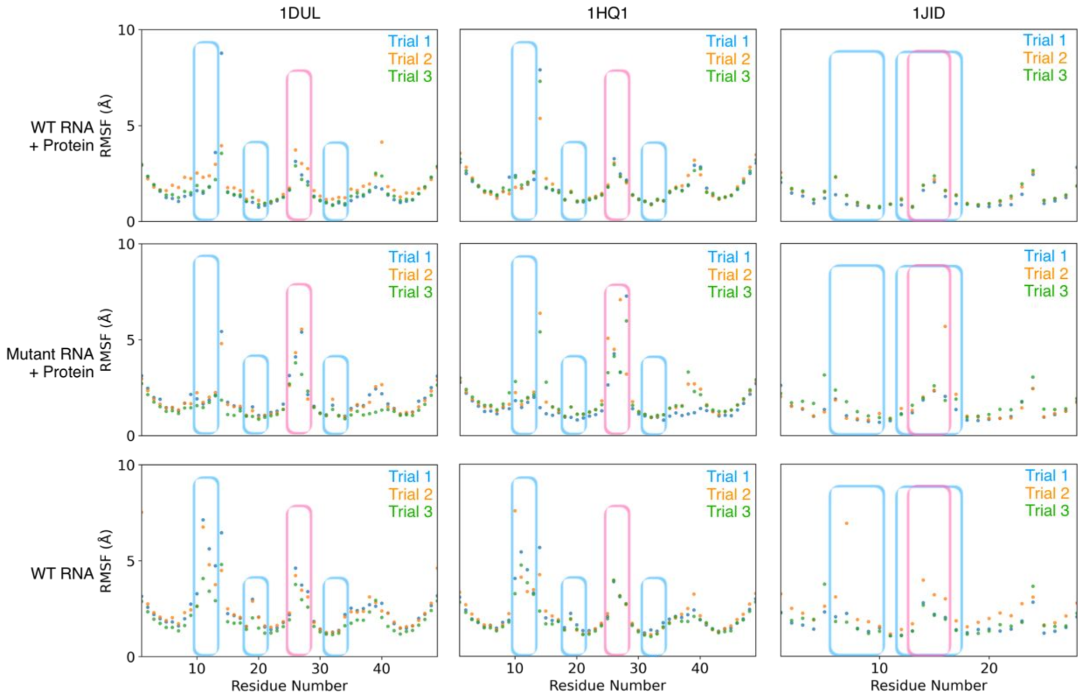

Figure S7 RMSF of RNA sidechains. Regions interacting with the protein are shown with blue rectangles and tetraloop is shown with a pink one. Mutation of G->C in the tetraloop leads to elevated fluctuations in that residue and its neighboring monomers. The impact of mutation is smaller in 1JID. Removal of protein causes instability in protein-interacting regions.

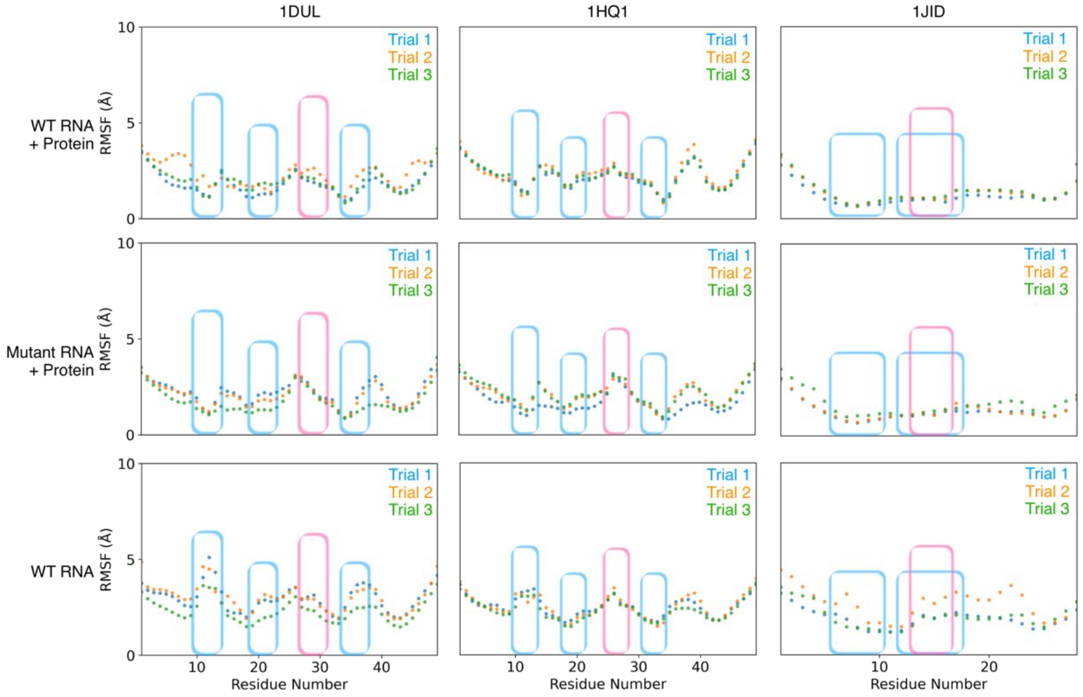

Figure S8. RMSF of RNA backbone. Protein interaction regions are marked with blue rectangles and the tetraloop is enclosed in a pink rectangle. The increase in backbone fluctuations due to mutation or protein removal is smaller than sidechains (see fig S9).

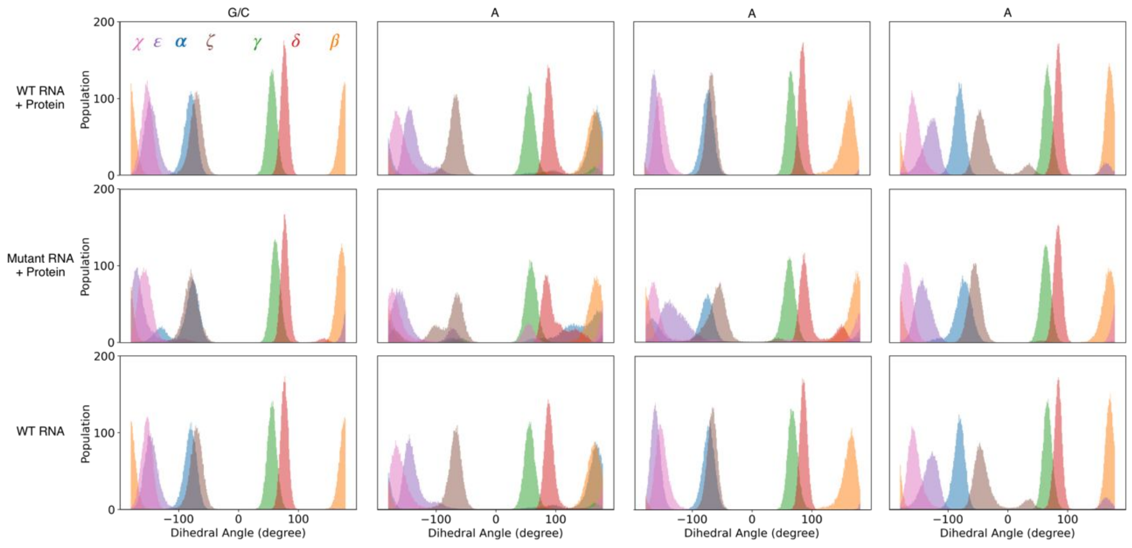

Figure S9. Backbone and Chi dihedral distributions. Results for 1DUL are pooled from 3 replicates and shown for each base in the tetraloop.

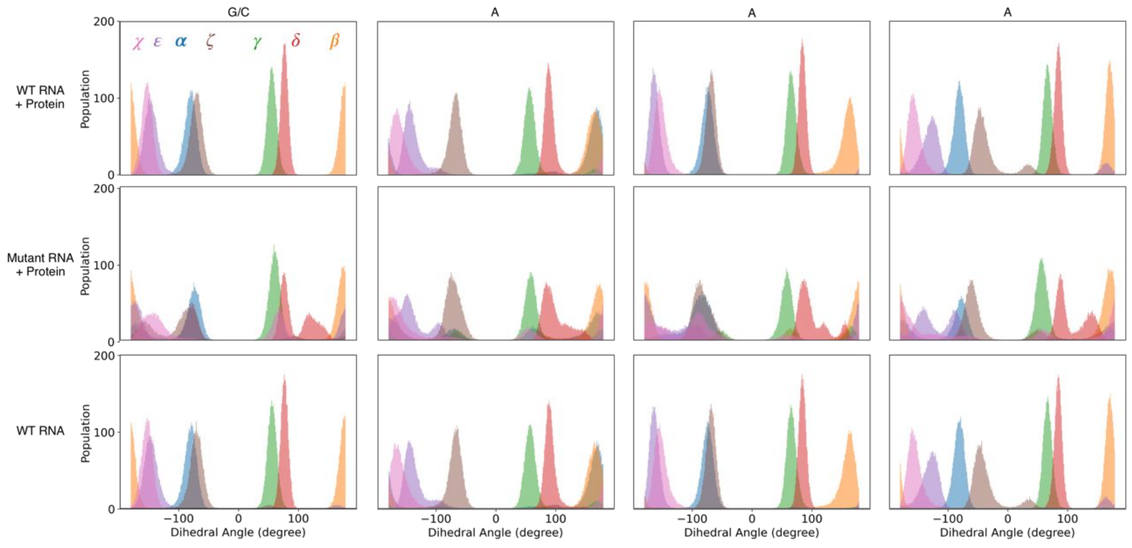

Figure S10. Backbone and Chi dihedral distributions. Results for 1HQ1 are pooled from 3 replicates and shown for each base in the tetraloop.

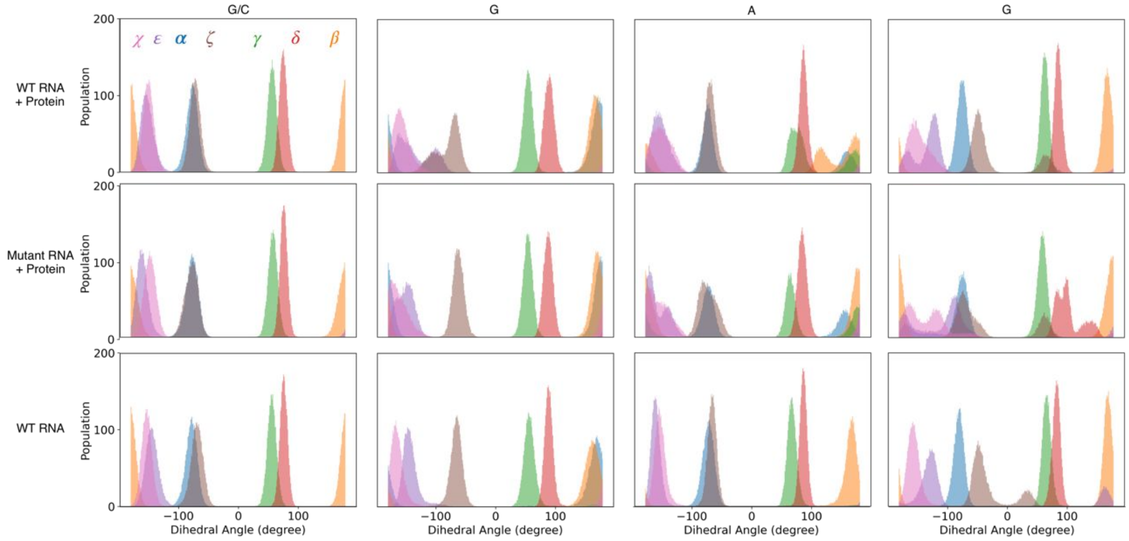

Figure S11. Backbone and Chi dihedral distributions. Results for 1JID are pooled from 3 replicates and shown for each base in the tetraloop.

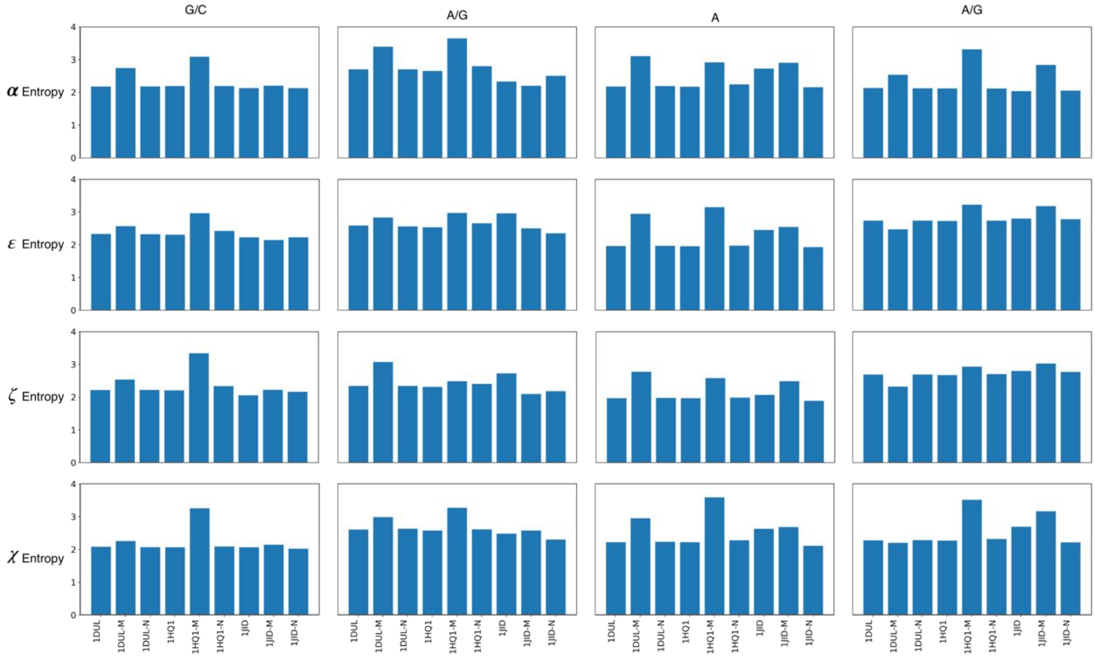

Figure S12. Dimensionless torsion entropy for selected dihedrals in each base in the tetraloop. The base names at the top indicate the difference in residues between the three systems – except for the first residue in the tetraloop, which indicates the site of mutation.

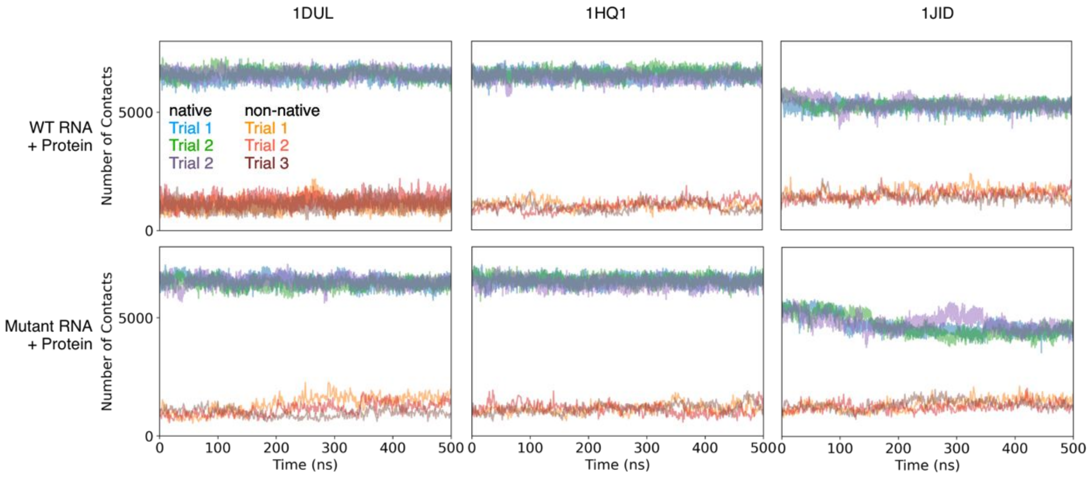

Figure S13. Atomic resolution fingerprint of protein-DNA contacts. The number of native contacts remain stable over the simulation timescale for all systems and replicates. The mutation in the tetraloop does not affect the contacts in 1DUL and 1HQ1 since the protein is interacting far away from the tetraloop region. However, for 1JID, where the protein interacts in the tetraloop site, we see a loss of native contacts and slight increase in non-native ones consistent with the loss of the anion-pi interaction.

AIM and NCIplot analyses of 1DUL tetraloop structure

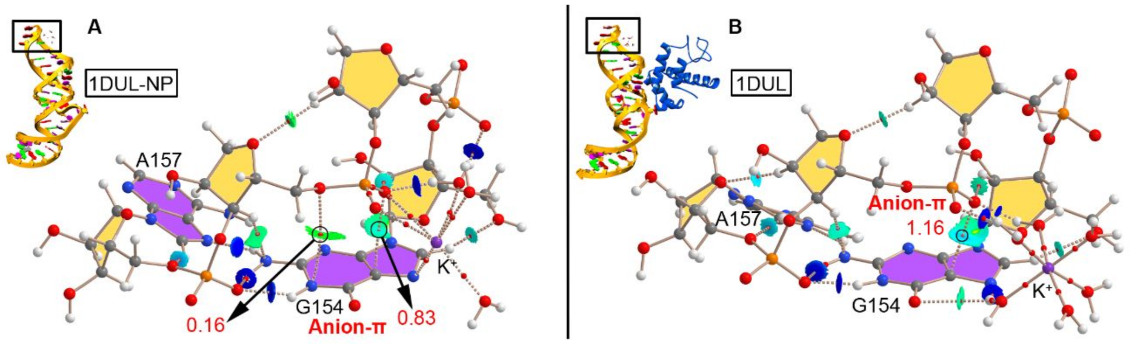

Figure S14. AIM distribution of bond critical points (BCP in red spheres) and bond paths in (A) 1DUL-NP and (B) 1DUL model structures. Only the first (G154) and last (A157) base of the tetraloop structure are shown and only noncovalent BCPs were considered for sake of clarity. The values of density at the BCPs ($\rho \cdot 10^2$) characterizing the anion-π interaction (denoted in red) are also indicated in a.u. The NCIplot surfaces are also shown for both intra-/intermolecular interactions. NCIplot colour range –0.02 a.u. ≤ (signλ2)ρ ≤ –0.02 a.u.

In case of 1DUL-NP (Fig. S14A), two BCPs and bond paths connect the phosphate O atoms with the G N1 and C5 atoms, thus characterizing the anion-π interaction. Also, the interaction between the RNA tetraloop with the K^+ water cluster is denoted by i) the presence of two BCPs connecting an O atom from the phosphate moiety and one BCP connecting an O from the carbonyl group of G to the K^+ ion, ii) the presence of two BCPs connecting a K^+ coordinated water molecule and two phosphate O atoms (strong HBs).

On the other hand, once the protein complexed to the RNA (1DUL in Fig. S14B), only one bond path connecting the O atom from the phosphate group and the C5 atom of G characterizes the anion-π interaction. In this case, an octahedral water cluster is formed, with no metal coordination from either the phosphate or the G base. Consequently, several strong HBs are established between the RNA assembly and the Mg^{2+} water cluster, which particularly involve i) two O atoms from the phosphate moiety and ii) the N7 and O atoms from G. QM calculations of these HBs resulted in an average interaction energy value of –34.4 kcal/mol per HB. This value is also remarkably strong owing to the charged nature of both HB donor and acceptor partners. Other intramolecular interactions within the RNA assembly common to both systems mainly encompass HBs involving i) two sugar moieties, ii) G154 $–NH_2$ and A157 N7 groups and iii) phosphate and N1 from G154.

Additional information on QM calculations:

Selected snapshots from the MD trajectories were used for the analysis of the anion-π interaction using QM calculations. More specifically, 10 snapshots were extracted from each trajectory (5 exhibiting close phosphate-guanine distances (denoted as CLOSE in Tables S1, S2 and S3) and 5 showing large phosphate-guanine distances (denoted as FAR in Tables S1, S2 and S3), see cartesian coordinates below). For each snapshot, a theoretical model was created including the phosphate group and the base following Scheme 1 and the interaction energy calculated at the RI-MP2/def2-TZVPD level of theory. In all complexes the global charge was set to -1. The values of the anion-π interactions given in Table 1 represent an average of the 5 QM models gathered in Tables S1, S2 and S3. In addition, theoretical models of i) selected average structures of the whole trajectory and ii) X-ray crystal structures of 1HQ1, 1DUL and 1JID were built for QM calculations also using the scheme shown in Fig. S14.

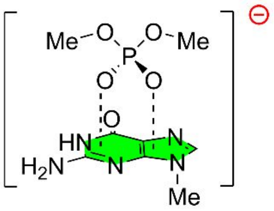

Figure S15. Schematic representation of the theoretical models used to compute the anion-π interaction energies.

Optimization of 1JID selected snapshots

The following constraints were used for the relaxation of the 1JID set of complexes:
Distances: **P**-**C5**/P-**N1**/P-**N3**
Dihedrals: **C6-N1-C2-N(NH_2)** and **C1-O2-O3-C4**
For those complexes tagged as “close” the **N7-C8-N9-C(Me)** dihedral was also kept frozen during optimization.

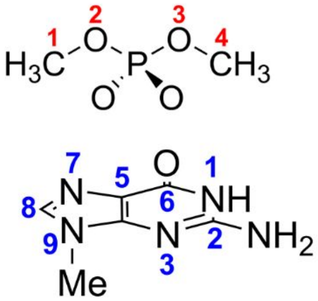

Figure S16. Schematic representation of both the phosphate and the guanine moieties, including labels to understand the geometric restraints imposed during optimization.

Table S1. List of structures used to compute the average anion-π interaction energy values, including both complexed (1HQ1) and isolated RNA (1HQ1-NP) sequences.

Complex	ΔE_{BSSE}	R[a]	A[b]
**1HQ1-483 (CLOSE)**	–4.9	2.932	87.1
**1HQ1-825 (CLOSE)**	+1.4	2.880	82.8
**1HQ1-1044 (CLOSE)**	–4.4	2.857	79.7
**1HQ1-1206 (CLOSE)**	–1.9	2.838	80.3
**1HQ1- 1537(CLOSE)**	–3.7	2.897	79.8
**1HQ1-28 (FAR)**	–1.9	3.549	88.3
**1HQ1-281 (FAR)**	–1.6	3.840	93.2
**1HQ1-1008 (FAR)**	–0.5	4.645	89.2
**1HQ1-1608 (FAR)**	–0.1	4.578	91.8
**1HQ1-1706 (FAR)**	–1.1	5.015	103.7
**1HQ1-NP-919 (CLOSE)**	–2.5	2.868	84.6
**1HQ1-NP-968 (CLOSE)**	–4.8	2.850	84.7
**1HQ1-NP-1418 (CLOSE)**	–2.9	2.854	80.4
**1HQ1-NP-1955 (CLOSE)**	–0.9	2.862	89.0
**1HQ1-NP-2648 (CLOSE)**	–3.6	2.861	86.0
**1HQ1-NP-1292 (FAR)**	–0.3	4.350	107.5
**1HQ1-NP-1591 (FAR)**	+1.0	4.349	95.5
**1HQ1-NP-2366 (FAR)**	+1.4	3.849	76.4
**1HQ1-NP-3633 (FAR)**	–1.0	3.462	76.2
**1HQ1-NP-3833 (FAR)**	+1.3	5.109	119.5

[a]Distance measured from the closest O atom from the phosphate group to the 6-membered ring centroid.

[b]Angle measured including the closest O atom from the phosphate group, the 6-membered ring centroid and C4 atom from guanine ring.

Table S2. List of structures used to compute the average anion-π interaction energy values, including both complexed (1DUL) and isolated RNA (1DUL-NP) sequences.

Complex	ΔE_{BSSE}	R^a	A^b
**1DUL-4 (CLOSE)**	–1.9	2.940	91.4
**1DUL-598 (CLOSE)**	–3.2	2.720	83.0
**1DUL-1140 (CLOSE)**	–4.1	2.920	82.1
**1DUL-1449 (CLOSE)**	–1.4	2.911	79.4
**1DUL-1908 (CLOSE)**	–0.2	2.938	76.8
**1DUL-1492 (FAR)**	–0.5	3.815	73.5
**1DUL-1777 (FAR)**	–0.5	3.268	70.3
**1DUL-1651 (FAR)**	–1.1	4.458	107.6
**1DUL-2682 (FAR)**	+0.4	3.933	98.3
**1DUL-3184 (FAR)**	–2.5	3.382	83.4
**1DUL-NP-344 (CLOSE)**	–3.2	2.743	90.9
**1DUL-NP-487 (CLOSE)**	–5.8	2.868	74.9
**1DUL-NP-672 (CLOSE)**	–2.8	2.874	79.0
**1DUL-NP-1338 (CLOSE)**	–2.4	2.855	72.0
**1DUL-NP-1425 (CLOSE)**	+0.4	2.882	85.4
**1DUL-NP-41 (FAR)**	+0.2	3.386	90.8
**1DUL-NP-1314 (FAR)**	+0.1	3.932	98.0
**1DUL-NP-1923 (FAR)**	+0.3	4.144	87.8
**1DUL-NP-1658 (FAR)**	–2.9	3.371	97.7
**1DUL-NP-2489 (FAR)**	+0.6	4.177	91.3

[a]Distance measured from the closest O atom from the phosphate group to the 6-membered ring centroid.

[b]Angle measured including the closest O atom from the phosphate group, the 6-membered ring centroid and C4 atom from guanine ring.

Table S3. List of structures used to compute the average anion-π interaction energy values, including both complexed (1JID) and isolated RNA (1JID-NP) sequences. Values in parenthesis were obtained through geometry optimization at the BP86-D3/def2-SVP//RIMP2/def2-TZVPD level of theory.

Complex	ΔE_{BSSE}	R[a]	A[b]
**1JID-38 (CLOSE)**	−3.3 (−6.7)	2.994 (2.995)	84.7 (90.6)
**1JID-492 (CLOSE)**	−6.3 (−9.3)	2.980 (2.741)	91.8 (84.8)
**1JID-966 (CLOSE)**	−6.1 (−8.9)	2.973 (2.875)	90.6 (95.5)
**1JID-1438 (CLOSE)**	−3.1 (−8.7)	3.015 (3.168)	91.4 (86.5)
**1JID-1877 (CLOSE)**	−2.6 (−8.0)	2.817 (2.939)	86.8 (89.5)
**1JID-96 (FAR)**	−1.4 (−2.1)	4.422 (4.278)	67.9 (67.2)
**1JID-764 (FAR)**	+1.2 (−3.4)	4.333 (4.152)	83.3 (84.6)
**1JID-816 (FAR)**	−1.9 (−4.7)	3.401 (3.301)	99.2 (106.5)
**1JID-840 (FAR)**	+0.1 (−3.9)	3.893 (3.906)	92.0 (90.2)
**1JID-1037 (FAR)**	−1.4 (−4.1)	4.392 (4.386)	79.3 (75.5)
**1JID-NP-1318 (CLOSE)**	−4.4 (−8.2)	2.864 (2.734)	91.1 (92.5)
**1JID-NP-1396 (CLOSE)**	−1.7 (−4.1)	2.852 (2.778)	75.5 (80.9)
**1JID-NP-2284 (CLOSE)**	−2.9 (−6.0)	2.853 (2.772)	85.4 (85.2)
**1JID-NP-3652 (CLOSE)**	−3.0 (−4.1)	2.820 (2.778)	85.3 (80.9)
**1JID-NP-5743 (CLOSE)**	−2.5 (−7.4)	2.808 (2.838)	81.6 (80.9)
**1JID-NP-4787 (FAR)**	−1.1 (−1.4)	4.440 (4.435)	69.7 (70.2)
**1JID-NP-4788 (FAR)**	−1.3 (−1.5)	4.606 (4.592)	80.5 (81.2)
**1JID-NP-4802 (FAR)**	+0.5 (−1.6)	4.201 (4.167)	72.8 (71.5)
**1JID-NP-4806 (FAR)**	+0.9 (−1.5)	4.342 (4.277)	92.0 (95.0)
**1JID-NP-5975 (FAR)**	−0.3 (−0.5)	4.418 (4.403)	73.4 (73.1)

[a]Distance measured from the closest O atom from the phosphate group to the 6-membered ring centroid.

[b]Angle measured including the closest O atom from the phosphate group, the 6-membered ring centroid and C4 atom from guanine ring.

Cartesian coordinates of anion-π models

X-ray structures

1HQ1

C	2.87718590	-2.40886300	-0.68133750
N	2.80518590	-0.93386300	-0.71933750
C	3.28618590	-0.07286300	-1.67733750
N	2.98718590	1.16813700	-1.38233750
C	2.26718590	1.13013700	-0.19433750
C	1.69218590	2.15513700	0.60266250
O	1.69318590	3.37313700	0.40666250
N	1.03918590	1.66013700	1.73366250
C	0.96218590	0.33613700	2.04766250
N	0.29918590	0.01313700	3.16966250
N	1.50318590	-0.64386300	1.32066250
C	2.14118590	-0.17486300	0.23466250
C	-2.94281410	-1.16486300	-2.27333750
O	-2.93781410	0.22213700	-1.93633750
P	-1.78781410	0.73213700	-0.92133750
O	-1.95181410	2.22313700	-0.97333750
O	-0.46481410	0.15213700	-1.25133750
O	-2.29381410	0.27113700	0.53066250
C	-3.61781410	0.68013700	0.98266250
H	2.47871860	-2.72936630	0.29944330
H	3.84342170	-0.42364900	-2.55672780
H	0.46657540	0.64292888	3.96098215
H	-1.90000070	-1.54075510	-2.37634140
H	-3.75840890	1.75683180	0.74119890
H	-4.41635150	0.08993240	0.47975020
H	-3.67310130	0.50870530	2.07894270
H	-3.45997420	-1.78725650	-1.50062200
H	-3.48319740	-1.29866200	-3.23672240
H	2.25282240	-2.85555280	-1.48238700
H	3.92506410	-2.75614110	-0.79203170
H	0.49077779	-0.95705710	3.43460615
H	0.55021480	2.37388500	2.27890280

1DUL

C	2.80097740	-2.41990300	-0.75114560
N	2.78097740	-0.96090300	-0.72714560
C	3.27797740	-0.08890300	-1.66814560
N	2.99397740	1.16009700	-1.40614560
C	2.29097740	1.10709700	-0.20914560
C	1.68897740	2.13909700	0.55485440
O	1.64697740	3.35609700	0.31585440
N	1.07097740	1.63809700	1.69485440
C	1.02497740	0.31309700	2.05185440
N	0.36297740	0.02409700	3.18085440
N	1.58097740	-0.65990300	1.34785440
C	2.18397740	-0.19490300	0.23685440
C	-2.98202260	-1.12090300	-2.29014560
O	-2.88102260	0.26409700	-1.96414560
P	-1.80202260	0.75909700	-0.87214560
O	-1.87102260	2.25009700	-0.84714560
O	-0.51102260	0.08909700	-1.19414560
O	-2.36002260	0.24009700	0.52785440
C	-3.61402260	0.71009700	1.02085440
H	2.86130770	-2.79773330	0.28771310
H	3.81602870	-0.45912170	-2.55335830

H	0.53092917	0.68066994	3.95016495
H	-1.96615450	-1.57142080	-2.37213740
H	-3.66802590	1.81808290	0.91364350
H	-4.47014340	0.26631890	0.45855130
H	-3.70615470	0.42110960	2.09130490
H	-3.55269290	-1.69479630	-1.51730950
H	-3.51708050	-1.21952080	-3.26033080
H	1.86852600	-2.81038030	-1.21013500
H	3.67658690	-2.77500940	-1.32909170
H	0.55193724	-0.93863938	3.47396602
H	0.54636210	2.34240740	2.21985200

1JID

C	-0.96260040	-3.48751930	-0.47066920
N	-1.67560040	-2.38351930	-1.12566920
C	-3.04260040	-2.17151930	-1.27466920
N	-3.32860040	-0.96651930	-1.73166920
C	-2.07860040	-0.39251930	-1.94266920
C	-1.71760040	0.89248070	-2.45066920
O	-2.47460040	1.81648070	-2.89666920
N	-0.33060040	1.05848070	-2.44166920
C	0.60239960	0.10648070	-2.07866920
N	1.94039960	0.47248070	-2.13866920
N	0.28239960	-1.11251930	-1.66766920
C	-1.05960040	-1.28051930	-1.60266920
C	0.67539960	0.33548070	4.20033080
O	0.00139960	1.47448070	3.65733080
P	0.19939960	1.83348070	2.12833080
O	-0.66560040	3.01748070	1.82133080
O	0.04439960	0.63648070	1.27133080
O	1.72039960	2.29048070	2.18533080
C	2.56039960	2.25348070	1.03633080
H	-0.17291880	-3.88175350	-1.13990650
H	-3.76924950	-2.93039080	-0.95158920
H	2.52063334	-0.37047918	-2.08932354
H	0.63008960	-0.51706080	3.48356860
H	2.54103640	1.24098950	0.56650470
H	2.23108070	2.99834160	0.26755290
H	1.74986310	0.55872990	4.40886910
H	0.17940820	0.05042090	5.15348650
H	3.59817250	2.50546390	1.34461050
H	-1.68220270	-4.29133220	-0.22660900
H	-0.48463110	-3.11426830	0.45738130
H	2.17041282	1.02803055	-2.97016582
H	-0.02462090	1.98879230	-2.74007690

Average structures

1DUL-758

C	55.78300000	26.43400000	26.49100000
H	55.86300000	25.64900000	27.24300000
N	54.44400000	27.07000000	26.45800000
C	54.17100000	28.29000000	25.87600000
H	54.98000000	28.90000000	25.50200000
N	52.94000000	28.66600000	25.87100000
C	52.31400000	27.45900000	26.37000000
C	50.92300000	27.09000000	26.54900000
O	49.90400000	27.65800000	26.21900000

N	50.72500000	25.98900000	27.32400000
H	49.79700000	25.61800000	27.46800000
C	51.70900000	25.20600000	27.81800000
N	51.39900000	24.13700000	28.44400000
H	50.47100000	23.87400000	28.74500000
H	52.17700000	23.53900000	28.68100000
N	52.99000000	25.42600000	27.49600000
C	53.23800000	26.60200000	26.89400000
C	53.62800000	24.43000000	21.79600000
H	54.12100000	24.42800000	22.76800000
O	52.49400000	25.24100000	21.61900000
P	51.86400000	25.93800000	22.91700000
O	50.73100000	26.79200000	22.44200000
O	52.94000000	26.56100000	23.66100000
O	51.19100000	24.72000000	23.79300000
C	49.95500000	24.16200000	23.46600000
H	49.78600000	23.99300000	22.40300000
H	49.17000000	24.76900000	23.91700000
K	52.03900000	30.42600000	22.58500000
O	53.44800000	28.84600000	22.76300000
H	54.35100000	28.89200000	22.45100000
H	53.43900000	28.09900000	23.36200000
O	50.86300000	29.27600000	21.52300000
H	50.87600000	28.32600000	21.64000000
H	50.20500000	29.42500000	20.84400000
O	50.95700000	29.99000000	24.27300000
H	50.10100000	30.25200000	24.61200000
H	51.35200000	29.48400000	24.98300000
O	52.90200000	30.84500000	20.85000000
H	53.71400000	31.33200000	20.71100000
H	52.53300000	30.73900000	19.97300000
O	53.48600000	31.63900000	23.41100000
H	54.40244650	31.54519146	23.14095228
O	50.90600000	32.22600000	22.31100000
H	50.00000000	32.52300000	22.23100000
H	51.33500000	32.90800000	22.82900000
H	53.24357760	32.29783358	24.06580508
H	54.26231012	24.47565373	20.93549642
H	53.28703195	23.42529947	21.65737233
H	49.78659064	23.22047086	23.94564692
H	55.96022476	25.95507119	25.55072420
H	56.57542948	27.12569692	26.68724195

1DUL-NP-2171

C	20.43700000	25.53400000	45.51500000
H	20.35736587	26.39736186	44.88797153
N	21.61300000	24.74700000	45.11800000
C	22.35500000	23.84600000	45.84600000
H	22.22000000	23.82100000	46.91700000
N	23.33300000	23.24400000	45.17300000
C	23.10100000	23.58600000	43.89100000
C	23.66700000	23.14800000	42.63300000
O	24.57400000	22.33000000	42.44600000
N	23.28100000	23.96100000	41.57400000
H	23.71600000	23.77800000	40.68100000
C	22.21500000	24.80300000	41.65300000
N	21.86700000	25.34700000	40.52900000
H	22.40500000	25.04100000	39.73100000
H	21.20500000	26.10300000	40.43200000
N	21.61800000	25.21700000	42.75200000

C	22.10100000	24.58700000	43.84900000
C	18.13400000	20.18200000	43.49200000
H	18.26800000	21.22800000	43.76600000
O	19.39000000	19.65000000	43.15000000
P	20.63200000	20.60500000	42.87600000
O	21.78500000	19.70800000	42.78300000
O	20.63200000	21.63900000	43.96800000
O	20.48300000	21.28200000	41.41200000
C	20.78700000	20.47600000	40.19400000
H	20.31400000	19.49500000	40.23600000
H	21.84600000	20.23500000	40.09700000
K	22.92100000	20.54400000	45.20600000
O	20.62800000	21.05800000	46.60100000
H	19.89600000	21.27900000	47.17600000
H	20.41700000	21.48400000	45.77000000
O	21.92400000	17.87000000	45.71100000
H	20.96700000	17.88300000	45.70300000
H	22.15500000	17.86400000	46.64000000
O	24.81700000	18.40800000	44.31700000
H	24.56100000	18.61100000	43.41700000
H	25.72600000	18.11600000	44.24700000
O	25.11300000	20.84100000	46.80700000
H	25.19600000	21.40900000	47.57300000
H	25.87700000	21.04900000	46.27000000
H	17.45632011	19.78074217	44.21632183
H	17.50896825	20.11977648	42.62576473
H	20.29690294	20.83058312	39.31140401
H	19.55596717	24.93566440	45.41167691
H	20.54147953	25.84194951	46.53438762

1HQ1-694

C	20.43700000	25.53400000	45.51500000
H	20.35736587	26.39736186	44.88797153
N	21.61300000	24.74700000	45.11800000
C	22.35500000	23.84600000	45.84600000
H	22.22000000	23.82100000	46.91700000
N	23.33300000	23.24400000	45.17300000
C	23.10100000	23.58600000	43.89100000
C	23.66700000	23.14800000	42.63300000
O	24.57400000	22.33000000	42.44600000
N	23.28100000	23.96100000	41.57400000
H	23.71600000	23.77800000	40.68100000
C	22.21500000	24.80300000	41.65300000
N	21.86700000	25.34700000	40.52900000
H	22.40500000	25.04100000	39.73100000
H	21.20500000	26.10300000	40.43200000
N	21.61800000	25.21700000	42.75200000
C	22.10100000	24.58700000	43.84900000
C	18.13400000	20.18200000	43.49200000
H	18.26800000	21.22800000	43.76600000
O	19.39000000	19.65000000	43.15000000
P	20.63200000	20.60500000	42.87600000
O	21.78500000	19.70800000	42.78300000
O	20.63200000	21.63900000	43.96800000
O	20.48300000	21.28200000	41.41200000
C	20.78700000	20.47600000	40.19400000
H	20.31400000	19.49500000	40.23600000
H	21.84600000	20.23500000	40.09700000
K	22.92100000	20.54400000	45.20600000
O	20.62800000	21.05800000	46.60100000

H	19.89600000	21.27900000	47.17600000
H	20.41700000	21.48400000	45.77000000
O	21.92400000	17.87000000	45.71100000
H	20.96700000	17.88300000	45.70300000
H	22.15500000	17.86400000	46.64000000
O	24.81700000	18.40800000	44.31700000
H	24.56100000	18.61100000	43.41700000
H	25.72600000	18.11600000	44.24700000
O	25.11300000	20.84100000	46.80700000
H	25.19600000	21.40900000	47.57300000
H	25.87700000	21.04900000	46.27000000
H	17.45632011	19.78074217	44.21632183
H	17.50896825	20.11977648	42.62576473
H	20.29690294	20.83058312	39.31140401
H	19.55596717	24.93566440	45.41167691
H	20.54147953	25.84194951	46.53438762

1HQ1-NP-784

C	51.62100000	14.39000000	35.50200000
H	52.31000000	15.18800000	35.22400000
N	51.35200000	14.67500000	36.97400000
C	50.18200000	14.49500000	37.64900000
H	49.29100000	14.23400000	37.09700000
N	50.30800000	14.60100000	38.94500000
C	51.62300000	15.01300000	39.14000000
C	52.36800000	15.38800000	40.29800000
O	52.04900000	15.28500000	41.49800000
N	53.68200000	15.78500000	40.07400000
H	54.29900000	15.90400000	40.86500000
C	54.22600000	15.81300000	38.85100000
N	55.46900000	16.17800000	38.75100000
H	55.97000000	16.50900000	39.56300000
H	55.92200000	16.13600000	37.84900000
N	53.55500000	15.48500000	37.73700000
C	52.29000000	15.05700000	37.90000000
C	53.94500000	9.60900000	38.56900000
H	53.71000000	10.41100000	37.86900000
O	53.67400000	9.99300000	39.88200000
P	53.27400000	11.47400000	40.35800000
O	52.91900000	11.47800000	41.81400000
O	52.38700000	12.08700000	39.36900000
O	54.63700000	12.30900000	40.22500000
C	55.66600000	12.11000000	41.12200000
H	55.61900000	11.07700000	41.46700000
H	55.35500000	12.81100000	41.89700000
Mg	50.31600000	12.67200000	41.07400000
O	50.16700000	10.78400000	38.65600000
H	50.98300000	11.28200000	38.60100000
H	50.05500000	10.41100000	37.78100000
O	49.46100000	10.51900000	42.83200000
H	48.89300000	9.94900000	43.35000000
H	49.82300000	11.13700000	43.46700000
O	47.61000000	12.42800000	40.90400000
H	46.98900000	12.95200000	40.39800000
H	47.68700000	11.60900000	40.41400000
O	49.22500000	14.98400000	42.62800000
H	49.48800000	15.83000000	42.26500000
H	48.27300000	15.04300000	42.70900000
H	56.64027442	12.32033913	40.73283835
H	54.98349252	9.38129068	38.44824506

H	50.73272814	14.39307893	34.90545694
H	52.05871643	13.41371398	35.51503544
H	53.49400471	8.67027677	38.32343545

1JID-991

C	32.20100000	35.23700000	64.95800000
H	32.33000000	34.18400000	64.70600000
N	32.99100000	35.97500000	63.95000000
C	33.04900000	37.31800000	63.61300000
H	32.35600000	38.05100000	64.00000000
N	34.08900000	37.70700000	62.89600000
C	34.74700000	36.50000000	62.76100000
C	35.98600000	36.21600000	62.05500000
O	36.68700000	36.91300000	61.41600000
N	36.29600000	34.87100000	62.00300000
H	37.15300000	34.63100000	61.52600000
C	35.48300000	33.87200000	62.41100000
N	35.99500000	32.66000000	62.34800000
H	36.99500000	32.69200000	62.21100000
H	35.38400000	31.94500000	62.71800000
N	34.33400000	34.06700000	63.05200000
C	34.04000000	35.42900000	63.20700000
C	36.87000000	35.54400000	68.50200000
H	36.02000000	35.27800000	67.87400000
O	37.85100000	36.25400000	67.70800000
P	37.83400000	35.93800000	66.17700000
O	39.08300000	36.50000000	65.64400000
O	36.48200000	36.36300000	65.68600000
O	37.98200000	34.32800000	65.97400000
C	39.17900000	33.76100000	66.34600000
H	39.38400000	33.89900000	67.40800000
H	39.88900000	34.22100000	65.65900000
C	35.73300000	46.14400000	63.66900000
H	34.98100000	45.45100000	64.04800000
C	37.14700000	45.51500000	63.74200000
O	38.21500000	46.10500000	63.87800000
N	37.13500000	44.23400000	63.52800000
H	36.22800000	43.80900000	63.40000000
C	38.34600000	43.50000000	63.24100000
H	39.03900000	43.42800000	64.08000000
C	38.03900000	41.96200000	62.83700000
H	37.26600000	42.05000000	62.07300000
H	38.92700000	41.45800000	62.45400000
C	37.56200000	41.14400000	64.04300000
H	38.48600000	40.89100000	64.56300000
H	36.90100000	41.73000000	64.68100000
C	36.88000000	39.88900000	63.60000000
H	35.95400000	40.18200000	63.10600000
H	37.50600000	39.27700000	62.94900000
N	36.31500000	39.10200000	64.76000000
H	36.75000000	38.21300000	64.95900000
C	35.31200000	39.43800000	65.57600000
N	34.86600000	40.65400000	65.67100000
H	35.08100000	41.37600000	64.99900000
H	34.15200000	40.85100000	66.35800000
N	34.84600000	38.52600000	66.33800000
H	35.20900000	37.58300000	66.32000000
H	34.08300000	38.78200000	66.94700000
C	39.21400000	44.21300000	62.13100000
O	40.45700000	44.42600000	62.34500000

N	38.50300000	44.66400000	61.07200000
H	37.52300000	44.42700000	61.01200000
C	39.06500000	45.45400000	60.02300000
H	39.85300000	44.79700000	59.65600000
H	39.59570598	46.31571333	60.37042093
H	38.34192597	45.66650381	59.26345667
H	35.45992742	46.38894199	62.66384589
H	35.63452616	46.99990798	64.30352694
H	31.15658964	35.46959794	64.96028037
H	32.62838395	35.39580179	65.92600049
H	36.62440764	36.06632611	69.40297715
H	37.30386604	34.66164323	68.92402700
H	39.25365904	32.71913648	66.11395164

1JID-NP-1145

C	18.38800000	34.35700000	36.35500000
H	18.07311122	35.18771437	36.95137127
N	19.86800000	34.46800000	36.07900000
C	20.63400000	33.52000000	35.52200000
H	20.37800000	32.48200000	35.37100000
N	21.72700000	34.01500000	35.00300000
C	21.67600000	35.40800000	35.28100000
C	22.43000000	36.53500000	34.82200000
O	23.37500000	36.45400000	34.03600000
N	21.96400000	37.79000000	35.24100000
H	22.35100000	38.56900000	34.72700000
C	20.85000000	37.94900000	36.03100000
N	20.36600000	39.14700000	36.10700000
H	20.91300000	39.91300000	35.74100000
H	19.57200000	39.37200000	36.69000000
N	20.05500000	36.90800000	36.38500000
C	20.53000000	35.68700000	35.98900000
C	17.30500000	36.25800000	31.02800000
H	17.34400000	35.83900000	32.03300000
O	18.59000000	36.57800000	30.48000000
P	19.91700000	36.78500000	31.40900000
O	21.16000000	36.83500000	30.49900000
O	19.99100000	35.78300000	32.45500000
O	19.62100000	38.18500000	32.01200000
C	19.80000000	39.36900000	31.20600000
H	19.66900000	39.15000000	30.14600000
H	20.86800000	39.55100000	31.33100000
Mg	22.87900000	35.22900000	31.85400000
O	23.57900000	37.71500000	31.13800000
H	23.63800000	38.62700000	31.42200000
H	22.68600000	37.62500000	30.80600000
O	20.56500000	33.12700000	32.30000000
H	20.46700000	33.93100000	32.81000000
H	19.69700000	32.72500000	32.31800000
O	23.43400000	35.29400000	28.73800000
H	23.20800000	34.45600000	29.14200000
H	22.59900000	35.64300000	28.42600000
O	22.88500000	32.78300000	30.35900000
H	22.89000000	31.86700000	30.08300000
H	22.19300000	32.83100000	31.01800000
O	24.05900000	33.59400000	33.46600000
H	23.50300000	33.34200000	34.20300000
H	24.27200000	32.76700000	33.03300000
O	25.60200000	34.89500000	31.48000000
H	25.94200000	35.01400000	32.36700000

H	25.43400000	33.95500000	31.41300000
H	16.93908521	35.51190473	30.35394643
H	16.56815857	37.01923541	30.87804882
H	19.17879352	40.15557886	31.58056136
H	18.19044233	33.44581737	36.87999255
H	17.85142796	34.35879188	35.42926395

Tables S1-S3 structures

1HQ1-483

N	45.02800000	24.74700000	22.11600000
C	44.79300000	26.03600000	21.81400000
H	45.48600000	26.60500000	21.21200000
N	43.61100000	26.50900000	22.11600000
C	42.99400000	25.42100000	22.73200000
C	41.70700000	25.21800000	23.31000000
O	40.85600000	26.08500000	23.60900000
N	41.45100000	23.93200000	23.69500000
H	40.55800000	23.74400000	24.12800000
C	42.43600000	23.01500000	23.73700000
N	42.12300000	21.87000000	24.30300000
H	41.19400000	21.78100000	24.68800000
H	42.87400000	21.19600000	24.25700000
N	43.72400000	23.24500000	23.34100000
C	43.88900000	24.35400000	22.76900000
O	40.98100000	22.70700000	18.40100000
P	40.54300000	23.26300000	19.84500000
O	39.35300000	24.11600000	19.70300000
O	41.76900000	23.80500000	20.51200000
O	40.09900000	21.99400000	20.70000000
C	46.19421397	23.88177739	21.88745063
H	46.59464594	23.56697747	22.82843695
H	45.89639952	23.02385786	21.32160556
H	46.94047295	24.42470924	21.34595037
C	42.13362496	21.88933453	18.18241616
H	41.82480171	20.91416044	17.86846761
H	42.74563609	22.33104309	17.42397307
H	42.69289036	21.81098072	19.09125217
C	38.89929096	21.23504428	20.52799870
H	39.03176238	20.25893542	20.94580585
H	38.09049810	21.72891639	21.02483313
H	38.67792840	21.14939134	19.48465687

1HQ1-825

N	52.92200000	21.93900000	26.34900000
C	52.54600000	22.95500000	25.45700000
H	53.29400000	23.63500000	25.07600000
N	51.24500000	23.11100000	25.26000000
C	50.67300000	22.10500000	25.97600000
C	49.37600000	21.65700000	26.07200000
O	48.31300000	22.12800000	25.63700000
N	49.18700000	20.51200000	26.87500000
H	48.27500000	20.09700000	27.00700000
C	50.21200000	19.90100000	27.50100000
N	49.96800000	18.81000000	28.13700000
H	48.99500000	18.57400000	28.27200000
H	50.66700000	18.38700000	28.73000000
N	51.47800000	20.37900000	27.47900000
C	51.67600000	21.45400000	26.68300000

O	50.08900000	18.56800000	22.35700000
P	49.72200000	19.63400000	23.47200000
O	48.82600000	20.65800000	22.84300000
O	51.00000000	20.03000000	24.11300000
O	48.90900000	18.71100000	24.51500000
C	54.25565221	21.50131870	26.78569995
H	54.31073826	21.53686322	27.85368971
H	54.42895720	20.49930885	26.45277107
H	54.99801445	22.14919985	26.36850895
C	51.01010193	17.50278008	22.60555131
H	50.51301719	16.56406354	22.47664044
H	51.82771486	17.57069892	21.91867715
H	51.37878959	17.57652547	23.60731527
C	47.68804651	18.04655573	24.17931206
H	47.70706908	17.04887039	24.56552718
H	46.86350795	18.57852258	24.60598221
H	47.57998232	18.01510283	23.11524775

1HQ1-1044

N	66.54000000	25.03900000	36.72800000
C	66.32900000	25.99800000	35.75600000
H	67.15100000	26.59900000	35.39600000
N	65.09100000	25.96200000	35.33400000
C	64.41300000	25.04500000	36.16800000
C	63.05900000	24.61600000	36.35600000
O	62.06300000	24.90600000	35.80300000
N	62.91100000	23.53800000	37.23400000
H	61.95000000	23.27300000	37.39200000
C	63.92600000	23.16500000	38.08000000
N	63.57400000	22.28900000	38.97600000
H	62.69100000	21.80300000	38.90300000
H	64.29100000	22.01700000	39.63300000
N	65.12400000	23.61300000	38.10600000
C	65.30700000	24.54800000	37.12000000
O	65.47600000	20.54200000	33.53100000
P	64.45900000	21.32700000	34.50500000
O	63.26600000	21.71800000	33.76100000
O	65.24200000	22.37400000	35.18300000
O	64.14300000	20.16800000	35.54900000
C	67.83795155	24.58506085	37.24777015
H	67.87421749	24.74453416	38.30519771
H	67.96058071	23.54264148	37.03986646
H	68.62382355	25.13758879	36.77658220
C	66.79320516	20.30609777	34.03520305
H	66.98844839	19.25410273	34.04450387
H	67.50828311	20.79603686	33.40788877
H	66.86848460	20.69163956	35.03048720
C	63.42282390	18.99191410	35.17075625
H	63.62245753	18.21002326	35.87338925
H	62.37401215	19.20357750	35.16113796
H	63.73312924	18.68213357	34.19472007

1HQ1-1206

N	61.06300000	22.42200000	33.55000000
C	60.94100000	23.27900000	32.49300000
H	61.83700000	23.72400000	32.08700000
N	59.77500000	23.18900000	31.86100000
C	59.05800000	22.26300000	32.60500000
C	57.85400000	21.61300000	32.26600000

O	57.00300000	21.87500000	31.38900000
N	57.47100000	20.62200000	33.16300000
H	56.49500000	20.38500000	33.26700000
C	58.21300000	20.31200000	34.23700000
N	57.61300000	19.42600000	34.98700000
H	56.65400000	19.14100000	34.85400000
H	58.03800000	19.05600000	35.82500000
N	59.44800000	20.82000000	34.56000000
C	59.83400000	21.83800000	33.66600000
O	60.89300000	17.96600000	30.27600000
P	59.62600000	18.81600000	30.81200000
O	58.84100000	19.26800000	29.63800000
O	60.29400000	19.78900000	31.62100000
O	58.73300000	17.78100000	31.65500000
C	62.19487470	22.13069429	34.44157201
H	61.90791487	22.31815291	35.45518600
H	62.47787755	21.10431600	34.33500285
H	63.02271294	22.75757524	34.18349427
C	61.85143231	17.33252972	31.12748278
H	61.73057278	16.27079355	31.07262582
H	62.83898496	17.59591825	30.81083434
H	61.70188750	17.65688234	32.13611142
C	57.76035774	16.94890709	31.01743555
H	57.43688539	16.19105484	31.70002298
H	56.92161097	17.54349819	30.72101477
H	58.19479594	16.48955353	30.15421034

1HQ1-1537

N	40.59800000	12.53400000	40.33000000
C	41.34300000	12.96000000	39.23100000
H	42.30400000	12.54800000	38.96000000
N	40.95100000	13.98600000	38.54600000
C	39.72100000	14.20800000	39.13400000
C	38.66500000	15.08700000	38.75200000
O	38.61100000	16.01300000	37.90200000
N	37.52200000	14.95200000	39.55100000
H	36.65900000	15.43000000	39.33700000
C	37.46100000	14.15000000	40.65700000
N	36.45500000	14.31300000	41.50600000
H	35.83300000	15.10600000	41.43700000
H	36.37600000	13.75800000	42.34500000
N	38.45000000	13.35900000	41.13700000
C	39.48700000	13.34400000	40.25900000
O	36.72000000	10.72700000	36.46300000
P	37.04500000	12.20200000	37.03900000
O	36.97000000	13.04000000	35.83400000
O	38.32600000	12.25400000	37.76600000
O	35.94200000	12.82900000	37.98900000
C	34.71741110	13.20595165	37.35402798
H	33.93657487	12.54284658	37.66303679
H	34.46522698	14.20796853	37.63199441
H	34.83413106	13.14909444	36.29193395
C	36.57731663	9.59287703	37.32224766
H	36.33519245	8.73034753	36.73716753
H	37.49568800	9.42328596	37.84448601
H	35.79430637	9.77638846	38.02802275
C	40.74916115	11.24152146	41.01377593
H	40.61423066	11.37704684	42.06654686
H	40.01542293	10.55525907	40.64557467
H	41.72785871	10.85147515	40.82692049

1HQ1-28

N	22.51200000	22.27100000	31.94400000
C	22.52700000	23.32200000	31.06900000
H	22.77900000	23.29500000	30.02000000
N	22.19700000	24.48200000	31.64400000
C	22.04900000	24.15900000	33.03300000
C	21.62600000	24.96000000	34.20000000
O	21.22000000	26.11300000	34.29900000
N	21.78400000	24.24000000	35.36500000
H	21.58000000	24.78100000	36.19300000
C	22.14400000	22.92800000	35.43800000
N	21.97500000	22.40300000	36.64700000
H	21.78800000	23.06100000	37.39000000
H	22.11700000	21.44500000	36.93400000
N	22.37200000	22.09400000	34.44600000
C	22.36200000	22.77200000	33.23900000
O	17.10200000	22.16300000	31.69300000
P	18.20500000	23.30000000	31.68500000
O	17.53000000	24.55000000	31.27600000
O	19.33000000	22.81200000	30.88100000
O	18.68500000	23.50800000	33.17900000
C	22.63657961	20.82491826	31.71113197
H	23.50247854	20.45509873	32.21941590
H	21.76521001	20.32743563	32.08278510
H	22.73273063	20.63963075	30.66169234
C	17.38431448	20.76140339	31.71993460
H	16.92553612	20.32164315	32.58076754
H	16.99580264	20.30224764	30.83498570
H	18.44284693	20.61157271	31.76420441
C	18.00310888	24.40067513	34.06390429
H	18.35799069	24.25320199	35.06250860
H	18.18944742	25.41095375	33.76471361
H	16.95166246	24.20581508	34.02662156

1HQ1-281

N	41.29300000	27.72800000	21.44100000
C	40.78400000	28.95300000	20.96600000
H	41.32600000	29.61200000	20.30400000
N	39.74800000	29.40200000	21.58800000
C	39.43700000	28.27800000	22.45000000
C	38.28000000	27.97100000	23.28700000
O	37.25200000	28.65500000	23.42100000
N	38.39900000	26.73500000	23.87600000
H	37.53100000	26.41200000	24.28000000
C	39.47800000	25.94900000	23.82000000
N	39.46300000	24.90700000	24.52100000
H	38.61800000	24.61700000	24.99200000
H	40.30300000	24.35700000	24.40500000
N	40.48800000	26.12100000	22.97400000
C	40.36400000	27.30600000	22.37000000
O	37.46900000	24.60900000	18.08900000
P	37.54400000	25.92600000	18.92800000
O	36.40100000	26.75400000	18.66600000
O	38.88100000	26.59500000	18.82800000
O	37.41300000	25.25600000	20.38800000
C	42.54412937	27.04948784	21.07330916
H	43.15429746	26.92997694	21.94412030
H	42.32040690	26.08826945	20.65989959

H	43.06836937	27.63633435	20.34826876
C	38.67189911	24.01428502	17.59478564
H	38.51847795	22.96501114	17.45200516
H	38.93601985	24.46635758	16.66163472
H	39.46127053	24.16649003	18.30092047
C	36.24648566	24.66386604	20.96551336
H	36.53081633	24.05321933	21.79687771
H	35.58334038	25.43442309	21.29925003
H	35.75245426	24.06089060	20.23253752

1HQ1-1008

N	67.19500000	29.10700000	33.49100000
C	66.42000000	29.75700000	32.60800000
H	66.83600000	30.60300000	32.08200000
N	65.31000000	29.08000000	32.41000000
C	65.39000000	27.94800000	33.26300000
C	64.42400000	26.85900000	33.48100000
O	63.31800000	26.72800000	33.02700000
N	64.87900000	25.90300000	34.28400000
H	64.26900000	25.10400000	34.38700000
C	66.09700000	25.97300000	34.91000000
N	66.30100000	25.13200000	35.83700000
H	65.69100000	24.32900000	35.88400000
H	67.14700000	25.24900000	36.37600000
N	66.92600000	27.02600000	34.84000000
C	66.55300000	27.96200000	33.93100000
O	69.01000000	23.59600000	29.23800000
P	67.53800000	23.69500000	29.88800000
O	66.88900000	22.93400000	28.81700000
O	67.16900000	25.09100000	30.11600000
O	67.50000000	22.82200000	31.28100000
C	70.02029435	24.60242719	29.34434677
H	70.97387711	24.17656036	29.11150057
H	69.80698683	25.39577240	28.65877891
H	70.03597329	24.98800941	30.34233501
C	67.23659213	21.41682032	31.24959368
H	67.32373236	21.01543635	32.23762075
H	66.24603512	21.24775433	30.88201578
H	67.94291362	20.93584256	30.60564465
C	68.61233033	29.40990959	33.73660237
H	68.78663087	29.47066512	34.79056066
H	69.22030293	28.63494350	33.31863480
H	68.86171896	30.34460529	33.27938333

1HQ1-1608

N	47.18200000	12.36700000	47.95500000
C	47.67700000	12.59800000	46.72300000
H	48.65200000	12.24400000	46.42300000
N	46.93100000	13.34400000	45.92800000
C	45.81100000	13.58500000	46.73800000
C	44.53700000	14.24500000	46.48700000
O	44.22800000	14.75600000	45.43400000
N	43.68500000	14.11400000	47.57000000
H	42.72800000	14.38900000	47.40400000
C	43.90500000	13.56500000	48.80300000
N	42.94700000	13.65400000	49.61700000
H	42.18700000	14.21200000	49.25400000
H	43.08600000	13.26400000	50.53800000
N	45.05300000	12.97300000	49.05000000

C	45.98000000	12.96000000	47.96300000
O	43.76400000	7.46700000	45.71700000
P	43.80500000	9.00600000	45.56100000
O	43.38200000	9.23400000	44.17900000
O	45.22100000	9.39100000	45.85900000
O	42.77600000	9.65300000	46.59600000
C	47.67509167	11.63082106	49.12794551
H	47.72438491	12.29099551	49.96856425
H	47.00912682	10.82267066	49.34768116
H	48.65068023	11.24293907	48.92136740
C	41.39751324	9.75316343	46.22906467
H	40.79718571	9.24768371	46.95638655
H	41.11374149	10.78397100	46.18652839
H	41.25015782	9.30278297	45.26971915
C	44.22765999	6.86283699	46.92733321
H	43.83795289	5.86912964	47.00199301
H	45.29704348	6.82704136	46.92119948
H	43.89491782	7.44027416	47.76444071

1HQ1-1706

N	44.45000000	11.49700000	45.93000000
C	45.67600000	11.89100000	45.42000000
H	46.58600000	11.60200000	45.92300000
N	45.50700000	12.55800000	44.31400000
C	44.13900000	12.70300000	44.16400000
C	43.39400000	13.20500000	43.05200000
O	43.81900000	13.50000000	41.91600000
N	42.01000000	13.08200000	43.24800000
H	41.41200000	13.55000000	42.58100000
C	41.42900000	12.48100000	44.32000000
N	40.15500000	12.56500000	44.47000000
H	39.56400000	13.06200000	43.81800000
H	39.71800000	12.12400000	45.26700000
N	42.17100000	11.98300000	45.31600000
C	43.49000000	12.12200000	45.22000000
O	40.31400000	7.15600000	41.65800000
P	40.90400000	8.22100000	40.69200000
O	40.72600000	7.80400000	39.27900000
O	42.21900000	8.64800000	41.17700000
O	40.03000000	9.58100000	40.87400000
C	38.73268526	9.66370132	40.27815191
H	37.98560594	9.49486143	41.02532363
H	38.59736212	10.63567768	39.85171362
H	38.64437010	8.92244621	39.51157426
C	44.19503371	10.41339326	46.89004615
H	43.53291989	10.76268690	47.65457166
H	43.74767419	9.58472332	46.38203474
H	45.11891946	10.10402212	47.33234088
C	41.13828965	6.75634441	42.75605373
H	40.63332488	6.00464358	43.32601552
H	42.06108558	6.36234998	42.38441405
H	41.33723439	7.60299686	43.37935191

1HQ1-919

N	56.42900000	18.59300000	31.74400000
C	55.32200000	18.21900000	32.50500000
H	54.37400000	17.81100000	32.18800000
N	55.63600000	18.20000000	33.76300000
C	56.97000000	18.67700000	33.86200000

C	57.94600000	18.83100000	34.92600000
O	57.75500000	18.74000000	36.14700000
N	59.21000000	19.19100000	34.55200000
H	59.87700000	19.60300000	35.19000000
C	59.55900000	19.26800000	33.25700000
N	60.79800000	19.71600000	33.02500000
H	61.41400000	19.88800000	33.80600000
H	61.00800000	20.03100000	32.08800000
N	58.75600000	19.09800000	32.18100000
C	57.46800000	18.89100000	32.59200000
O	59.79800000	14.10600000	33.90000000
P	59.41300000	15.52200000	34.32200000
O	58.98500000	15.41700000	35.73100000
O	58.50400000	16.14000000	33.33700000
O	60.79300000	16.36400000	34.40400000
C	61.84028756	16.09501892	35.33980871
H	62.70070367	15.73193352	34.81755100
H	62.09274518	16.99480064	35.86092099
H	61.51104822	15.35705708	36.04117437
C	60.12602535	13.80260118	32.54160075
H	60.65801645	12.87507850	32.50176193
H	59.22703382	13.72311830	31.96679929
H	60.73847122	14.58258819	32.13981683
C	56.60312430	18.45746006	30.29065570
H	57.00205537	19.36713742	29.89286039
H	57.27767221	17.65252892	30.08578750
H	55.65638876	18.25405556	29.83544319

1HQ1-968

N	40.34600000	20.45500000	27.94400000
C	39.47100000	20.21200000	28.94500000
H	38.40900000	20.37100000	28.82700000
N	40.07900000	19.76700000	30.03200000
C	41.45500000	19.67400000	29.69100000
C	42.61300000	19.26800000	30.41000000
O	42.65000000	18.76200000	31.52900000
N	43.74500000	19.25100000	29.63800000
H	44.59000000	18.97800000	30.11900000
C	43.79700000	19.69600000	28.37900000
N	44.97800000	19.85800000	27.87100000
H	45.76800000	19.53400000	28.41000000
H	45.18200000	20.43500000	27.06700000
N	42.80300000	20.24200000	27.73700000
C	41.61900000	20.12400000	28.40400000
O	42.13900000	14.80900000	27.63600000
P	42.67900000	16.00100000	28.48900000
O	42.36600000	15.63200000	29.86300000
O	42.10500000	17.20200000	27.81200000
O	44.28000000	15.99800000	28.36300000
C	40.03002463	20.58279896	26.51406049
H	40.59753774	21.38669436	26.09384502
H	40.27685297	19.67105233	26.01137386
H	38.98568754	20.78367388	26.39612273
C	42.09128476	14.94910008	26.21367963
H	42.69207845	14.18873562	25.76003569
H	41.07958779	14.85108028	25.87937233
H	42.46648504	15.91231446	25.93737763
C	45.05578953	15.02439959	29.06667098
H	46.06075422	15.03594690	28.69950258
H	45.05512014	15.25515028	30.11149330

H	44.63197983	14.05360367	28.91553987

1HQ1-1418

N	47.80600000	43.01800000	15.67900000
C	46.94500000	44.06000000	16.02200000
H	47.22000000	45.08700000	15.83000000
N	45.75300000	43.66600000	16.29900000
C	45.84900000	42.30300000	16.37700000
C	44.88000000	41.35300000	16.78200000
O	43.74400000	41.54500000	17.25700000
N	45.33300000	40.10100000	16.78500000
H	44.71500000	39.38900000	17.14500000
C	46.57500000	39.77800000	16.53400000
N	47.00000000	38.57800000	16.56300000
H	46.41700000	37.84500000	16.93900000
H	47.90300000	38.38300000	16.15600000
N	47.56600000	40.57900000	16.06500000
C	47.08400000	41.86200000	16.01700000
O	44.71800000	40.11400000	11.62100000
P	44.34800000	40.33100000	13.23400000
O	42.91400000	40.73200000	13.22700000
O	45.35100000	41.29700000	13.72300000
O	44.59600000	38.84800000	13.68400000
C	49.13064095	43.14770655	15.05497871
H	49.87610185	42.75781575	15.71616946
H	49.14719556	42.59981697	14.13604294
H	49.33281946	44.17989921	14.85850433
C	45.90374885	40.57299955	10.96661550
H	46.00830701	40.07337580	10.02622000
H	45.83567920	41.62839990	10.80414512
H	46.75449865	40.36067002	11.57983728
C	43.71554271	37.77952313	13.32615628
H	44.15637940	36.84539675	13.60538901
H	42.78259733	37.90019753	13.83600979
H	43.54884797	37.79348577	12.26931291

1HQ1-1955

N	61.10200000	59.59500000	26.23600000
C	59.97800000	60.38800000	26.13900000
H	60.07000000	61.38900000	26.53200000
N	58.93500000	59.84700000	25.57200000
C	59.43500000	58.54000000	25.25900000
C	58.88400000	57.41600000	24.54200000
O	57.72000000	57.23500000	24.05400000
N	59.74100000	56.33000000	24.46200000
H	59.47800000	55.49400000	23.95800000
C	61.05500000	56.33800000	24.74800000
N	61.66900000	55.20500000	24.61100000
H	61.04200000	54.46700000	24.32400000
H	62.66700000	55.23300000	24.76100000
N	61.61900000	57.39200000	25.32200000
C	60.78100000	58.43300000	25.63100000
O	60.94700000	59.30300000	20.17900000
P	60.16900000	58.43700000	21.20900000
O	58.80700000	58.60800000	20.74700000
O	60.44300000	58.89100000	22.55300000
O	60.69900000	56.97400000	20.84400000
C	62.47619802	59.92492228	26.64051340
H	62.83075019	59.18941416	27.33204834

H	63.10874758	59.93738782	25.77759645
H	62.48936244	60.88811231	27.10633705
C	62.35753468	59.53208684	20.23202010
H	62.82169721	59.09997701	19.37019979
H	62.54747830	60.58495184	20.24924562
H	62.75886313	59.08274620	21.11628729
C	60.15195107	56.09584251	19.85684404
H	60.79572260	55.24974750	19.73609034
H	59.18379572	55.76547514	20.17058377
H	60.06700475	56.61522232	18.92521711

1HQ1-2648

N	73.26400000	43.42300000	33.79300000
C	72.74900000	44.40900000	33.00500000
H	73.10800000	45.42800000	33.00300000
N	71.77700000	44.07400000	32.22900000
C	71.54100000	42.70600000	32.54200000
C	70.69700000	41.70400000	31.94700000
O	69.87200000	41.86300000	31.01800000
N	70.92300000	40.43600000	32.54200000
H	70.25900000	39.71800000	32.29400000
C	71.85700000	40.16300000	33.47200000
N	71.76100000	38.98200000	33.97900000
H	71.03500000	38.38800000	33.60400000
H	72.13600000	38.68800000	34.86900000
N	72.61000000	41.07400000	34.07600000
C	72.41500000	42.34900000	33.54500000
O	75.29200000	40.32200000	29.56300000
P	73.72800000	40.31700000	29.95700000
O	72.93400000	40.52600000	28.71800000
O	73.64200000	41.29100000	30.94800000
O	73.47300000	38.86600000	30.58100000
C	74.50816267	43.34314487	34.57183400
H	74.28847304	42.99821248	35.56059996
H	75.18256556	42.66100378	34.09774089
H	74.95906404	44.31209256	34.62406730
C	76.37230980	40.19305325	30.49101049
H	76.84577933	39.24285506	30.35740654
H	77.08420487	40.97341715	30.32031311
H	75.99528854	40.26640295	31.48969702
C	73.07384911	37.79651936	29.71972784
H	73.11995216	36.87124970	30.25512542
H	72.07207542	37.96440909	29.38333147
H	73.73085454	37.75365888	28.87627848

1HQ1-1292

N	45.96400000	51.64600000	16.61200000
C	44.65800000	51.86600000	17.06600000
H	44.26700000	52.83000000	17.35600000
N	43.98700000	50.72700000	17.24100000
C	44.84200000	49.72200000	16.92000000
C	44.68100000	48.26300000	16.91600000
O	43.74200000	47.55600000	17.20500000
N	45.82600000	47.64100000	16.50200000
H	45.84100000	46.63100000	16.48200000
C	46.92600000	48.27100000	16.07000000
N	48.01500000	47.52400000	15.86200000
H	47.98000000	46.53600000	16.06800000
H	48.90400000	47.91900000	15.59000000

N	47.05800000	49.56200000	16.00000000
C	46.03800000	50.23300000	16.51200000
O	45.42000000	49.50200000	11.91700000
P	44.50200000	48.24600000	11.59000000
O	43.76800000	48.51400000	10.33000000
O	43.74800000	47.94500000	12.83900000
O	45.60500000	47.12200000	11.29700000
C	46.24010426	46.45798205	12.39272932
H	45.99115385	45.41755248	12.37199284
H	47.30094263	46.57254653	12.31274860
H	45.90343394	46.88699479	13.31332759
C	46.31212714	50.22762415	11.06701250
H	45.79237389	51.05196211	10.62517149
H	47.13594433	50.59319832	11.64371067
H	46.67559864	49.58066130	10.29615095
C	47.09948068	52.50017715	16.23521753
H	47.95074658	52.24529659	16.83127313
H	47.33047268	52.35080080	15.20118204
H	46.84372876	53.52618183	16.39894093

1HQ1-1591

N	60.11700000	38.49400000	19.91100000
C	59.43700000	39.48300000	19.20100000
H	59.95600000	40.19000000	18.57000000
N	58.10000000	39.44900000	19.38400000
C	57.93400000	38.39800000	20.31100000
C	56.75700000	37.77900000	20.78700000
O	55.56300000	37.99600000	20.59300000
N	56.97100000	36.71900000	21.60900000
H	56.15300000	36.24400000	21.96300000
C	58.15600000	36.17800000	21.93400000
N	58.28800000	35.18300000	22.74000000
H	57.44500000	34.69600000	23.00900000
H	59.16800000	35.03700000	23.21300000
N	59.30100000	36.75500000	21.50400000
C	59.13800000	37.82100000	20.66600000
O	57.90100000	33.39000000	15.89500000
P	56.70600000	34.51900000	16.06100000
O	55.39600000	33.83200000	16.12900000
O	56.88700000	35.50600000	15.08300000
O	57.05600000	35.12500000	17.48600000
C	59.26777002	33.79686198	15.78868431
H	59.79821940	33.49846436	16.66873108
H	59.71152021	33.33568153	14.93118890
H	59.31625808	34.86087516	15.68658207
C	61.47363871	38.01668300	19.60669769
H	62.01225849	37.86743391	20.51911944
H	61.41473860	37.09178518	19.07191288
H	61.98140395	38.74339528	19.00756188
C	56.62062338	34.56234391	18.72646980
H	57.17830073	34.99604888	19.53008412
H	55.57924837	34.76582722	18.86442856
H	56.77854989	33.50414736	18.71308002

1HQ1-2366

N	71.98200000	56.64600000	42.26200000
C	71.21100000	57.30200000	41.32100000
H	71.14600000	58.37800000	41.25300000
N	70.56800000	56.55000000	40.42600000

C	70.76300000	55.30400000	40.94500000
C	70.51800000	53.99300000	40.29700000
O	69.79300000	53.74100000	39.33800000
N	71.01700000	52.91700000	41.01000000
H	70.92300000	52.02800000	40.54100000
C	71.67600000	53.04100000	42.13300000
N	71.93000000	51.95200000	42.77200000
H	71.35900000	51.23100000	42.35400000
H	72.43400000	51.91900000	43.64600000
N	72.06300000	54.16800000	42.67800000
C	71.61500000	55.30400000	42.02400000
O	75.37600000	55.46000000	37.75100000
P	73.98900000	54.82900000	37.97600000
O	73.59500000	54.21600000	36.74100000
O	73.14900000	55.84100000	38.62600000
O	74.20500000	53.60600000	38.92100000
C	74.66437370	52.38002191	38.34580131
H	75.53310543	52.04280965	38.87162740
H	73.89399553	51.64105200	38.41888315
H	74.90974716	52.53886424	37.31650010
C	76.18489659	55.87241772	38.85576148
H	76.79564896	55.05417496	39.17569949
H	76.80950953	56.68773295	38.55572853
H	75.55478929	56.18393742	39.66249580
C	73.00644368	57.13729449	43.19476195
H	72.75611272	56.83600667	44.19048283
H	73.95821376	56.72822993	42.92699358
H	73.05068774	58.20525568	43.14575833

1HQ1-3633

N	31.64400000	44.95200000	18.85200000
C	32.24600000	46.22100000	18.91500000
H	32.96300000	46.42400000	18.13200000
N	31.69000000	46.97200000	19.85100000
C	30.67100000	46.17400000	20.34500000
C	29.49800000	46.55600000	21.10000000
O	29.14600000	47.69000000	21.46100000
N	28.78700000	45.41400000	21.46200000
H	27.98600000	45.59400000	22.05000000
C	28.93400000	44.17100000	20.95100000
N	28.09800000	43.18400000	21.16600000
H	27.47800000	43.31000000	21.95400000
H	28.46400000	42.26600000	20.95600000
N	29.89200000	43.81000000	20.09500000
C	30.75900000	44.89500000	19.86200000
O	27.05000000	47.54200000	16.41700000
P	27.64300000	47.64400000	17.84000000
O	27.43600000	49.05500000	18.39100000
O	29.00800000	47.00000000	17.79400000
O	26.68100000	46.63700000	18.63500000
C	31.68700000	44.00400000	17.73000000
H	31.84400000	43.01400000	18.10500000
H	30.76000000	44.04000000	17.19700000
H	32.48800000	44.26700000	17.07100000
C	27.33700000	46.49100000	15.49100000
H	26.42100000	46.07300000	15.13000000
H	27.89900000	46.88300000	14.67000000
H	27.90700000	45.72900000	15.98200000
C	25.40700000	47.00100000	19.17400000
H	24.86700000	46.11600000	19.44100000

H	25.54900000	47.60800000	20.04400000
H	24.85300000	47.54900000	18.44200000

1HQ1-3833

N	43.86200000	56.13200000	17.11100000
C	45.16900000	56.53400000	17.14200000
H	45.86800000	56.44600000	16.32400000
N	45.50600000	57.20000000	18.21700000
C	44.30200000	57.26100000	18.94900000
C	44.00300000	57.88400000	20.20000000
O	44.73300000	58.67600000	20.79000000
N	42.73200000	57.65300000	20.61900000
H	42.47200000	58.17200000	21.44500000
C	41.82100000	56.98100000	19.86200000
N	40.65700000	56.71700000	20.44800000
H	40.44100000	57.16000000	21.33000000
H	39.95400000	56.14300000	20.00600000
N	42.06500000	56.34700000	18.71300000
C	43.37000000	56.53300000	18.30100000
O	41.35500000	62.18300000	17.43800000
P	41.42200000	62.78100000	18.86800000
O	41.43500000	64.24900000	18.72400000
O	42.56400000	62.19200000	19.64000000
O	40.04900000	62.35700000	19.63200000
C	39.86989845	61.07475101	20.23917392
H	39.74457055	61.19447029	21.29504333
H	39.00160315	60.60361062	19.82808999
H	40.72950871	60.46672748	20.04870719
C	43.04952759	55.61021913	16.00260778
H	42.54984030	54.71750968	16.31610810
H	42.32437581	56.34302353	15.71615952
H	43.68297547	55.39032467	15.16876714
C	40.18862988	62.11146263	16.61376511
H	40.47771644	62.13514690	15.58382919
H	39.66416085	61.20104056	16.81616049
H	39.55127421	62.94467254	16.82457075

1JID-38

N	31.70200000	36.04800000	62.45800000
C	30.68300000	36.69300000	61.81300000
H	29.62400000	36.53100000	61.95300000
N	31.06000000	37.74200000	61.11600000
C	32.46200000	37.77600000	61.25900000
C	33.44900000	38.71900000	60.88200000
O	33.32700000	39.71900000	60.19400000
N	34.72000000	38.47600000	61.37100000
H	35.52200000	39.05200000	61.16200000
C	34.99900000	37.36300000	62.04700000
N	36.23400000	37.09500000	62.24400000
H	36.91300000	37.75700000	61.89600000
H	36.49000000	36.19500000	62.62600000
N	34.16700000	36.42500000	62.43400000
C	32.87000000	36.71100000	62.04900000
C	33.01200000	38.87000000	67.19300000
H	32.75100000	37.96400000	66.64700000
O	32.76100000	40.05600000	66.45100000
P	33.21200000	40.26000000	64.96100000
O	32.53300000	41.42000000	64.46300000
O	33.12600000	38.95500000	64.25100000

O	34.75200000	40.67800000	65.12200000
C	35.75600000	40.18000000	64.16800000
H	35.46400000	40.44600000	63.15200000
H	35.78800000	39.09100000	64.17200000
H	32.45247277	38.75290596	68.09749890
H	34.03115697	38.87993607	67.51876118
H	36.71875159	40.52175598	64.48613869
C	31.63515926	34.95790626	63.44193493
H	32.23697557	34.13863158	63.10800958
H	31.99928243	35.30651382	64.38575010
H	30.62056698	34.63510410	63.54824234

1JID-38 (Optimized)

N	-1.5478426	-0.3012785	-2.8265121
C	-1.7169772	1.0590233	-3.0728853
H	-2.6348387	1.4353583	-3.5464146
N	-0.6918473	1.7910483	-2.6831295
C	0.1958543	0.8867595	-2.1358541
C	1.5020516	1.0855472	-1.5419134
O	2.1840668	2.0925555	-1.3954617
N	2.0229809	-0.1931826	-1.1167308
H	2.8998261	-0.1103587	-0.5957872
C	1.3726659	-1.4024401	-1.1606290
N	2.0247381	-2.4881563	-0.5832802
H	2.4509428	-2.2574086	0.3215721
H	1.3605619	-3.2592417	-0.4668301
N	0.2268051	-1.5885283	-1.7864613
C	-0.3146005	-0.4248932	-2.2175680
C	-2.2792353	-1.1409206	3.2041521
H	-2.3341907	-1.4318372	2.1298982
O	-1.6477965	0.1218419	3.3680943
P	-0.3982901	0.5643946	2.3341161
O	-0.2934572	2.0654795	2.3809306
O	-0.4440088	-0.2709850	1.0477634
O	0.8119228	-0.1326610	3.3210257
C	1.9404119	-0.7062040	2.5702284
H	2.4982454	0.0720495	1.9960423
H	1.5712860	-1.4743067	1.8528743
H	-3.3068234	-1.0854729	3.6297629
H	-1.7269335	-1.9546342	3.7411198
H	2.6303314	-1.1669223	3.3102550
C	-2.4801244	-1.3855639	-3.0511550
H	-1.9981969	-2.3162241	-2.6951066
H	-3.4180442	-1.2309513	-2.4791443
H	-2.7250427	-1.4913155	-4.1290548

1JID-492

C	28.03700000	27.16600000	60.47100000
H	28.30400000	26.49700000	59.68000000
N	28.92500000	28.27900000	60.47100000
C	28.72800000	29.57500000	60.81000000
H	27.80700000	30.00700000	61.17100000
N	29.83200000	30.32300000	60.75100000
C	30.78800000	29.42000000	60.36900000
C	32.23800000	29.54100000	60.28200000
O	32.90700000	30.52400000	60.59000000
N	32.87900000	28.41800000	59.81600000
H	33.88300000	28.31300000	59.79200000
C	32.20200000	27.28500000	59.50100000

N	32.83500000	26.32600000	58.80200000
H	33.83300000	26.37200000	58.65000000
H	32.26500000	25.52200000	58.58400000
N	30.86600000	27.16500000	59.60900000
C	30.21200000	28.23700000	60.13700000
C	31.79400000	25.34000000	64.82600000
H	31.09700000	25.34800000	63.98800000
O	32.78200000	26.36700000	64.83500000
P	33.30100000	27.01200000	63.42400000
O	34.41700000	27.97100000	63.85100000
O	32.08000000	27.62000000	62.79000000
O	33.96100000	25.94900000	62.47800000
C	35.20300000	25.29300000	62.90200000
H	35.13900000	24.85400000	63.89800000
H	35.99600000	26.04000000	62.90200000
H	32.28700000	24.39300000	64.75600000
H	31.21700000	25.17400000	65.71200000
H	35.34900000	24.45100000	62.25800000
H	27.03400000	27.51000000	60.32400000
H	28.10500000	26.65500000	61.40900000

1JID-492 (Optimized)

C	-2.3021127	0.8552703	2.8240613
H	-1.8004707	1.8336773	2.6937249
N	-1.2939937	-0.1786572	2.7543861
C	-1.4030448	-1.5285499	3.0960308
H	-2.3045970	-1.9097369	3.5966127
N	-0.3393718	-2.2383012	2.7686972
C	0.5007740	-1.3289919	2.1580382
C	1.7653357	-1.4984094	1.4766137
O	2.4325810	-2.5383531	1.4002740
N	2.2457742	-0.3640117	0.8671229
H	3.0947853	-0.4928810	0.3133554
C	1.6530313	0.8451414	1.0339769
N	2.3139542	1.9493647	0.6430349
H	2.8059217	1.8653127	-0.2542692
H	1.6787646	2.7519877	0.6322244
N	0.5007871	1.0642530	1.6537418
C	-0.0633687	-0.0563691	2.1453916
C	-2.4530535	0.0264039	-2.8098133
H	-2.6381034	0.1702771	-1.7186524
O	-1.4663962	-0.9762485	-3.0381571
P	-0.0336813	-1.0560966	-2.1216291
O	0.7364755	-2.2094825	-2.7197241
O	-0.3904617	-0.8311363	-0.6626568
O	0.7671355	0.3951304	-2.5373157
C	1.2578007	0.6324710	-3.8995143
H	0.4274692	0.9176929	-4.5876476
H	1.7433477	-0.2864438	-4.2992489
H	-2.1548735	1.0191677	-3.2321518
H	-3.3992163	-0.2928672	-3.3013674
H	1.9935445	1.4645228	-3.8558849
H	-2.8200015	0.8356281	3.8048672
H	-3.0529827	0.7407013	2.0133080

1JID-966

N	31.59200000	36.67500000	63.36400000
C	31.54300000	38.04800000	63.11900000
H	30.65500000	38.64400000	63.26600000

N	32.57400000	38.45000000	62.44500000
C	33.50500000	37.36200000	62.47000000
C	34.87700000	37.18600000	62.15100000
O	35.69200000	38.01900000	61.76000000
N	35.29900000	35.91600000	62.29900000
H	36.28700000	35.71300000	62.24600000
C	34.56900000	34.91600000	62.63600000
N	35.08600000	33.67700000	62.57000000
H	35.94300000	33.56400000	62.04700000
H	34.56600000	32.83400000	62.76800000
N	33.22900000	34.94500000	62.89700000
C	32.80500000	36.25500000	62.92600000
O	35.92900000	37.16400000	67.47000000
P	36.24100000	36.87000000	65.89700000
O	37.38500000	37.67400000	65.56000000
O	34.97800000	37.18700000	65.16800000
O	36.58700000	35.33600000	65.71800000
C	37.88367256	34.83850930	66.05865126
H	37.78864781	34.07428268	66.80150252
H	38.35015425	34.43154827	65.18590986
H	38.48245271	35.63744884	66.44343422
C	34.98493498	36.36405966	68.18675428
H	35.46459726	35.47388848	68.53663907
H	34.60880303	36.91811887	69.02128598
H	34.17500663	36.10161513	67.53865018
C	30.64276218	35.86035560	64.13614121
H	30.33592749	35.01912214	63.55041197
H	31.11449202	35.51735362	65.03320342
H	29.78692547	36.45161887	64.38684278

1JID-966 (Optimized)

N	-3.1531456	-0.4829543	1.2036219
C	-3.3805035	0.8519753	1.5484002
H	-4.0115756	1.1100997	2.4106838
N	-2.7783200	1.7088182	0.7452214
C	-2.1076272	0.9140406	-0.1604138
C	-1.2147501	1.2239999	-1.2585245
O	-0.9095958	2.3427772	-1.6893584
N	-0.7447235	0.1319209	-1.8904472
H	-0.0553331	0.3630183	-2.6132473
C	-1.0122369	-1.0856462	-1.5864146
N	-0.6170754	-2.0684551	-2.4138807
H	0.3499710	-1.9889172	-2.7514928
H	-0.7720392	-2.9807989	-1.9774516
N	-1.8401858	-1.4834681	-0.6076157
C	-2.3272347	-0.4397839	0.0975859
O	3.1359160	0.1727486	1.8017901
P	2.2505553	0.5196857	0.4077564
O	2.9719058	1.6709915	-0.2530267
O	0.7689705	0.4129674	0.7254413
O	2.5303929	-0.8932251	-0.5092907
C	3.7410680	-1.0695387	-1.2496146
H	4.5326196	-1.5813449	-0.6469098
H	3.5322896	-1.7060906	-2.1418075
H	4.1449432	-0.0866965	-1.5852670
C	2.9558586	-1.0156389	2.5765253
H	3.2582574	-1.9316530	2.0107864
H	3.5881657	-0.9339538	3.4873772
H	1.8900457	-1.1502881	2.8800830
C	-3.5751487	-1.6806216	1.8939483

H	-4.6604441	-1.6456702	2.1229472
H	-3.3696019	-2.5440173	1.2319900
H	-3.0125939	-1.8251310	2.8413009

1JID-1438

N	23.92800000	43.56200000	57.36600000
C	24.97700000	43.99600000	58.10500000
H	25.16400000	45.02900000	58.35800000
N	25.84800000	43.03800000	58.49000000
C	25.25000000	41.90600000	57.94600000
C	25.61800000	40.48300000	57.99900000
O	26.64700000	40.06200000	58.51200000
N	24.66600000	39.64900000	57.51000000
H	25.04300000	38.71700000	57.41600000
C	23.51600000	40.05400000	56.88900000
N	22.81700000	39.11000000	56.28500000
H	23.12300000	38.14800000	56.27200000
H	22.22200000	39.43700000	55.53800000
N	23.16900000	41.34900000	56.69400000
C	24.05400000	42.23500000	57.29900000
O	20.93900000	40.61800000	61.69100000
P	22.04200000	39.75000000	60.87900000
O	22.93200000	39.34300000	61.89300000
O	22.53500000	40.57100000	59.74500000
O	21.19200000	38.54500000	60.27100000
C	22.71737822	44.26475118	56.91718412
H	22.56491197	44.08193171	55.87400097
H	21.87277936	43.90822187	57.46894459
H	22.83324284	45.31562614	57.08191785
C	19.75956193	41.12275951	61.05930097
H	19.01896778	40.35155927	61.01842928
H	19.38107331	41.95133443	61.62064753
H	19.99612849	41.44307187	60.06615634
C	21.54429687	37.91025822	59.03897330
H	20.99093621	37.00031375	58.93556617
H	21.31359396	38.56269910	58.22288687
H	22.59196713	37.69281537	59.03660112

1JID-1438 (Optimized)

N	0.5196156	-1.7122987	-2.6272210
C	-0.7774136	-1.9312833	-3.0834754
H	-1.0849170	-2.9256518	-3.4384871
N	-1.5368441	-0.8556021	-3.0526123
C	-0.7225453	0.1224198	-2.5129431
C	-1.0531937	1.4345283	-1.9944916
O	-2.0807208	2.0992678	-2.0677068
N	0.0823003	1.9611646	-1.2722274
H	-0.1310997	2.8332546	-0.7807558
C	1.3187153	1.3778273	-1.1326103
N	2.3010258	2.1171647	-0.4856320
H	1.9526963	2.6608520	0.3087416
H	3.0589973	1.4960168	-0.1870155
N	1.6357520	0.2164251	-1.6757843
C	0.5644679	-0.3892569	-2.2417285
O	-0.6514838	-1.5369603	3.3574707
P	-0.6313100	-0.2064537	2.3270401
O	-2.0267858	0.3602313	2.3026959
O	0.2081864	-0.5131511	1.0815060
O	0.3673048	0.7736288	3.3268206

C	1.5282285	-2.6956859	-2.2877566
H	2.5237156	-2.3582435	-2.6371956
H	1.5702061	-2.8253014	-1.1862086
H	1.2772962	-3.6610245	-2.7681505
C	0.5685449	-2.2666233	3.5124138
H	1.2891655	-1.7242806	4.1711524
H	0.3286907	-3.2489634	3.9764613
H	1.0648878	-2.4304438	2.5284608
C	1.1032722	1.8055374	2.6647015
H	1.5994388	2.4310626	3.4405368
H	1.8782223	1.3777060	1.9874038
H	0.4421148	2.4753520	2.0547763

1JID-1877

N	35.57100000	43.01200000	62.00800000
C	36.89000000	42.52400000	61.80000000
H	37.77500000	43.14100000	61.85300000
N	36.94400000	41.25500000	61.67600000
C	35.59800000	40.85100000	61.78400000
C	35.02200000	39.56000000	61.92900000
O	35.53700000	38.43000000	62.02400000
N	33.63900000	39.57800000	62.08800000
H	33.16100000	38.69000000	62.12900000
C	32.88800000	40.71500000	62.08300000
N	31.59100000	40.46900000	61.93200000
H	31.37200000	39.56700000	61.53200000
H	30.96700000	41.25700000	61.83700000
N	33.36000000	41.91100000	62.05100000
C	34.76200000	41.91300000	61.96900000
O	35.25500000	40.58200000	67.18000000
P	34.68700000	39.77800000	65.93600000
O	35.56300000	38.60200000	65.85400000
O	34.62900000	40.76700000	64.77000000
O	33.26700000	39.16300000	66.34200000
C	32.37732937	38.58805678	65.38136143
H	32.93178425	37.96881088	64.70755560
H	31.64160606	37.99696789	65.88556884
H	31.89290047	39.36818954	64.83216063
C	35.07648427	44.31562983	62.47372895
H	35.88279694	45.01889399	62.48712235
H	34.31067838	44.66358255	61.81238921
H	34.67602398	44.21331316	63.46067518
C	34.61141396	41.79479295	67.57978779
H	35.08795493	42.17891126	68.45743341
H	34.68397502	42.51392314	66.79080956
H	33.58074773	41.59901848	67.79026221

1JID-1877 (Optimized)

N	-0.4624630	1.5124945	-2.7249150
C	0.8773559	1.8772413	-2.8347434
H	1.1555097	2.8862541	-3.1727746
N	1.7139351	0.9051780	-2.5396965
C	0.9000072	-0.1449430	-2.1620281
C	1.2373650	-1.3771437	-1.4764862
O	2.3155705	-1.9181550	-1.2693798
N	0.0240330	-1.9962529	-0.9869576
H	0.2064112	-2.7718314	-0.3450823
C	-1.2585716	-1.5344134	-1.1627636
N	-2.2693231	-2.2767852	-0.5876017

H	-2.0234324	-2.6634445	0.3304004
H	-3.1207721	-1.7120001	-0.5180178
N	-1.5679384	-0.4815486	-1.9009328
C	-0.4596829	0.2130615	-2.2624666
O	0.3721449	1.7885804	3.0830927
P	0.4601468	0.3666492	2.1698358
O	1.9038606	-0.0638710	2.1603451
O	-0.4215339	0.4982295	0.9230483
O	-0.4157402	-0.6653582	3.2422578
C	-0.7708729	-1.9638711	2.7599366
H	0.1190308	-2.5311502	2.3893261
H	-1.2342828	-2.5361641	3.5934701
H	-1.5093287	-1.8922898	1.9207213
C	-1.6256711	2.3738952	-2.7489241
H	-1.4365025	3.2423880	-3.4098044
H	-2.4964388	1.8053019	-3.1284258
H	-1.8671654	2.7311895	-1.7255622
C	-0.8650444	2.5046957	3.1209041
H	-0.6465421	3.5661540	3.3745121
H	-1.3828239	2.4559874	2.1358879
H	-1.5575072	2.0924356	3.8951827

1JID-96

N	26.66300000	32.54800000	62.48300000
C	25.74700000	33.28700000	61.80000000
H	24.75100000	32.89700000	61.64900000
N	26.24900000	34.27100000	61.22800000
C	27.66100000	34.22900000	61.53600000
C	28.70500000	35.13400000	61.25800000
O	28.70400000	36.22400000	60.64400000
N	29.84400000	34.73200000	61.79600000
H	30.56600000	35.43800000	61.83500000
C	30.00900000	33.51400000	62.42100000
N	31.21000000	33.33200000	62.82400000
H	31.83400000	34.12400000	62.77500000
H	31.36800000	32.53900000	63.42900000
N	29.06100000	32.70300000	62.79700000
C	27.87400000	33.12100000	62.26600000
C	26.17300000	34.87400000	68.18000000
H	26.07100000	34.13900000	67.38200000
O	26.19900000	36.22400000	67.69800000
P	26.84300000	36.42800000	66.29200000
O	26.47600000	37.76600000	65.82000000
O	26.47800000	35.28800000	65.47200000
O	28.35300000	36.38200000	66.67700000
C	29.30300000	36.45300000	65.60400000
H	29.26900000	37.42800000	65.11900000
H	29.00700000	35.60500000	64.98600000
H	26.97891676	34.68957173	68.85925281
H	30.24408744	36.35011143	66.10266660
H	25.34621327	34.64252632	68.81854808
C	26.41907424	31.31558478	63.24625156
H	26.96645468	30.50878999	62.80539238
H	26.73947742	31.45323030	64.25783227
H	25.37373926	31.08767029	63.23109387

1JID-96 (Optimized)

N	-2.1082299	-0.2133324	-1.9647260
C	-2.3841135	1.1190782	-1.9801011

H	-3.4017364	1.4775985	-1.7554296
N	-1.3746911	1.8197033	-2.1740984
C	-0.3245966	0.9514932	-2.4194886
C	1.1161944	1.1073305	-2.4302612
O	1.7561351	2.1722460	-2.4298025
N	1.7767356	-0.0379237	-2.4514099
H	2.7949106	0.0644584	-2.4758185
C	1.1670814	-1.2740021	-2.4939060
N	2.0130083	-2.2326885	-2.5510221
H	2.9971685	-2.0877844	-2.3377868
H	1.6587516	-3.1713847	-2.3646331
N	-0.1131483	-1.5105970	-2.3313283
C	-0.7978075	-0.3493233	-2.2625512
C	-1.4042905	-1.2912796	4.0725485
H	-2.0510630	-1.1840423	3.1738720
O	-0.5693871	-0.1384702	4.2442075
P	0.0663333	0.5181732	2.8515330
O	0.6291044	1.8811673	3.1815347
O	-0.8364523	0.2217242	1.6548118
O	1.3125191	-0.6346183	2.8587879
C	2.1721017	-0.5335408	1.7143356
H	2.6765324	0.4617451	1.6655082
H	1.6006382	-0.6671251	0.7612303
H	-0.7932919	-2.2186317	3.9412260
H	2.9522534	-1.3263311	1.7846984
H	-2.0324972	-1.4103416	4.9847071
C	-3.0634467	-1.3161348	-1.7850387
H	-2.5553293	-2.2572729	-2.0654170
H	-3.3718739	-1.3812367	-0.7222341
H	-3.9545619	-1.1669351	-2.4270435

1JID-764

N	29.17100000	32.86000000	63.72900000
C	28.71400000	34.14900000	63.68400000
H	27.74000000	34.48000000	64.01100000
N	29.62500000	35.04500000	63.30900000
C	30.69600000	34.19500000	62.83300000
C	31.92800000	34.53700000	62.17600000
O	32.31100000	35.62800000	61.80500000
N	32.84600000	33.49300000	62.21000000
H	33.78800000	33.59500000	61.86100000
C	32.51200000	32.26200000	62.58600000
N	33.55700000	31.41800000	62.49300000
H	34.45300000	31.78000000	62.20000000
H	33.35000000	30.43600000	62.37800000
N	31.27700000	31.91900000	63.07700000
C	30.43200000	32.89100000	63.19800000
O	33.60300000	34.54600000	68.66400000
P	33.72600000	34.89200000	67.10600000
O	34.41900000	36.22500000	67.02100000
O	32.34400000	34.80100000	66.64600000
O	34.59200000	33.82200000	66.32100000
C	35.98030457	33.87323622	65.98205268
H	36.38739780	32.88379020	65.99515513
H	36.09346052	34.29254777	65.00416028
H	36.49885627	34.48170830	66.69322486
C	33.00021218	33.31728067	69.07848238
H	33.11005270	33.20534571	70.13692729
H	31.96025323	33.32751573	68.82692597
H	33.47929378	32.49958954	68.58173089

C	28.65388621	31.72746182	64.51056932
H	28.64824714	30.84725826	63.90220274
H	29.27970428	31.56744725	65.36359164
H	27.65730477	31.94331579	64.83481074

1JID-764 (Optimized)

N	-1.7681760	-0.3536396	-2.9145996
C	-2.1352313	0.9861909	-3.0088616
H	-3.1630396	1.2697225	-3.2754963
N	-1.1491035	1.8192215	-2.7448653
C	-0.0809813	1.0049839	-2.4308543
C	1.2800660	1.3372989	-2.0632237
O	1.8632699	2.4095743	-1.9784560
N	2.0013561	0.1117791	-1.7848514
H	2.9517026	0.2785498	-1.4425672
C	1.5135656	-1.1718293	-1.8528772
N	2.3733753	-2.1926083	-1.4636247
H	2.9234865	-1.9746906	-0.6264005
H	1.8524257	-3.0652766	-1.3387455
N	0.3080885	-1.4660787	-2.3066919
C	-0.4390144	-0.3555920	-2.5405209
O	-1.3013123	-0.1754538	3.9288693
P	-0.2583101	0.6085598	2.8357043
O	0.3987205	1.7124456	3.6368390
O	-0.9867550	0.7258804	1.5117272
O	0.9118280	-0.6105336	2.5487746
C	2.2178446	-0.6922403	3.1254416
H	2.3136496	-1.5765646	3.8040548
H	2.9857386	-0.8002513	2.3184984
H	2.4489923	0.2290747	3.7104734
C	-2.0348606	-1.3629460	3.6179523
H	-2.8270772	-1.4967171	4.3884313
H	-2.5122141	-1.2921533	2.6105014
H	-1.3819938	-2.2717496	3.6189613
C	-2.6174741	-1.5240239	-3.0094911
H	-3.2038497	-1.5108328	-3.9506499
H	-1.9665476	-2.4185982	-2.9953861
H	-3.3129753	-1.5816876	-2.1464338

1JID-816

N	30.92800000	38.44300000	62.55400000
C	30.70500000	39.76600000	62.17400000
H	29.87700000	40.41400000	62.42100000
N	31.70700000	40.26900000	61.50600000
C	32.64600000	39.24200000	61.40300000
C	33.91000000	39.19100000	60.74800000
O	34.70000000	39.99700000	60.25500000
N	34.46100000	37.87000000	60.83400000
H	35.40500000	37.66400000	60.53800000
C	33.80600000	36.81000000	61.27200000
N	34.44800000	35.68900000	61.19000000
H	35.36000000	35.59100000	60.76800000
H	33.99000000	34.91300000	61.64600000
N	32.58900000	36.75100000	61.91300000
C	32.13400000	38.06300000	61.96800000
O	35.36900000	39.26900000	66.48100000
P	35.07700000	40.01900000	65.05900000
O	36.09700000	41.05200000	64.92700000
O	33.69900000	40.33000000	65.08000000

O	35.31200000	39.02700000	63.86000000
C	30.24032026	37.58607518	63.53056359
H	29.39620881	38.10777363	63.93082174
H	29.90988451	36.68987156	63.04834862
H	30.91431172	37.33683262	64.32335212
C	36.65112371	38.80463628	63.41033115
H	36.94487269	37.80313225	63.64613024
H	36.70076098	38.95097083	62.35154773
H	37.30973871	39.49342184	63.89685016
C	34.29594827	38.68532339	67.22449283
H	34.69324158	38.13332938	68.05054406
H	33.65239685	39.45865266	67.58877762
H	33.73929385	38.02725123	66.59047635

1JID-816 (Optimized)

N	-2.1199377	-0.0245807	-2.2331571
C	-2.3095443	1.3440280	-2.0542740
H	-3.3146193	1.7846011	-2.1147465
N	-1.1937816	1.9970569	-1.8028371
C	-0.2166999	1.0248658	-1.7829983
C	1.2106693	1.1270036	-1.5568020
O	1.9376863	2.0858848	-1.3363275
N	1.7975038	-0.1924011	-1.6701312
H	2.7947954	-0.1997361	-1.4403248
C	1.1341983	-1.3792260	-1.8559098
N	1.9027958	-2.5396496	-1.7923444
H	2.5786824	-2.5360235	-1.0210849
H	1.2933234	-3.3605031	-1.7360452
N	-0.1526057	-1.4574297	-2.1419677
C	-0.7668005	-0.2442596	-2.0646817
O	-0.4536155	-0.8729961	3.5939550
P	-0.1371669	0.5060107	2.6446749
O	0.7196688	1.4023061	3.5127839
O	-1.4044056	0.8716051	1.8969855
O	0.8878358	-0.1915901	1.4334809
C	-3.1276992	-1.0557393	-2.3711403
H	-3.8566835	-0.7898567	-3.1632104
H	-2.6173111	-1.9988546	-2.6440951
H	-3.6692436	-1.2119960	-1.4145973
C	2.2821318	-0.3222888	1.7228884
H	2.5712799	-1.3941029	1.8771593
H	2.8808332	0.0861173	0.8726882
H	2.5476894	0.2569323	2.6355790
C	-1.6636790	-1.6141981	3.4171328
H	-1.5051251	-2.5229855	2.7815714
H	-2.0278869	-1.9569338	4.4138835
H	-2.4423261	-0.9869885	2.9277929

1JID-840

N	31.21700000	37.18300000	64.28100000
C	31.03000000	38.52800000	64.08400000
H	30.21600000	39.15400000	64.41700000
N	31.96800000	38.99300000	63.34200000
C	32.76600000	37.86600000	62.96800000
C	33.85200000	37.78700000	62.08800000
O	34.59800000	38.70500000	61.70000000
N	34.28700000	36.50000000	61.80400000
H	34.83500000	36.38700000	60.96300000
C	33.66300000	35.41200000	62.33800000

N	34.20400000	34.23200000	62.00600000
H	35.07800000	34.26900000	61.50100000
H	33.78400000	33.41300000	62.42100000
N	32.68500000	35.50200000	63.21600000
C	32.23200000	36.75200000	63.42800000
O	35.86500000	36.79600000	68.14300000
P	35.47800000	37.67100000	66.87900000
O	36.52100000	38.73100000	66.67400000
O	34.13400000	38.11500000	66.90100000
O	35.73900000	36.61000000	65.63100000
C	37.08742895	36.20300166	65.38403499
H	37.21993460	35.19200745	65.70843128
H	37.29449034	36.27150222	64.33649823
H	37.75682584	36.84095827	65.92238356
C	34.85579876	36.20780287	68.96788605
H	35.31504045	35.56577743	69.69024357
H	34.31202295	36.97966905	69.47130483
H	34.18519657	35.63784276	68.35933186
C	30.39113175	36.34933134	65.16634637
H	29.83021952	35.65253541	64.57918973
H	31.02283358	35.81607657	65.84567850
H	29.71920017	36.97256228	65.71860641

1JID-840 (Optimized)

N	-2.0853577	-0.3909324	-1.8612505
C	-2.5130124	0.8990716	-2.0504707
H	-3.5732532	1.1747177	-1.9655010
N	-1.5014427	1.6837041	-2.1382864
C	-0.3863619	0.8806492	-2.1671306
C	1.0072455	1.1615234	-2.2393724
O	1.5280835	2.2867802	-2.2856227
N	1.8649364	0.0730640	-2.3160050
H	2.8537343	0.3162285	-2.4033206
C	1.3886623	-1.2033281	-2.2684408
N	2.3272963	-2.1580135	-2.3217978
H	3.2366649	-1.9383308	-1.9113600
H	1.9780680	-3.0778120	-2.0431665
N	0.1131584	-1.5277459	-2.0814047
C	-0.7286521	-0.4535474	-2.0179605
O	-0.1462801	-0.5741258	3.8724338
P	-0.1150253	0.5196045	2.5746953
O	0.4604732	1.8077541	3.1205927
O	-1.3875284	0.3653043	1.7564928
O	1.0770440	-0.2812643	1.6864955
C	2.4153714	-0.3464214	2.1860295
H	2.5130558	-1.0930339	3.0130178
H	3.0890424	-0.6512292	1.3515422
H	2.7450834	0.6430552	2.5806318
C	-1.2235956	-1.5078345	3.9841462
H	-0.8983130	-2.5428174	3.7067720
H	-1.5847380	-1.5391348	5.0399857
H	-2.0623762	-1.2189851	3.3122767
C	-2.9753882	-1.5343692	-1.6136662
H	-2.5015512	-2.4548382	-2.0051971
H	-3.1322548	-1.6477279	-0.5212365
H	-3.9478945	-1.3740253	-2.1182015

1JID-1037

N	32.01400000	39.73100000	62.83700000

C	32.13500000	41.07700000	62.59900000
H	31.57600000	41.83500000	63.12800000
N	33.11100000	41.39600000	61.72600000
C	33.59100000	40.17800000	61.30900000
C	34.68200000	39.80800000	60.46500000
O	35.48300000	40.48100000	59.76500000
N	34.74200000	38.44700000	60.27300000
H	35.44200000	38.00700000	59.69400000
C	34.14800000	37.54500000	61.08100000
N	34.44200000	36.26200000	60.90000000
H	35.24700000	35.98400000	60.35700000
H	33.88600000	35.58300000	61.40100000
N	33.06100000	37.80800000	61.86800000
C	32.93700000	39.16400000	61.98100000
O	37.48800000	38.77900000	66.66500000
P	37.46500000	38.96700000	65.13000000
O	38.78500000	39.51800000	64.81700000
O	36.22600000	39.61200000	64.77900000
O	37.32400000	37.47100000	64.46000000
C	36.34695851	38.28896343	67.37407579
H	36.58363564	37.34712974	67.82333433
H	36.07284647	38.98863077	68.13580291
H	35.53060745	38.16445921	66.69365705
C	38.36088259	36.53135616	64.75469295
H	38.14292951	35.59854517	64.27797487
H	39.29554294	36.90667350	64.39351858
H	38.42002421	36.38575995	65.81308985
C	31.21767140	39.05672176	63.87242726
H	30.57758389	38.33184009	63.41445942
H	31.87199900	38.56845373	64.56405557
H	30.62379098	39.77907055	64.39244484

1JID-1037 (Optimized)

N	-2.6752218	-0.4007784	1.8536479
C	-2.9161488	0.9302926	2.1815967
H	-3.0080829	1.2406264	3.2318015
N	-3.0067315	1.7224740	1.1324714
C	-2.7809806	0.8920823	0.0535845
C	-2.6995006	1.1870652	-1.3618732
O	-2.8824348	2.2206781	-1.9930116
N	-2.3253377	-0.0196002	-2.0711570
H	-2.2005486	0.1325256	-3.0758506
C	-2.1525124	-1.2766141	-1.5384805
N	-1.8170306	-2.2973236	-2.4205121
H	-1.1525051	-2.0239066	-3.1506027
H	-1.4790906	-3.1152238	-1.9050980
N	-2.3337683	-1.5514048	-0.2606370
C	-2.5854634	-0.4396424	0.4782183
O	4.0122240	0.1114741	1.0979453
P	2.7896893	0.6882096	0.0761861
O	3.4403510	1.7984238	-0.7175349
O	1.4833197	0.7417493	0.8438157
O	2.6306019	-0.6882488	-0.9148509
C	3.7528616	-0.9217907	2.0518818
H	3.7536735	-1.9374958	1.5811466
H	4.5492402	-0.8959441	2.8298954
H	2.7575608	-0.7768488	2.5346577
C	3.7343862	-1.1763029	-1.6818994
H	3.3560396	-1.9312420	-2.4089774
H	4.2359006	-0.3504087	-2.2413639

H	4.5114450	-1.6618493	-1.0402048
C	-2.3144953	-1.4879439	2.7438967
H	-2.5494877	-2.4476453	2.2461999
H	-1.2269841	-1.4636965	2.9647164
H	-2.8871019	-1.4109966	3.6886247

1JID-1318

N	27.98700000	39.90200000	30.17900000
C	29.26400000	39.50700000	29.82500000
H	29.46700000	38.53600000	29.39800000
N	30.15500000	40.48300000	29.95900000
C	29.39700000	41.53100000	30.44800000
C	29.73100000	42.91900000	30.64000000
O	30.81400000	43.46400000	30.45800000
N	28.66200000	43.70700000	31.11600000
H	28.82400000	44.64800000	31.44500000
C	27.40900000	43.26300000	31.32100000
N	26.63100000	44.09900000	31.96700000
H	26.88100000	45.01700000	32.30500000
H	25.80200000	43.62700000	32.29900000
N	27.04800000	41.97600000	31.12300000
C	28.10700000	41.17300000	30.62700000
O	27.22700000	44.02200000	25.94300000
P	27.77800000	44.42900000	27.44000000
O	29.06400000	45.13100000	27.32000000
O	27.71400000	43.17700000	28.19900000
O	26.78900000	45.49300000	28.12600000
C	26.72608969	39.23930284	29.81590545
H	26.09518765	39.17631074	30.67781879
H	26.23265843	39.80483710	29.05328064
H	26.93261792	38.25438945	29.45232370
C	25.96645163	43.38256189	25.72612051
H	25.27854884	44.08450710	25.30308070
H	26.09481942	42.55976145	25.05424170
H	25.58277949	43.02495663	26.65875880
C	26.74151515	46.89632879	27.85523534
H	25.80802555	47.29377042	28.19514314
H	27.54454510	47.38547605	28.36589947
H	26.83644420	47.05978266	26.80206332

1JID-1318 (Optimized)

N	-0.6025201	-3.0028676	-1.2885051
C	0.7156718	-3.4048050	-1.5049990
H	0.9798808	-4.0142536	-2.3811749
N	1.5476950	-2.9959174	-0.5669487
C	0.7545329	-2.2839215	0.3113668
C	1.1039071	-1.3889915	1.4058993
O	2.2100753	-1.1437354	1.8826785
N	-0.0446812	-0.8195310	1.9947461
H	0.1794809	-0.1276869	2.7141263
C	-1.3004440	-0.9697982	1.5368711
N	-2.2349708	-0.5392890	2.3510071
H	-2.0424967	0.3444309	2.8337845
H	-3.1594993	-0.5223361	1.9106184
N	-1.6439433	-1.6775102	0.4697433
C	-0.5806781	-2.2684802	-0.1196510
O	0.4318503	2.9164453	-1.8437142
P	0.5584412	2.1420726	-0.3517722
O	1.8641796	2.6340019	0.2281108

O	0.1080542	0.7018701	-0.5388163
O	-0.7074768	2.8081643	0.5669668
C	-1.7524466	-3.1776409	-2.1479813
H	-1.8971817	-4.2460631	-2.4125868
H	-2.6409035	-2.8131724	-1.5972063
H	-1.6529865	-2.5843674	-3.0814182
C	-0.6878992	2.8769269	-2.7322520
H	-1.5408115	3.4985387	-2.3624466
H	-0.3615032	3.2750574	-3.7180999
H	-1.0647647	1.8343591	-2.8664485
C	-0.6230697	4.0436233	1.2821008
H	-1.2326509	3.9662989	2.2122863
H	0.4349806	4.2675005	1.5538259
H	-1.0179143	4.9025771	0.6843366

1JID-1396

N	28.14600000	32.81900000	29.64600000
C	29.47200000	32.65800000	29.44000000
H	29.93000000	31.78400000	29.00000000
N	30.26600000	33.71900000	29.61100000
C	29.33800000	34.69000000	29.94800000
C	29.36100000	36.10600000	29.98000000
O	30.29000000	36.87200000	29.92200000
N	28.16400000	36.76000000	30.29400000
H	28.11100000	37.76400000	30.39600000
C	27.02900000	36.07300000	30.37900000
N	25.93000000	36.73700000	30.62200000
H	26.02600000	37.74100000	30.66800000
H	25.08300000	36.22300000	30.81700000
N	26.87800000	34.77000000	30.17300000
C	28.08300000	34.16300000	29.87700000
O	28.47600000	36.06500000	24.99700000
P	28.68600000	36.41100000	26.49100000
O	29.94200000	37.15400000	26.51200000
O	28.54600000	35.19600000	27.29500000
O	27.41400000	37.38900000	26.87100000
C	27.00428111	31.94911894	29.32869111
H	27.35702455	30.96243208	29.11207832
H	26.33894647	31.91423365	30.16595620
H	26.48582558	32.33751266	28.47707255
C	27.37196167	35.25847521	24.57805237
H	26.72460536	35.83707801	23.95271358
H	27.73483513	34.41356582	24.03092260
H	26.83034624	34.92129844	25.43704280
C	27.23398230	38.70742507	26.34731215
H	26.23437125	39.03535828	26.54260055
H	27.92637223	39.37503381	26.81613490
H	27.40650494	38.69839656	25.29135078

1JID-1396 (Optimized)

N	-0.3882575	-1.4203973	2.7646908
C	0.9103649	-1.9333767	2.6786215
H	1.1283167	-2.9557060	3.0215938
N	1.7889864	-1.0689484	2.2181228
C	1.0413795	0.0645948	1.9562032
C	1.3085236	1.1588987	1.0472843
O	2.3680571	1.3935307	0.4650996
N	0.2752631	2.0941778	0.9178750
H	0.4524970	2.7942700	0.1928013

C	-0.9411236	1.8144604	1.3756749
N	-1.8842437	2.6983299	1.1832039
H	-1.8350023	3.2820470	0.3449812
H	-2.8190675	2.3270569	1.3621734
N	-1.3093019	0.7205340	2.0381296
C	-0.2976242	-0.1369461	2.2745486
O	-0.0997581	-1.5265511	-3.0049122
P	0.4000101	-0.3967297	-1.8796304
O	1.7685051	0.0437822	-2.3312332
O	-0.0407620	-0.8619411	-0.5014545
O	-0.7239349	0.8030510	-2.2459305
C	-1.6212568	-2.1429920	2.9802164
H	-1.5110902	-2.8655472	3.8146133
H	-2.4162317	-1.4136749	3.2286344
H	-1.9274485	-2.6867821	2.0609577
C	-1.3593073	-2.1937202	-2.8894953
H	-2.2108225	-1.5260221	-3.1731889
H	-1.3555674	-3.0729148	-3.5715096
H	-1.5382329	-2.5346932	-1.8430831
C	-0.7408514	1.5962382	-3.4356649
H	-1.3788782	2.4919111	-3.2585872
H	0.2882746	1.9255183	-3.7146705
H	-1.1612845	1.0333269	-4.3057325

1JID-2284

N	37.91800000	22.82000000	40.43100000
C	38.90100000	22.95600000	41.39400000
H	38.91900000	22.38500000	42.31000000
N	39.78600000	23.91800000	41.18700000
C	39.32700000	24.40500000	39.95300000
C	40.01400000	25.37400000	39.09600000
O	41.02600000	25.99300000	39.47100000
N	39.40300000	25.47800000	37.87200000
H	39.80000000	26.07600000	37.16100000
C	38.31500000	24.75300000	37.50400000
N	37.80000000	25.04800000	36.34700000
H	38.23600000	25.80800000	35.84500000
H	36.99500000	24.56600000	35.97300000
N	37.71400000	23.84800000	38.21500000
C	38.26500000	23.72300000	39.46800000
O	42.33800000	21.19300000	37.08400000
P	42.00300000	22.76400000	37.43400000
O	43.19000000	23.53800000	37.76400000
O	40.90100000	22.64500000	38.39900000
O	41.27900000	23.43500000	36.15100000
C	42.03895989	23.97515307	35.06675113
H	41.73371417	23.51209144	34.15171070
H	41.87251018	25.03034289	35.00535935
H	43.07929644	23.78719519	35.23189257
C	41.33910078	20.18185686	36.92686979
H	41.77437599	19.31872811	36.46812777
H	40.94652950	19.91619225	37.88614561
H	40.54896904	20.55406096	36.30876304
C	37.00038420	21.70674533	40.14896224
H	36.87716882	21.11385616	41.03111892
H	36.05095009	22.09539121	39.84494045
H	37.40510958	21.10066073	39.36553463

1JID-2284 (Optimized)

N	-2.9482812	-1.1923682	-0.3360597
C	-3.0164757	-1.5830640	1.0027134
H	-3.5684072	-2.4879002	1.2956247
N	-2.3852596	-0.7594527	1.8181596
C	-1.8569757	0.2083551	0.9903693
C	-0.9103985	1.2812136	1.2486509
O	-0.4189220	1.6098552	2.3317291
N	-0.6192127	2.0238802	0.1324205
H	0.1395748	2.6944902	0.2784119
C	-1.0562422	1.7179478	-1.1166606
N	-0.7740345	2.5830875	-2.0455261
H	0.1385504	3.0446757	-1.9938234
H	-0.9805802	2.2394822	-2.9865908
N	-1.8482848	0.6992482	-1.4240696
C	-2.1976529	-0.0345490	-0.3410326
O	2.9483658	-1.8029147	-0.3413087
P	2.0450408	-0.5892553	0.4095914
O	2.7649120	-0.3075274	1.7057437
O	0.5806753	-0.9231019	0.1848885
O	2.2861026	0.7459427	-0.6298689
C	3.4288860	1.6043331	-0.5839117
H	4.2357610	1.2663147	-1.2804482
H	3.1219129	2.6329591	-0.8881211
H	3.8513004	1.6403796	0.4469846
C	2.5035330	-2.4694514	-1.5256872
H	3.0158625	-3.4558117	-1.5864735
H	1.3998949	-2.6227531	-1.5053961
H	2.7498100	-1.8890289	-2.4500674
C	-3.3903337	-1.9128337	-1.5087416
H	-4.4413581	-2.2518157	-1.3968536
H	-3.3177520	-1.2296583	-2.3770355
H	-2.7464724	-2.7965363	-1.7076194

1JID-3652

N	45.96800000	26.64800000	40.34400000
C	44.76100000	25.91600000	40.21100000
H	44.37500000	25.55100000	39.27100000
N	44.24000000	25.52100000	41.31900000
C	44.95400000	26.20300000	42.25400000
C	44.64400000	26.48000000	43.65500000
O	43.60700000	26.28600000	44.27300000
N	45.66100000	27.22900000	44.22700000
H	45.51300000	27.54500000	45.17500000
C	46.88000000	27.48700000	43.67900000
N	47.57500000	28.37600000	44.28700000
H	47.15400000	28.67600000	45.15400000
H	48.24200000	28.81800000	43.67100000
N	47.06500000	27.41600000	42.35800000
C	46.10300000	26.76700000	41.70700000
O	48.57600000	22.53300000	43.31400000
P	47.45100000	23.39200000	44.15100000
O	46.68900000	22.47500000	44.99100000
O	46.76700000	24.25700000	43.14900000
O	48.33600000	24.30900000	45.16000000
C	46.95823673	27.00844617	39.31910499
H	47.12413939	28.06530919	39.33952231
H	47.87814616	26.49911412	39.51721483
H	46.59320885	26.72328065	38.35456609
C	48.67472387	24.00171457	46.51489549
H	49.63860907	24.40685331	46.74224527

H	47.94476452	24.42845171	47.17060484
H	48.69424865	22.93991188	46.64563886
C	49.41130044	23.14527832	42.32795313
H	50.22648700	22.49223969	42.09576479
H	48.84014830	23.33152508	41.44251686
H	49.79228078	24.07020836	42.70776677

1JID-3652 (Optimized)

N	-0.3886536	-1.4195847	2.7651399
C	0.9096799	-1.9328982	2.6793373
H	1.1274500	-2.9549842	3.0229596
N	1.7887254	-1.0687436	2.2189073
C	1.0413289	0.0649978	1.9565881
C	1.3085236	1.1588987	1.0472843
O	2.3679383	1.3932841	0.4645955
N	0.2752631	2.0941778	0.9178750
H	0.4519138	2.7932135	0.1915122
C	-0.9411236	1.8144604	1.3756749
N	-1.8842437	2.6983299	1.1832039
H	-1.8341497	3.2834131	0.3459377
H	-2.8192959	2.3269996	1.3607570
N	-1.3093798	0.7209323	2.0385306
C	-0.2977109	-0.1363954	2.2748954
O	-0.0997581	-1.5265511	-3.0049122
P	0.3997812	-0.3968176	-1.8791565
O	1.7686243	0.0434914	-2.3297701
O	-0.0418449	-0.8621161	-0.5011692
O	-0.7239349	0.8030510	-2.2459305
C	-1.6220851	-2.1408279	2.9820970
H	-1.5116675	-2.8638052	3.8160449
H	-2.4158792	-1.4107041	3.2318367
H	-1.9302142	-2.6839597	2.0631315
C	-1.3593073	-2.1937202	-2.8894953
H	-2.2091043	-1.5331513	-3.1941285
H	-1.3478550	-3.0856625	-3.5546975
H	-1.5469980	-2.5154766	-1.8385556
C	-0.7408514	1.5962382	-3.4356649
H	-1.4017380	2.4767429	-3.2673859
H	0.2844357	1.9485691	-3.7005735
H	-1.1352221	1.0242784	-4.3120544

1JID-5743

N	28.57500000	52.65300000	41.71400000
C	29.05700000	53.07100000	42.92700000
H	29.74600000	53.87700000	43.13500000
N	28.48900000	52.32700000	43.87800000
C	27.59900000	51.41800000	43.30500000
C	26.67300000	50.47300000	43.81800000
O	26.39900000	50.18400000	45.00000000
N	25.86100000	49.87900000	42.84000000
H	25.22800000	49.18500000	43.20900000
C	26.18000000	49.96100000	41.49800000
N	25.62800000	49.09500000	40.62600000
H	25.11500000	48.32000000	41.02000000
H	25.98100000	49.20400000	39.68600000
N	26.98800000	50.87300000	40.96000000
C	27.68700000	51.59200000	41.91500000
C	23.27400000	54.88100000	41.46200000
H	24.03700000	54.51300000	40.77600000

O	23.11700000	54.31500000	42.76300000
P	23.79700000	52.90100000	42.85200000
O	23.26000000	52.38600000	44.10800000
O	25.26900000	53.00300000	42.75900000
O	23.19700000	52.04100000	41.69000000
C	21.94600000	51.45700000	41.92100000
H	21.17600000	52.19300000	42.15000000
H	22.08800000	50.70900000	42.70000000
H	23.24116744	55.95011024	41.43327151
H	22.34519310	54.75312577	40.94637811
H	21.66225746	50.88206584	41.06435487
C	28.91699240	53.13524770	40.36812543
H	29.35643089	52.33870781	39.80481555
H	28.03012316	53.47409440	39.87462177
H	29.61335632	53.94396461	40.44528921

1JID-5743 (Optimized)

N	2.9219831	1.2742549	-0.5279253
C	3.2568921	1.5727855	0.7944420
H	3.8300532	2.4789646	1.0371699
N	2.8309840	0.6698676	1.6576317
C	2.1667772	-0.2580636	0.8795342
C	1.3071655	-1.3662197	1.2429288
O	1.0914016	-1.7994310	2.3766054
N	0.6958664	-2.0074328	0.1548923
H	0.0081169	-2.7215631	0.4027025
C	1.0294043	-1.6840776	-1.1465080
N	0.7326350	-2.5392054	-2.1443699
H	-0.1438616	-3.0625887	-2.0498727
H	0.8061398	-2.0756183	-3.0536378
N	1.7148970	-0.6105601	-1.5032764
C	2.2136119	0.0940299	-0.4673474
C	-2.9792964	2.3734058	-0.8005601
H	-1.9318085	2.6649361	-1.0430706
O	-3.0371338	1.6272120	0.4149463
P	-1.8671387	0.4069978	0.4988538
O	-2.1886317	-0.4472850	1.7034526
O	-0.5136546	0.9873850	0.1327083
O	-2.3733319	-0.4096959	-0.9038329
C	-3.4524481	-1.2972642	-0.8192832
H	-4.4408062	-0.7773292	-0.9168559
H	-3.4631107	-1.8350824	0.1577935
H	-3.5960324	3.2925150	-0.6771223
H	-3.3756422	1.7910501	-1.6667673
H	-3.3860622	-2.0427438	-1.6486152
C	3.0738489	2.0987001	-1.7057141
H	2.9876141	1.4481728	-2.5975872
H	2.2742519	2.8679390	-1.7612103
H	4.0640180	2.5981146	-1.7080462

1JID-4787

N	42.59800000	22.23100000	47.02000000
C	42.39700000	22.13400000	45.60300000
H	43.10300000	21.66700000	44.93200000
N	41.28000000	22.63800000	45.14800000
C	40.67100000	23.13000000	46.30700000
C	39.33500000	23.72000000	46.50100000
O	38.43900000	23.93400000	45.73800000

N	39.17700000	24.15000000	47.81000000
H	38.30800000	24.62100000	48.02000000
C	40.07900000	23.98700000	48.78200000
N	39.70000000	24.57400000	49.93400000
H	38.77800000	24.97700000	50.01700000
H	40.34800000	24.65800000	50.70500000
N	41.27400000	23.40700000	48.64800000
C	41.44700000	22.83800000	47.43400000
C	40.35100000	16.96000000	49.94100000
H	41.00200000	17.81200000	49.74500000
O	39.08000000	17.00200000	49.36700000
P	38.90200000	17.79000000	47.97600000
O	37.46800000	18.02800000	47.79500000
O	39.59800000	17.13500000	46.84600000
O	39.55400000	19.22900000	48.31500000
C	38.84000000	20.34800000	48.90900000
H	37.93500000	20.36900000	48.30200000
H	39.29100000	21.32900000	48.75800000
H	40.30444431	16.86346541	51.00561901
H	40.89347002	16.06175418	49.73176156
H	38.76143776	20.28018132	49.97395474
C	43.68884480	21.72403038	47.86494936
H	44.18479643	22.54562991	48.33813663
H	43.28756682	21.07150471	48.61200105
H	44.38818714	21.18593795	47.25974127

1JID-4787 (Optimized)

N	2.1586040	1.9263146	0.2925722
C	2.4345848	2.0414879	-1.1103778
H	3.4603083	1.8642965	-1.4657303
N	1.4134483	2.3348905	-1.8719059
C	0.3565484	2.4294133	-0.9652061
C	-1.0753934	2.6528909	-1.1726916
O	-1.7033049	2.7602807	-2.2268057
N	-1.7403316	2.7881676	0.0368134
H	-2.7562115	2.8678502	-0.0608735
C	-1.1696136	2.6745973	1.2394521
N	-2.0326900	2.9346502	2.2408439
H	-2.7948957	3.5922756	2.0862674
H	-1.6319738	2.9411674	3.1790209
N	0.0839239	2.3619132	1.4949560
C	0.8089871	2.2089328	0.3566646
C	0.7445982	-4.1695160	1.7778762
H	0.6613374	-3.1924680	2.3207516
O	-0.2474816	-4.3322604	0.8104192
P	-0.2576572	-3.3944727	-0.5969332
O	-1.6415177	-3.5548081	-1.1911118
O	1.0655104	-3.5798044	-1.3082970
O	-0.1371928	-1.9043408	0.1907808
C	-1.2878445	-1.1169604	0.6040917
H	-2.2174816	-1.5381142	0.1590970
H	-1.1562233	-0.0678032	0.2516971
H	0.6612428	-4.9913105	2.5286161
H	1.7737511	-4.2033814	1.3375941
H	-1.3824903	-1.1062528	1.7164613
C	3.0383174	1.5532838	1.4096454
H	2.5882565	1.9465686	2.3406194
H	3.1199424	0.4499834	1.4852498
H	4.0425979	1.9948301	1.2604823

1JID-4788

N	43.13200000	23.41800000	48.00300000
C	43.16500000	23.27800000	46.62500000
H	43.89600000	22.73300000	46.04700000
N	42.15300000	23.76800000	45.96400000
C	41.39500000	24.37200000	46.99200000
C	40.15800000	24.98100000	47.05600000
O	39.34500000	25.20000000	46.15400000
N	39.63500000	25.28400000	48.30400000
H	38.74400000	25.75700000	48.36100000
C	40.37600000	25.16400000	49.44900000
N	39.94100000	25.71400000	50.51300000
H	38.94800000	25.88600000	50.45600000
H	40.57400000	25.65000000	51.29800000
N	41.57900000	24.51100000	49.50600000
C	41.99900000	24.08600000	48.20900000
O	38.83100000	18.29500000	49.55000000
P	38.46600000	19.03600000	48.30400000
O	37.00100000	19.26400000	48.47500000
O	39.00400000	18.33100000	47.18100000
O	39.28700000	20.40600000	48.41200000
C	38.76312322	21.59789424	49.00347379
H	39.52052038	22.06311541	49.59914047
H	38.45175225	22.27148898	48.23261826
H	37.92510543	21.35091487	49.62123376
C	40.19831205	17.98383927	49.83024412
H	40.69777502	18.86043901	50.18662824
H	40.24419307	17.21878680	50.57689641
H	40.67606126	17.63946554	48.93690072
C	43.83543899	22.64288600	49.03512009
H	44.65164004	22.11368874	48.58940033
H	44.20866884	23.30639621	49.78702014
H	43.15803486	21.94437466	49.48021058

1JID-4788 (Optimized)

N	2.1062819	2.3549706	0.2108704
C	2.3648022	2.3744748	-1.1501439
H	3.4021052	2.2471744	-1.4970682
N	1.3305164	2.4647460	-1.9396178
C	0.2996665	2.5852396	-0.9925136
C	-1.1077908	2.6358154	-1.1215398
O	-1.7441730	2.7303759	-2.1834153
N	-1.8591907	2.5744472	0.0422790
H	-2.8641132	2.6744169	-0.1244186
C	-1.2840797	2.6610028	1.2817392
N	-2.0379823	2.8774060	2.2860407
H	-3.0438723	2.7182320	2.2430459
H	-1.6084120	2.7698624	3.2059783
N	0.0229397	2.4815718	1.4675687
C	0.7473520	2.4757836	0.3218625
O	0.2140433	-4.2085718	0.9010030
P	-0.3416203	-3.5928110	-0.5523738
O	-1.8215512	-3.9160160	-0.6133260
O	0.6852068	-3.8595222	-1.6322917
O	-0.1268545	-1.9985332	-0.0781902
C	-1.1731995	-1.1917603	0.4687828
H	-1.0425537	-1.0493055	1.5718194
H	-1.1584648	-0.1843166	-0.0106986
H	-2.1643013	-1.6696172	0.2918725

C	1.5389343	-3.9536432	1.3748966
H	1.6616956	-2.8896725	1.6987831
H	1.7373372	-4.6200401	2.2445812
H	2.2988644	-4.1495963	0.5805463
C	2.9379837	1.8434289	1.3097322
H	3.9840650	2.1847540	1.1875947
H	2.5261054	2.2303787	2.2600261
H	2.9083860	0.7347200	1.3294363

1JID-4802

C	41.78200000	22.34500000	51.93500000
H	41.44200000	22.90500000	52.80600000
N	41.17200000	22.88800000	50.63800000
C	41.42800000	22.58800000	49.33600000
H	42.22400000	21.92100000	49.03800000
N	40.45200000	22.88000000	48.51600000
C	39.52500000	23.49300000	49.33500000
C	38.30800000	24.13400000	49.00800000
O	37.77800000	24.19000000	47.88400000
N	37.70500000	24.81400000	50.04700000
H	36.71700000	24.98400000	49.92200000
C	38.25900000	24.98000000	51.23000000
N	37.46400000	25.55900000	52.13500000
H	36.48200000	25.70200000	51.94600000
H	37.84800000	25.59100000	53.06800000
N	39.36400000	24.36500000	51.63700000
C	39.94500000	23.57500000	50.66900000
C	37.97900000	17.61900000	51.92400000
H	38.68400000	18.43900000	52.06300000
O	37.00500000	17.86100000	50.88900000
P	37.30500000	18.78900000	49.66000000
O	36.06400000	19.10900000	49.00300000
O	38.26000000	18.05200000	48.83200000
O	38.00500000	20.11300000	50.21700000
C	37.31700000	21.23300000	50.87700000
H	36.73900000	21.79600000	50.14400000
H	38.17300000	21.81700000	51.21700000
H	42.84293063	22.44104214	51.83449078
H	41.61142302	21.29561043	52.05576850
H	38.52381320	16.71257301	51.76130798
H	37.49750450	17.36873115	52.84618631
H	36.76826474	20.99384346	51.76390121

1JID-4802 (Optimized)

C	3.0796942	1.5262543	0.9838949
H	2.7785159	2.1725028	1.8296340
N	2.0870336	1.6625397	-0.0645047
C	2.1282316	1.1252479	-1.3497336
H	3.0293607	0.6169488	-1.7185403
N	1.0029702	1.2744821	-2.0153134
C	0.1563004	1.8995863	-1.1268347
C	-1.2535875	2.2041274	-1.2372863
O	-2.0444378	2.1169083	-2.1658744
N	-1.7289240	2.6784295	0.0501358
H	-2.7280586	2.9013956	0.0395879
C	-0.9704175	2.9744212	1.1588781
N	-1.6206502	3.5705718	2.2336453
H	-2.5818848	3.2605959	2.3973917
H	-1.0498836	3.5297363	3.0817711

N	0.3312129	2.7665690	1.2229957
C	0.8142096	2.1700445	0.0937508
C	1.0920317	-3.9867194	1.7695466
H	1.2134300	-2.9648682	2.2067185
O	-0.1159037	-4.0988704	0.9905862
P	-0.4824734	-3.2239433	-0.4175721
O	-1.9908814	-3.2813617	-0.5293056
O	0.5304540	-3.5732359	-1.4852104
O	-0.0087921	-1.6943762	0.1361725
C	-0.8325901	-0.7893767	0.9520627
H	-1.6373112	-0.3200623	0.3420885
H	-0.1610870	-0.0005894	1.3515737
H	4.0791528	1.8385677	0.6204726
H	3.1302053	0.4752878	1.3359468
H	2.0029860	-4.1944203	1.1552420
H	1.0392219	-4.7297902	2.5964240
H	-1.3143267	-1.3273850	1.8019219

1JID-4806

N	41.27000000	22.44700000	47.87200000
C	41.63100000	22.35400000	46.50600000
H	42.62800000	22.18500000	46.12800000
N	40.61000000	22.38500000	45.72600000
C	39.53100000	22.69000000	46.54600000
C	38.11500000	22.80400000	46.23100000
O	37.56000000	22.68900000	45.17400000
N	37.33800000	23.04700000	47.39600000
H	36.33500000	22.92200000	47.38900000
C	37.91100000	23.10800000	48.65400000
N	37.10500000	23.56100000	49.60900000
H	36.12900000	23.68300000	49.38000000
H	37.35400000	23.56000000	50.58800000
N	39.16300000	23.00600000	48.91900000
C	39.92000000	22.71000000	47.83600000
O	38.06500000	16.32000000	48.32400000
P	37.87100000	17.03400000	46.93500000
O	36.49700000	16.79600000	46.54700000
O	38.87900000	16.62200000	45.96800000
O	37.96400000	18.63000000	47.17000000
C	42.09636944	22.26829884	49.07453044
H	42.09682418	23.17369257	49.64476020
H	41.69543594	21.47327505	49.66790174
H	43.09835498	22.02785396	48.78624024
C	39.15843282	16.68880869	49.16856191
H	38.80718224	17.33669916	49.94429445
H	39.58502213	15.80950045	49.60415183
H	39.90125679	17.19618812	48.58918408
C	36.80681704	19.38464067	47.53924931
H	37.10422567	20.36414072	47.85074211
H	36.14850157	19.46202328	46.69928939
H	36.30186006	18.89241896	48.34400781

1JID-4806 (Optimized)

N	2.0345431	2.6843563	0.0686234
C	2.2412710	2.5548965	-1.3027509
H	3.2530802	2.6156570	-1.7272930
N	1.1315065	2.3493412	-1.9826875
C	0.1383174	2.3174230	-1.0258672
C	-1.2923842	2.1346333	-1.1594444

O	-2.0100141	1.9933372	-2.1395786
N	-1.8920671	2.1463076	0.1617568
H	-2.9002847	1.9700629	0.1396097
C	-1.2481381	2.3120208	1.3649451
N	-2.0272233	2.2491175	2.5117532
H	-2.7722564	1.5470197	2.4919130
H	-1.4449479	2.1643019	3.3488654
N	0.0436580	2.5699189	1.4652859
C	0.6755495	2.5355655	0.2619950
O	0.5291392	-4.0627154	1.0929970
P	0.0313721	-3.5611578	-0.4530603
O	-1.2837268	-4.2746082	-0.6932694
O	1.2416109	-3.5364105	-1.3592811
O	-0.2866264	-1.9211860	-0.1013209
C	3.0278126	2.8317293	1.1148026
H	3.6287154	3.7523726	0.9674984
H	2.4933505	2.8969909	2.0811328
H	3.7068646	1.9550802	1.1413109
C	1.5844415	-3.3859376	1.7808988
H	1.2915518	-2.3478954	2.0775358
H	1.8326649	-3.9647887	2.6993543
H	2.4995573	-3.3023755	1.1456150
C	-1.5458819	-1.4563106	0.3916970
H	-1.9237058	-0.6320237	-0.2586091
H	-2.2986656	-2.2791918	0.3897263
H	-1.4508874	-1.0561532	1.4353481

1JID-5975

N	34.17500000	49.07700000	57.37800000
C	34.53500000	50.05300000	56.47200000
H	34.41500000	51.10800000	56.67200000
N	35.16300000	49.65400000	55.39700000
C	35.17900000	48.25100000	55.58400000
C	35.66500000	47.21400000	54.82500000
O	36.25800000	47.26900000	53.73000000
N	35.43000000	45.97100000	55.32600000
H	35.79100000	45.16400000	54.83800000
C	34.85200000	45.73500000	56.53500000
N	34.97700000	44.51600000	56.92000000
H	35.67600000	43.98000000	56.42600000
H	34.36500000	44.11100000	57.61400000
N	34.37300000	46.65700000	57.34900000
C	34.56400000	47.89800000	56.79300000
O	40.43800000	48.84100000	59.68300000
P	40.06100000	49.31800000	58.17400000
O	40.88500000	48.51800000	57.27800000
O	40.07700000	50.75100000	57.95900000
O	38.51500000	48.82600000	58.00200000
C	33.73399595	49.16343286	58.77762309
H	32.84915908	48.57646836	58.90967001
H	34.50662687	48.79307463	59.41854137
H	33.52519909	50.18366934	59.02343073
C	39.64592858	49.29683318	60.78288135
H	39.02801982	48.49662720	61.13325136
H	40.28828974	49.62395359	61.57361796
H	39.02880760	50.11099661	60.46476250
C	38.25197022	47.50785077	57.51394474
H	37.21104233	47.28704621	57.62625890
H	38.51910561	47.45066000	56.47940721
H	38.82895035	46.79953901	58.07098011

1JID-5975 (Optimized)

N	-2.0351568	2.1430800	-0.0942234
C	-1.7738082	2.1775563	-1.4482988
H	-1.4902455	3.1340800	-1.9140735
N	-1.8136465	1.0356788	-2.0837031
C	-2.1793186	0.1851889	-1.0476385
C	-2.4369771	-1.2025143	-1.0184620
O	-2.3461829	-2.0044437	-1.9626094
N	-2.8470921	-1.6905275	0.1835879
H	-2.9521649	-2.7085563	0.1865524
C	-2.9222510	-0.9470572	1.3207176
N	-3.0661151	-1.6624688	2.3777419
H	-3.4112589	-2.6182314	2.3478584
H	-3.1148167	-1.1871187	3.2786163
N	-2.6392775	0.3393130	1.3890672
C	-2.3145104	0.8416995	0.1747465
O	4.1466439	-0.0881419	1.0893640
P	3.3951182	-0.2881823	-0.4295062
O	3.5180365	-1.7650970	-0.7389857
O	3.8033715	0.8756810	-1.3090488
O	1.8212518	0.0460040	0.0412842
C	-1.8082446	3.1354414	0.9662623
H	-2.4112730	2.8371371	1.8431190
H	-0.7364497	3.1521190	1.2506164
H	-2.1142473	4.1416949	0.6203849
C	4.0763443	1.1902488	1.7262904
H	3.0517037	1.4054449	2.1224623
H	4.7948587	1.2027217	2.5779038
H	4.3346202	2.0093150	1.0131924
C	0.9576722	-0.9779085	0.5420173
H	0.0870985	-0.4826997	1.0300626
H	0.5823168	-1.6398356	-0.2740479
H	1.4660217	-1.6296071	1.2920293

1DUL-4

N	18.96900000	26.70800000	36.26700000
C	19.40200000	27.47400000	35.26900000
H	19.75900000	27.12700000	34.31100000
N	19.22500000	28.76000000	35.42000000
C	18.58600000	28.82700000	36.69500000
C	17.94700000	29.85600000	37.39400000
O	17.83000000	30.96200000	37.06400000
N	17.57500000	29.54200000	38.61600000
H	17.53400000	30.25500000	39.33100000
C	17.81900000	28.35400000	39.16000000
N	17.73600000	28.13300000	40.43000000
H	17.25100000	28.83300000	40.97300000
H	17.93400000	27.22700000	40.82800000
N	18.22000000	27.28200000	38.48600000
C	18.68800000	27.58200000	37.26000000
O	13.16400000	28.55800000	36.35700000
P	14.52100000	29.03800000	37.09700000
O	14.61200000	30.53000000	36.86300000
O	15.58800000	28.22500000	36.51700000
O	14.16600000	28.71200000	38.59400000
C	18.89474031	25.24213414	36.34838048
H	19.53695815	24.89536619	37.13081785
H	17.88752533	24.94704434	36.55656306
H	19.20568453	24.81700154	35.41699658

C	12.92599720	27.14813595	36.33380481
H	12.03678667	26.92739250	36.88650452
H	12.80654689	26.82319717	35.32135905
H	13.75657213	26.63888536	36.77619505
C	13.23094120	29.49326623	39.34245715
H	12.92046817	28.94562516	40.20767143
H	13.69395137	30.40860508	39.64688511
H	12.37874494	29.71015205	38.73284902

1DUL-598

N	47.64600000	22.98000000	26.36100000
C	47.51200000	24.20300000	25.80900000
H	48.37900000	24.79400000	25.55300000
N	46.30000000	24.67100000	25.76200000
C	45.52000000	23.61900000	26.36500000
C	44.10900000	23.48700000	26.57000000
O	43.10500000	24.12100000	26.24900000
N	43.78800000	22.40800000	27.41000000
H	42.79800000	22.27700000	27.55600000
C	44.70000000	21.49100000	27.82000000
N	44.40800000	20.65300000	28.74600000
H	43.42200000	20.52700000	28.92900000
H	45.14600000	20.10800000	29.16900000
N	46.02400000	21.59300000	27.65600000
C	46.38400000	22.66600000	26.85700000
O	44.25100000	20.36900000	22.69900000
P	43.80300000	20.95400000	24.00900000
O	42.60800000	21.86000000	23.76800000
O	45.01500000	21.59700000	24.56400000
O	43.37300000	19.76500000	24.97000000
C	48.72366755	21.98477696	26.45620378
H	49.60191210	22.36303125	25.97608900
H	48.93704194	21.78752144	27.48599096
H	48.41647293	21.07936443	25.97582933
C	45.30384919	19.41774153	22.52147280
H	44.88818569	18.47602233	22.22943031
H	45.97257597	19.76361186	21.76115826
H	45.83858182	19.30180987	23.44099488
C	42.21084432	18.96435848	24.73920283
H	42.32342631	18.02165505	25.23269602
H	41.34871203	19.46776210	25.12418684
H	42.09080998	18.80457712	23.68803125

1DUL-1140

N	57.52400000	26.71700000	28.58900000
C	57.03700000	27.82300000	27.95200000
H	57.67400000	28.58700000	27.53300000
N	55.74400000	27.77000000	27.77900000
C	55.36900000	26.59500000	28.38200000
C	54.10400000	25.93600000	28.40000000
O	53.13900000	26.22800000	27.73700000
N	54.09200000	24.72400000	29.09300000
H	53.34000000	24.08000000	28.89000000
C	55.24800000	24.23700000	29.64300000
N	55.08400000	23.19500000	30.39800000
H	54.17800000	22.75200000	30.34600000
H	55.89000000	22.97600000	30.96600000
N	56.44300000	24.87900000	29.78600000

C	56.42300000	26.03000000	29.06400000
O	56.21300000	23.45800000	24.24700000
P	55.39000000	23.77600000	25.50500000
O	54.05800000	24.19900000	24.95100000
O	56.10000000	24.75300000	26.33500000
O	55.21500000	22.47500000	26.38500000
C	58.90063800	26.23372286	28.76847429
H	59.10284495	26.11243178	29.81217004
H	59.01622479	25.29340127	28.27114643
H	59.58488648	26.94356256	28.35274407
C	57.51990707	22.88819257	24.35751425
H	57.48767478	21.86123543	24.05881690
H	58.19677875	23.42274716	23.72427170
H	57.85316278	22.95423591	25.37214665
C	54.50231141	21.30201181	25.98366013
H	54.86872059	20.45780622	26.52951837
H	53.45951774	21.43181126	26.18523754
H	54.64542567	21.13872788	24.93592129

1DUL-1449

N	59.57100000	33.72200000	26.56200000
C	58.90800000	34.89300000	26.11600000
H	59.37000000	35.86800000	26.16300000
N	57.62300000	34.71400000	25.93400000
C	57.45800000	33.30400000	26.02000000
C	56.29900000	32.47200000	25.82200000
O	55.15400000	32.78200000	25.49100000
N	56.61100000	31.12100000	25.87100000
H	55.87000000	30.46700000	25.66200000
C	57.74900000	30.63100000	26.33900000
N	57.82600000	29.32400000	26.47000000
H	56.93000000	28.86400000	26.54900000
H	58.64800000	28.91100000	26.88600000
N	58.84100000	31.38100000	26.64300000
C	58.59400000	32.71800000	26.46500000
O	58.75100000	31.76700000	21.19300000
P	57.84200000	31.71700000	22.49400000
O	56.53500000	32.32300000	22.11600000
O	58.67100000	32.37000000	23.52500000
O	57.64900000	30.19800000	22.72600000
C	60.88000191	33.43924745	27.16818892
H	60.73907391	33.03410276	28.14844249
H	61.40826919	32.73263636	26.56276803
H	61.44547315	34.34519029	27.23459559
C	60.08090412	31.24796377	21.27580345
H	60.16258367	30.37642603	20.66045767
H	60.77474478	31.98924724	20.93818835
H	60.30048658	30.98984842	22.29072217
C	56.83332062	29.31493404	21.95155719
H	57.16875052	28.30804553	22.08780074
H	55.81533392	29.39798264	22.27047098
H	56.90554418	29.57801861	20.91692186

1DUL-1908

N	58.41300000	27.18600000	28.77000000
C	57.68400000	28.17000000	28.14500000
H	58.20400000	29.06000000	27.82200000
N	56.37600000	27.96100000	27.98800000
C	56.19300000	26.71400000	28.61300000

C	55.06300000	25.79600000	28.67500000
O	53.99500000	25.85300000	28.20000000
N	55.27400000	24.66800000	29.42700000
H	54.52600000	23.99400000	29.49500000
C	56.44600000	24.47600000	30.08000000
N	56.43800000	23.44000000	30.87900000
H	55.58300000	22.90200000	30.89200000
H	57.28500000	23.26000000	31.39900000
N	57.56600000	25.14400000	29.93900000
C	57.40100000	26.30000000	29.20000000
O	57.62800000	23.92100000	24.45300000
P	56.58200000	24.09700000	25.69500000
O	55.27900000	24.17500000	25.09600000
O	56.99300000	25.30100000	26.47000000
O	56.65000000	22.83600000	26.65900000
C	59.84614185	27.02167171	29.05284384
H	60.00240164	27.01580920	30.11135624
H	60.18809822	26.09712084	28.63670500
H	60.39109656	27.83247208	28.61634970
C	58.99812717	23.62488495	24.73578511
H	59.17750679	22.58187970	24.57810509
H	59.62793693	24.19716126	24.08714075
H	59.21413741	23.87404542	25.75370389
C	55.93471576	21.62339366	26.40829236
H	56.31187678	20.84905046	27.04314839
H	54.89474065	21.77464338	26.60947713
H	56.06231739	21.33915271	25.38465917

1DUL-1492

C	60.19700000	35.23200000	25.54300000
H	60.56400000	34.58800000	26.34300000
N	58.67300000	35.29100000	25.55400000
C	57.88200000	36.23100000	24.91100000
H	58.37300000	37.10200000	24.50500000
N	56.65500000	35.92400000	24.70000000
C	56.59900000	34.69000000	25.43800000
C	55.44700000	33.77600000	25.67800000
O	54.27800000	33.89800000	25.44800000
N	55.81700000	32.63500000	26.36900000
H	55.14200000	31.92100000	26.60200000
C	57.06700000	32.34900000	26.74000000
N	57.29100000	31.35200000	27.51400000
H	56.50700000	30.85700000	27.91300000
H	58.21800000	31.29500000	27.91100000
N	58.08800000	33.15100000	26.67200000
C	57.81200000	34.32700000	25.99500000
C	59.12100000	31.48700000	20.91500000
H	59.52000000	32.01700000	21.77900000
O	57.66300000	31.58900000	20.86900000
P	56.90500000	32.29000000	22.00300000
O	55.44100000	32.44100000	21.76100000
O	57.68200000	33.46700000	22.43600000
O	56.85700000	31.07000000	23.13000000
C	56.23400000	29.75900000	22.84800000
H	56.39700000	29.52100000	21.79600000
H	55.15400000	29.74300000	22.99300000
H	56.67852689	28.96338181	23.40861353
H	59.54691241	30.50551936	20.92894149
H	59.54681862	31.84493449	20.00096422
H	60.44749279	34.76789253	24.61200181

H	60.55540539	36.23963550	25.50958933

1DUL-1777

C	55.90100000	28.32300000	23.87300000
H	56.45500000	27.67300000	24.55100000
N	54.43200000	28.51800000	24.15300000
C	53.57000000	29.42700000	23.67900000
H	53.92100000	30.33500000	23.21200000
N	52.34600000	29.09200000	23.80700000
C	52.37700000	27.88300000	24.49600000
C	51.29400000	27.04100000	25.04700000
O	50.13800000	27.20700000	24.94600000
N	51.76800000	26.05000000	25.89500000
H	51.16400000	25.27700000	26.13600000
C	53.14500000	25.88300000	26.13200000
N	53.32300000	24.87800000	26.92200000
H	52.59400000	24.31300000	27.33400000
H	54.27500000	24.60700000	27.12400000
N	54.09200000	26.58900000	25.62600000
C	53.67600000	27.57400000	24.77100000
C	53.85500000	24.37300000	20.19800000
H	54.42600000	25.02400000	20.85900000
O	52.47200000	24.57700000	20.38000000
P	51.97800000	25.11900000	21.80400000
O	50.53200000	25.47200000	21.60800000
O	52.84700000	26.23700000	22.11800000
O	52.17300000	23.94700000	22.83000000
C	51.24200000	22.86400000	22.88800000
H	50.79100000	22.61000000	21.92900000
H	50.44400000	23.26700000	23.51200000
H	51.67003102	21.98537402	23.32355233
H	54.09632519	23.33599117	20.30418308
H	54.24093732	24.53199531	19.21277268
H	56.01198080	27.87053980	22.90974353
H	56.42248651	29.25726664	23.88288240

1DUL-1651

N	57.23700000	33.97700000	24.73700000
C	56.39200000	34.52100000	23.83900000
H	56.58700000	35.26500000	23.08100000
N	55.18200000	33.97400000	23.82500000
C	55.17000000	33.03800000	24.94400000
C	54.22700000	32.10600000	25.47300000
O	53.07300000	31.87500000	25.11000000
N	54.74700000	31.37000000	26.53700000
H	54.27500000	30.51500000	26.79100000
C	56.00800000	31.47300000	26.94100000
N	56.26300000	30.64000000	27.88800000
H	55.43500000	30.08500000	28.04800000
H	57.17100000	30.45400000	28.29000000
N	56.93600000	32.32000000	26.48700000
C	56.47000000	33.06900000	25.45600000
O	57.67500000	29.71500000	21.04300000
P	56.47200000	30.37200000	21.87500000
O	55.26900000	30.15100000	21.10500000
O	57.00400000	31.71300000	21.96500000
O	56.43400000	29.59700000	23.29200000
C	59.05121628	29.95598722	21.34771938
H	59.52153585	29.03579466	21.62510927

H	59.54123167	30.36184530	20.48744957
H	59.12063712	30.65064077	22.15860605
C	55.71455975	28.37405392	23.47006914
H	55.96540703	27.94757326	24.41879981
H	54.66330780	28.57017406	23.43392741
H	55.97664225	27.68934326	22.69072064
C	58.69418762	34.10687763	24.88065253
H	58.92945899	34.39413578	25.88416144
H	59.15936136	33.16893855	24.65978222
H	59.05441604	34.85209531	24.20257725

1DUL-2682

C	69.00100000	46.02400000	39.49100000
H	68.64100000	45.78100000	40.49000000
N	68.76100000	44.82300000	38.63200000
C	68.26300000	44.80900000	37.34900000
H	67.86900000	45.65100000	36.79900000
N	68.06800000	43.64400000	36.81400000
C	68.53900000	42.77200000	37.75300000
C	68.74200000	41.33300000	37.87200000
O	68.35800000	40.49400000	37.17300000
N	69.40600000	40.89500000	39.02400000
H	69.62800000	39.91000000	38.98300000
C	69.85000000	41.69200000	39.99300000
N	70.41100000	41.09800000	41.03600000
H	70.54000000	40.12100000	40.81700000
H	70.68500000	41.59400000	41.87200000
N	69.67300000	43.02700000	40.01700000
C	69.00300000	43.52600000	38.88900000
C	74.24500000	44.18600000	36.86100000
H	74.43600000	43.92500000	37.90200000
O	73.01700000	43.60300000	36.36900000
P	72.89900000	42.07700000	36.12300000
O	73.84700000	41.70200000	35.06700000
O	71.49600000	41.89400000	35.74500000
O	73.22400000	41.25600000	37.43900000
C	74.21700000	40.19000000	37.48200000
H	75.05600000	40.57900000	36.90500000
H	73.83400000	39.28700000	37.00800000
H	74.54005505	39.88956587	38.45682038
H	74.18930066	45.25415821	36.83209275
H	75.16408239	43.98433590	36.35156978
H	70.03990699	46.27793945	39.52397004
H	68.49498692	46.86710708	39.06907439

1DUL-3184

C	70.54400000	37.10600000	41.66400000
H	70.28200000	36.32100000	42.37300000
N	69.73700000	36.79700000	40.54200000
C	69.36500000	37.61400000	39.51200000
H	69.59600000	38.66200000	39.39300000
N	68.57500000	37.04700000	38.60500000
C	68.58300000	35.69000000	39.06300000
C	68.12000000	34.51000000	38.42800000
O	67.57000000	34.38700000	37.29600000
N	68.39100000	33.33400000	39.15400000
H	68.10400000	32.48100000	38.69700000
C	68.87900000	33.36300000	40.43700000
N	68.84400000	32.24100000	41.11000000

H	68.50500000	31.36600000	40.73700000
H	69.14900000	32.29000000	42.07200000
N	69.36700000	34.42300000	41.07500000
C	69.25900000	35.53900000	40.24800000
C	74.58700000	34.32000000	38.24100000
H	73.98500000	34.83900000	38.98800000
O	73.87900000	33.72900000	37.16500000
P	72.36100000	33.42400000	37.22200000
O	71.90700000	32.99200000	35.90900000
O	71.63100000	34.48600000	37.93700000
O	72.38100000	32.25000000	38.19200000
C	72.80000000	30.89300000	37.85000000
H	73.79400000	30.81400000	37.41100000
H	72.10100000	30.43600000	37.15000000
H	72.75983618	30.28050179	38.72643186
H	75.09738497	33.55792794	38.79204751
H	75.32711075	35.00467174	37.88272541
H	70.38974851	38.11422354	41.98740652
H	71.59361085	37.03539228	41.46847094

1DUL-344

N	34.29100000	14.08600000	44.36100000
C	33.07500000	14.74700000	44.26000000
H	32.46700000	14.56900000	43.38600000
N	32.73300000	15.56500000	45.24400000
C	33.81800000	15.45100000	46.03100000
C	34.07200000	15.94600000	47.36200000
O	33.43500000	16.70100000	48.09700000
N	35.20600000	15.42200000	47.89300000
H	35.44300000	15.74900000	48.81900000
C	36.16400000	14.67500000	47.26800000
N	37.12600000	14.33500000	48.00200000
H	37.15400000	14.73200000	48.93000000
H	37.54500000	13.44200000	47.78800000
N	35.97900000	14.24400000	46.04100000
C	34.77500000	14.57000000	45.53500000
O	31.80200000	11.40000000	48.49400000
P	32.64400000	12.61400000	48.97400000
O	31.88000000	13.24100000	50.00100000
O	33.09900000	13.41400000	47.79400000
O	33.92700000	11.97600000	49.63200000
C	34.86340679	12.97406114	43.58844422
H	35.85939605	13.22412662	43.28782379
H	34.88448134	12.09240718	44.19436678
H	34.26299261	12.79627949	42.72080576
C	32.34894976	10.26238517	47.82200411
H	32.48361412	9.46494203	48.52261094
H	31.67902557	9.94862333	47.04892256
H	33.29346556	10.52236827	47.39165693
C	34.07905891	11.72816372	51.03212687
H	34.97053672	11.16045900	51.19909596
H	34.14652410	12.65983246	51.55399066
H	33.23389418	11.17875582	51.39094095

1DUL-487

N	16.55200000	28.43700000	44.37400000
C	16.35400000	29.75500000	44.03200000
H	15.81400000	30.10600000	43.16500000
N	16.99600000	30.63000000	44.82400000

C	17.45900000	29.81100000	45.78300000
C	18.23900000	30.02000000	46.94700000
O	18.65100000	31.11300000	47.46200000
N	18.65200000	28.90900000	47.70300000
H	19.03700000	28.94800000	48.63600000
C	18.39300000	27.68200000	47.28000000
N	18.98200000	26.66800000	47.82500000
H	19.61600000	26.73100000	48.60900000
H	18.81600000	25.76000000	47.41700000
N	17.76300000	27.36800000	46.13100000
C	17.31500000	28.48800000	45.52200000
O	13.33700000	28.73300000	49.04900000
P	14.87200000	29.03700000	48.77100000
O	15.14500000	30.29900000	49.37200000
O	15.22100000	28.85300000	47.36700000
O	15.65800000	27.82200000	49.51600000
C	15.99475576	27.18320045	43.84639641
H	16.79304448	26.52393175	43.57621151
H	15.38745851	26.71969028	44.59556153
H	15.39815127	27.39335013	42.98337833
C	12.71781604	27.65523607	48.34193838
H	12.38415757	26.91589473	49.03975383
H	11.88093414	28.02623261	47.78796160
H	13.42505115	27.21714247	47.66903981
C	15.67844351	27.73102639	50.94295685
H	16.07923484	26.78262960	51.23417815
H	16.28923917	28.51394065	51.34154677
H	14.68215336	27.82843779	51.32086817

1DUL-672

N	18.06300000	27.61800000	46.47400000
C	17.60900000	28.90900000	46.28900000
H	16.90300000	29.16800000	45.51400000
N	17.97800000	29.77100000	47.20600000
C	18.75000000	28.95400000	48.08300000
C	19.25400000	29.14200000	49.40300000
O	19.23600000	30.12900000	50.10700000
N	19.88400000	27.99100000	49.86300000
H	20.21800000	27.80200000	50.79800000
C	19.95700000	26.81200000	49.18300000
N	20.72500000	25.91400000	49.75400000
H	21.38500000	26.25300000	50.43800000
H	20.87800000	25.02300000	49.30300000
N	19.48700000	26.58700000	48.00300000
C	18.91600000	27.68900000	47.52700000
O	14.62600000	27.21600000	50.65200000
P	16.15700000	27.67300000	50.65800000
O	16.30200000	28.90000000	51.49200000
O	16.55700000	27.72200000	49.22500000
O	16.95700000	26.55400000	51.33000000
C	17.02766125	26.38669909	52.74842074
H	16.73014799	25.39154818	53.00544313
H	18.03166008	26.55511670	53.07784731
H	16.37404805	27.08824929	53.22330714
C	14.23827076	26.04381856	49.93050384
H	13.23895001	25.77303008	50.20056242
H	14.28284063	26.24073318	48.87972405
H	14.90290272	25.24060596	50.17136452
C	17.66350962	26.42344544	45.71614326
H	18.53499770	25.94084356	45.32562571

H	17.14172872	25.74898401	46.36247635
H	17.02301704	26.71100344	44.90869069

1DUL-1338

N	17.85600000	37.09100000	55.45500000
C	18.41100000	37.02900000	56.69800000
H	18.21100000	37.73100000	57.49400000
N	19.28500000	36.11300000	56.85800000
C	19.25100000	35.40800000	55.61800000
C	19.90200000	34.21500000	55.14700000
O	20.78400000	33.50500000	55.65700000
N	19.56900000	33.91600000	53.84300000
H	20.03100000	33.11100000	53.44500000
C	18.79500000	34.70900000	53.03700000
N	18.90700000	34.45300000	51.74100000
H	19.59300000	33.81700000	51.35900000
H	18.40900000	35.18300000	51.25200000
N	18.09800000	35.71300000	53.48200000
C	18.35600000	36.01500000	54.78900000
O	15.07500000	32.22700000	56.31000000
P	16.52400000	32.57000000	55.82200000
O	17.38100000	31.77000000	56.74600000
O	16.75100000	34.02700000	55.79500000
O	16.61200000	31.96900000	54.34900000
C	16.75684296	37.93488833	54.96448365
H	17.06535906	38.43983551	54.07299829
H	15.90525813	37.32310815	54.75134453
H	16.49984533	38.65597995	55.71206582
C	13.94907151	32.87950335	55.71718939
H	13.34999118	32.15859117	55.20115801
H	13.36463913	33.34728623	56.48172713
H	14.29010665	33.62086923	55.02511161
C	16.67167015	30.56118909	54.10526262
H	16.48116738	30.37009086	53.06984475
H	17.64382175	30.19557717	54.36245745
H	15.93466954	30.06450269	54.70110885

1DUL-1425

N	21.14300000	27.09500000	56.82000000
C	21.39300000	28.11900000	57.73400000
H	20.64800000	28.89000000	57.86400000
N	22.51900000	28.04100000	58.32800000
C	23.09300000	26.90900000	57.73100000
C	24.36900000	26.34000000	57.98800000
O	25.14000000	26.62200000	58.88800000
N	24.63300000	25.20100000	57.27300000
H	25.51900000	24.75900000	57.47200000
C	23.81800000	24.79900000	56.22400000
N	24.19500000	23.80900000	55.39200000
H	25.17100000	23.58000000	55.51100000
H	23.68500000	23.56200000	54.55500000
N	22.64300000	25.34000000	55.94000000
C	22.33600000	26.42300000	56.71200000
O	21.51100000	23.14300000	60.89600000
P	22.77000000	23.85300000	60.12000000
O	23.81200000	24.02200000	61.18500000
O	22.22500000	25.02000000	59.44000000
O	23.42200000	22.72400000	59.17000000
C	24.16963184	21.61750487	59.68148330

H	23.67024151	20.70435080	59.43317112
H	25.14829004	21.61785210	59.24890036
H	24.24978057	21.70237517	60.74509658
C	20.27825750	22.79725586	60.25904874
H	19.78599256	22.03430829	60.82516378
H	19.65034902	23.66190797	60.20420653
H	20.47602959	22.43684776	59.27117618
C	19.98611901	26.76418621	55.97555426
H	20.25598175	26.86583167	54.94514546
H	19.68153359	25.75642921	56.16674412
H	19.17875858	27.42950143	56.20010889

1DUL-41

N	21.14300000	27.09500000	56.82000000
C	21.39300000	28.11900000	57.73400000
H	20.64800000	28.89000000	57.86400000
N	22.51900000	28.04100000	58.32800000
C	23.09300000	26.90900000	57.73100000
C	24.36900000	26.34000000	57.98800000
O	25.14000000	26.62200000	58.88800000
N	24.63300000	25.20100000	57.27300000
H	25.51900000	24.75900000	57.47200000
C	23.81800000	24.79900000	56.22400000
N	24.19500000	23.80900000	55.39200000
H	25.17100000	23.58000000	55.51100000
H	23.68500000	23.56200000	54.55500000
N	22.64300000	25.34000000	55.94000000
C	22.33600000	26.42300000	56.71200000
O	21.51100000	23.14300000	60.89600000
P	22.77000000	23.85300000	60.12000000
O	23.81200000	24.02200000	61.18500000
O	22.22500000	25.02000000	59.44000000
O	23.42200000	22.72400000	59.17000000
C	24.16963184	21.61750487	59.68148330
H	23.67024151	20.70435080	59.43317112
H	25.14829004	21.61785210	59.24890036
H	24.24978057	21.70237517	60.74509658
C	20.27825750	22.79725586	60.25904874
H	19.78599256	22.03430829	60.82516378
H	19.65034902	23.66190797	60.20420653
H	20.47602959	22.43684776	59.27117618
C	19.98611901	26.76418621	55.97555426
H	20.25598175	26.86583167	54.94514546
H	19.68153359	25.75642921	56.16674412
H	19.17875858	27.42950143	56.20010889

1DUL-1314

C	14.74400000	35.62000000	52.89100000
H	14.77200000	35.41500000	51.82100000
N	16.03100000	35.15200000	53.42300000
C	16.58900000	35.40100000	54.67200000
H	16.18500000	36.08400000	55.40500000
N	17.55800000	34.62100000	54.97300000
C	17.83200000	33.88200000	53.81900000
C	18.77700000	32.82400000	53.54700000
O	19.60500000	32.24300000	54.20100000
N	18.57900000	32.25200000	52.31200000
H	19.11700000	31.46900000	51.97100000
C	17.57100000	32.59200000	51.44500000

N	17.62000000	32.01200000	50.29400000
H	18.31800000	31.29200000	50.17700000
H	16.98200000	32.34100000	49.58300000
N	16.78100000	33.59900000	51.62600000
C	16.92500000	34.18900000	52.82400000
C	12.64300000	30.81600000	55.01100000
H	12.97400000	31.71800000	54.49600000
O	13.74600000	30.32000000	55.76200000
P	15.24100000	30.69800000	55.38700000
O	16.14300000	29.94000000	56.30100000
O	15.30200000	32.19400000	55.52500000
O	15.47400000	30.26700000	53.84900000
C	15.95400000	28.97600000	53.56000000
H	15.47600000	28.34500000	54.30900000
H	17.02200000	28.90400000	53.76600000
H	15.67606422	28.57121148	52.60931711
H	12.18410532	30.13564178	54.32439359
H	11.89125199	30.95280459	55.76003901
H	13.91544687	35.14575328	53.37420778
H	14.59039692	36.66568252	53.05789566

1DUL-1923

N	18.76600000	23.94100000	50.41100000
C	19.14000000	23.82600000	51.75600000
H	18.46100000	23.85400000	52.59500000
N	20.42200000	23.56100000	51.86300000
C	20.93800000	23.40500000	50.59700000
C	22.24100000	22.97900000	50.09300000
O	23.26000000	22.75600000	50.76900000
N	22.27500000	23.01300000	48.73700000
H	23.06400000	22.56400000	48.29500000
C	21.30800000	23.46700000	47.85600000
N	21.53400000	23.34900000	46.58000000
H	22.36300000	22.87800000	46.24600000
H	20.90300000	23.78000000	45.92000000
N	20.05300000	23.79900000	48.29900000
C	19.94800000	23.74000000	49.70300000
O	18.66700000	18.16900000	51.44700000
P	19.66900000	19.42700000	51.32800000
O	20.83300000	19.17600000	52.18300000
O	18.90000000	20.62900000	51.63200000
O	20.05600000	19.44200000	49.81000000
C	17.44038723	24.07385434	49.78971060
H	17.43431019	24.92452304	49.14069148
H	17.22154068	23.19223656	49.22424064
H	16.70040940	24.20150687	50.55196885
C	17.47261780	18.09605320	50.66403773
H	17.59705288	17.36708642	49.89071804
H	16.65205937	17.81487484	51.29055395
H	17.27504295	19.05161586	50.22498853
C	21.14270430	18.70521837	49.24331967
H	21.05419354	18.70257187	48.17699006
H	22.06841573	19.16304281	49.52323954
H	21.11863167	17.69874249	49.60571007

1DUL-1658

C	29.47700000	33.88300000	69.69500000
H	29.25700000	33.20900000	68.86700000
N	30.87400000	34.46000000	69.71100000

C	31.40700000	35.63200000	70.13200000
H	30.78100000	36.44300000	70.47200000
N	32.75300000	35.67200000	70.08100000
C	33.09000000	34.39600000	69.55900000
C	34.38300000	33.90100000	69.16900000
O	35.52100000	34.31700000	69.40100000
N	34.18900000	32.78300000	68.36900000
H	35.03500000	32.34000000	68.03900000
C	32.98500000	32.22600000	68.01200000
N	32.87700000	31.04300000	67.38800000
H	33.74800000	30.59700000	67.13700000
H	31.95100000	30.64100000	67.33600000
N	31.82100000	32.69800000	68.36300000
C	31.94100000	33.79700000	69.12900000
C	31.17500000	30.82600000	73.76000000
H	30.60500000	31.21900000	72.91800000
O	32.57100000	31.06800000	73.70800000
P	33.15800000	32.35200000	73.02400000
O	34.20400000	32.83800000	73.95700000
O	32.06200000	33.30700000	72.66500000
O	33.87200000	31.81900000	71.68600000
C	34.85800000	30.79000000	71.81500000
H	35.11100000	30.69400000	72.87100000
H	35.76500000	31.07500000	71.28200000
H	31.02639817	29.77085593	73.85740885
H	30.75566439	31.10968846	74.70264442
H	34.63193416	29.82266498	71.41743593
H	29.23078672	33.38843245	70.61134161
H	28.79419796	34.69731016	69.57017874

1DUL-2489

C	16.22800000	27.47200000	38.83300000
H	16.72800000	28.24100000	38.24400000
N	17.27300000	26.45400000	39.04200000
C	17.41300000	25.58600000	40.06400000
H	16.76300000	25.62900000	40.92600000
N	18.31000000	24.69100000	39.89900000
C	18.86500000	24.91000000	38.60600000
C	19.73400000	24.18900000	37.77900000
O	20.50600000	23.25500000	38.02500000
N	19.67600000	24.61300000	36.44900000
H	20.15800000	24.05500000	35.75900000
C	19.06600000	25.69400000	36.06200000
N	19.12800000	25.94700000	34.76900000
H	19.77100000	25.35400000	34.26400000
H	18.74900000	26.80800000	34.40200000
N	18.23100000	26.48700000	36.79000000
C	18.17100000	26.03800000	38.08500000
C	13.91800000	22.45800000	36.30200000
H	14.52100000	23.35400000	36.44800000
O	14.65700000	21.31700000	35.88600000
P	16.15300000	21.19100000	36.44200000
O	16.49400000	19.73600000	36.47500000
O	16.43800000	22.00800000	37.64300000
O	17.07000000	21.81800000	35.26100000
C	17.42200000	20.98600000	34.15400000
H	16.73800000	20.14300000	34.05300000
H	18.44700000	20.61800000	34.18200000
H	13.43537764	22.30650419	37.24488105
H	13.14800036	22.72104336	35.60715560

```
H   17.41142977   21.55256437   33.24636952
H   15.40265647   27.02997501   38.31500197
H   15.88114902   27.86884751   39.76418551
```

Cartesian coordinates of additional loop interactions

1HQ1-694 "Stacking-1"

```
C   -1.84035127    3.07457219   -3.61253524
H   -1.66580487    3.42569236   -4.62954081
N   -0.82230586    3.74105949   -2.81794633
C   -1.05831350    4.55212694   -1.72443809
H   -2.01841948    4.64623909   -1.23955608
N    0.00749850    5.20673065   -1.24304425
C    1.04639588    4.73477408   -2.10483283
C    2.41043303    5.00285631   -2.18831241
N    3.01487213    5.87506489   -1.38320695
H    3.99652822    6.05487027   -1.54149931
H    2.37734918    6.32775898   -0.74386064
N    3.16099935    4.39852109   -3.13096579
C    2.56150457    3.42075763   -3.83310173
H    3.14950613    2.89005433   -4.56698479
N    1.26634714    3.01913648   -3.84158380
C    0.55788676    3.73893136   -2.96104449
C    2.22138143    0.38691311   -1.91647982
H    2.86306396    1.09982320   -2.43422097
N    1.68413764    1.04591348   -0.70513101
C    0.47848338    1.01437573   -0.03971876
H   -0.28832033    0.32009211   -0.34998234
N    0.22590963    1.94036343    0.82192764
C    1.50808810    2.52925271    0.92813994
C    2.06383896    3.51029682    1.76133963
N    1.44967709    4.24156037    2.63675443
H    2.05766498    4.91520342    3.08014018
H    0.44118799    4.23820308    2.67899911
N    3.34364680    3.93180215    1.59606896
C    4.03201755    3.40917167    0.61577189
H    5.05021693    3.74927813    0.49619852
N    3.61095910    2.41597826   -0.20592324
C    2.35409365    2.04337461   -0.00527923
H    2.78307708   -0.48555136   -1.65532135
H    1.43701582    0.04620918   -2.55959056
H   -1.62475365    2.03234866   -3.50213701
H   -2.80068816    3.33188303   -3.21700497
```

1HQ1-694 "Stacking-2"

```
C    2.22138143    0.38691311   -1.91647982
H    2.86306396    1.09982320   -2.43422097
N    1.68413764    1.04591348   -0.70513101
C    0.47848338    1.01437573   -0.03971876
H   -0.28832033    0.32009211   -0.34998234
N    0.22590963    1.94036343    0.82192764
C    1.50808810    2.52925271    0.92813994
C    2.06383896    3.51029682    1.76133963
N    1.44967709    4.24156037    2.63675443
H    2.05766498    4.91520342    3.08014018
H    0.44118799    4.23820308    2.67899911
N    3.34364680    3.93180215    1.59606896
C    4.03201755    3.40917167    0.61577189
H    5.05021693    3.74927813    0.49619852
N    3.61095910    2.41597826   -0.20592324
C    2.35409365    2.04337461   -0.00527923
```

C	7.23919742	-0.68536627	0.16877822
H	7.73877006	0.21703491	-0.18378450
N	6.12964174	-0.17641496	1.08311956
C	4.88380595	-0.78574564	1.37140869
H	4.52622311	-1.70771981	0.93734959
N	4.14397369	-0.15536106	2.28361194
C	4.99168987	0.86966246	2.68931299
C	4.94184566	1.91544912	3.66668299
N	3.83690482	2.14445724	4.39712346
H	3.89122936	2.92285686	5.03912191
H	2.99397632	1.60481101	4.25897989
N	5.89495258	2.76788321	3.75075739
C	7.01821083	2.58043297	3.13250612
H	7.75602362	3.35033046	3.30078355
N	7.27632719	1.60059893	2.28044365
C	6.20419710	0.80106197	2.02932018
H	7.90961296	-1.35911322	0.66021291
H	6.83761055	-1.16041403	-0.70182938
H	2.78307708	-0.48555136	-1.65532135
H	1.43701582	0.04620918	-2.55959056

1HQ1-694 "Hydrogen bond"

C	-2.54568723	0.31169443	3.83175753
H	-1.64688133	0.46818078	4.42864207
N	-2.58488329	-1.09919866	3.32474503
C	-3.72595535	-1.84700189	2.95423300
H	-4.71447738	-1.50962421	3.22745390
N	-3.52665862	-2.91164495	2.29784804
C	-2.20421946	-2.87090462	2.01904493
C	-1.37352313	-3.71148573	1.23205808
O	-1.69188130	-4.71536239	0.52373192
N	-0.07095763	-3.33462266	1.14348264
H	0.57959623	-3.88562876	0.60117728
C	0.41401466	-2.20610632	1.73138996
N	1.70524825	-1.98336036	1.61820742
H	2.21560032	-2.66843344	1.07914360
H	2.11712257	-1.42941515	2.35528163
N	-0.33975803	-1.44211727	2.54440394
C	-1.58475049	-1.82612823	2.65875249
C	7.23919742	-0.68536627	0.16877822
H	7.73877006	0.21703491	-0.18378450
N	6.12964174	-0.17641496	1.08311956
C	4.88380595	-0.78574564	1.37140869
H	4.52622311	-1.70771981	0.93734959
N	4.14397369	-0.15536106	2.28361194
C	4.99168987	0.86966246	2.68931299
C	4.94184566	1.91544912	3.66668299
N	3.83690482	2.14445724	4.39712346
H	3.89122936	2.92285686	5.03912191
H	2.99397632	1.60481101	4.25897989
N	5.89495258	2.76788321	3.75075739
C	7.01821083	2.58043297	3.13250612
H	7.75602362	3.35033046	3.30078355
N	7.27632719	1.60059893	2.28044365
C	6.20419710	0.80106197	2.02932018
H	7.90961296	-1.35911322	0.66021291
H	6.83761055	-1.16041403	-0.70182938
H	-2.53679946	0.92382895	2.95419708
H	-3.41070291	0.46567861	4.44244300

1HQ1-NP-784 "Stacking-1"

C	-1.85387676	-0.17101295	-2.33429758
H	-2.41562527	-0.68392339	-3.11369522
N	-1.41986363	-1.09731304	-1.24892112
C	-0.35592600	-0.94625602	-0.46460120
H	0.24956634	-0.06123087	-0.33545137
N	-0.23286880	-1.85140601	0.44441457
C	-1.34349884	-2.66452246	0.25208978
C	-1.95703800	-3.73985602	0.91551553
N	-1.40088272	-4.34680184	1.94298539
H	-1.90372378	-5.04684499	2.46963007
H	-0.45291818	-4.13877447	2.22212153
N	-3.08903202	-4.29027826	0.53578650
C	-3.73186190	-3.66889321	-0.43278494
H	-4.68343155	-4.06938601	-0.74967116
N	-3.37245286	-2.59416212	-1.12396905
C	-2.10762120	-2.17856134	-0.77943002
C	-7.00829167	-0.07953504	-0.12956325
H	-7.44903149	-0.96767423	-0.58127151
N	-5.85952994	-0.45932609	0.74559311
C	-4.65389924	0.17829817	0.89022758
H	-4.41682784	1.04954691	0.29693342
N	-3.81860614	-0.44287959	1.69507097
C	-4.60465445	-1.47281386	2.23296383
C	-4.43997391	-2.59846678	3.14918822
N	-3.45654467	-2.82770890	3.96152574
H	-3.41114163	-3.79813781	4.23824841
H	-2.61117884	-2.33168415	3.71667230
N	-5.47629787	-3.39126171	3.47444983
C	-6.62794393	-3.25078478	2.81786485
H	-7.44444188	-3.89951462	3.10010556
N	-6.89871964	-2.36389979	1.89165495
C	-5.83111021	-1.50894766	1.64777333
H	-6.72767547	0.69255186	-0.81515523
H	-7.75664792	0.33333997	0.51417361
H	-1.09911070	0.39051859	-2.84411177
H	-2.51010853	0.55038714	-1.89402293

1HQ1-NP-784 "Stacking-2"

C	2.48997322	-2.06374629	-3.82219856
H	2.13401270	-2.40150886	-4.77304479
N	2.14033877	-3.06489468	-2.77722346
C	2.90673993	-3.71238106	-1.85233108
H	3.93474830	-3.42527752	-1.68516793
N	2.30590320	-4.52902556	-1.07016344
C	1.01562820	-4.60130135	-1.57018434
C	-0.20954914	-5.32954823	-1.29184778
N	-0.37198246	-6.26440699	-0.40680211
H	-1.29405696	-6.54791265	-0.10730038
H	0.29713430	-6.46924600	0.32114835
N	-1.27826148	-5.18158119	-2.08415639
C	-1.16500060	-4.29073911	-3.04634223
H	-2.11836121	-4.14419714	-3.53270647
N	-0.16043870	-3.49283403	-3.41920698
C	0.93508158	-3.67645289	-2.62636608
C	-1.85387676	-0.17101295	-2.33429758
H	-2.41562527	-0.68392339	-3.11369522
N	-1.41986363	-1.09731304	-1.24892112
C	-0.35592600	-0.94625602	-0.46460120
H	0.24956634	-0.06123087	-0.33545137

N	-0.23286880	-1.85140601	0.44441457
C	-1.34349884	-2.66452246	0.25208978
C	-1.95703800	-3.73985602	0.91551553
N	-1.40088272	-4.34680184	1.94298539
H	-1.90372378	-5.04684499	2.46963007
H	-0.45291818	-4.13877447	2.22212153
N	-3.08903202	-4.29027826	0.53578650
C	-3.73186190	-3.66889321	-0.43278494
H	-4.68343155	-4.06938601	-0.74967116
N	-3.37245286	-2.59416212	-1.12396905
C	-2.10762120	-2.17856134	-0.77943002
H	-1.09911070	0.39051859	-2.84411177
H	-2.51010853	0.55038714	-1.89402293
H	3.55264368	-1.94485835	-3.86089178
H	2.03454602	-1.12534505	-3.58368405

1HQ1-NP-784 "Hydrogen bond"

C	2.49825610	-0.76952796	3.41664424
H	1.53458003	-1.02727254	3.80367935
N	2.56375578	0.73332000	3.17679404
C	3.64327511	1.54875016	3.34000233
H	4.53782465	1.15206458	3.79730729
N	3.47297742	2.74313292	2.83882695
C	2.14170688	2.77028002	2.43388737
C	1.30563767	3.79231998	1.89255856
O	1.62342598	4.92663041	1.48670063
N	-0.01443861	3.43104067	1.64505671
H	-0.60292549	4.05918270	1.11623545
C	-0.48146226	2.20082002	1.89182646
N	-1.72514769	1.95795547	1.60453285
H	-2.32995730	2.70223318	1.28804877
H	-2.07955819	1.01813106	1.71270130
N	0.26670435	1.21855677	2.41549324
C	1.56247297	1.50275369	2.63953357
C	-7.00829167	-0.07953504	-0.12956325
H	-7.44903149	-0.96767423	-0.58127151
N	-5.85952994	-0.45932609	0.74559311
C	-4.65389924	0.17829817	0.89022758
H	-4.41682784	1.04954691	0.29693342
N	-3.81860614	-0.44287959	1.69507097
C	-4.60465445	-1.47281386	2.23296383
C	-4.43997391	-2.59846678	3.14918822
N	-3.45654467	-2.82770890	3.96152574
H	-3.41114163	-3.79813781	4.23824841
H	-2.61117884	-2.33168415	3.71667230
N	-5.47629787	-3.39126171	3.47444983
C	-6.62794393	-3.25078478	2.81786485
H	-7.44444188	-3.89951462	3.10010556
N	-6.89871964	-2.36389979	1.89165495
C	-5.83111021	-1.50894766	1.64777333
H	-7.75664792	0.33333997	0.51417361
H	-6.72767547	0.69255186	-0.81515523
H	3.25263029	-1.05148512	4.12114387
H	2.66154880	-1.28547677	2.49358827

1JID-991 "Stacking-1"

C	-0.22526232	2.77545126	4.21902482
H	-0.58143824	2.74668537	5.24914492
N	-1.41066937	3.35499582	3.45684731
C	-1.37090878	4.40382249	2.51826166
H	-0.47841094	4.92905826	2.21337781

N	-2.50729199	4.84304540	2.14958068
C	-3.42050975	3.98712242	2.77840695
C	-4.88706187	3.94960040	2.83798270
O	-5.77073410	4.69123172	2.26456694
N	-5.32532037	2.85124979	3.61015673
H	-6.32082089	2.86405803	3.78065259
C	-4.53463510	2.02662803	4.31855775
N	-5.13176929	1.08128199	5.00827500
H	-6.12978054	1.16292454	5.13795989
H	-4.53893583	0.47497883	5.55670831
N	-3.23275644	2.00679601	4.33578805
C	-2.70368182	3.06262040	3.58323427
C	-3.56431491	-0.98856340	1.67662459
H	-4.54368225	-1.05465994	2.14988566
N	-3.50530729	0.21598323	0.84569510
C	-2.46565342	0.81957437	0.19953195
H	-1.48368111	0.37565949	0.13317582
N	-2.79382460	1.91406767	-0.41183236
C	-4.17473749	2.01932722	-0.14774657
C	-5.21625771	2.84997958	-0.61719472
N	-5.03006113	3.92978160	-1.38419647
H	-5.74599337	4.52164514	-1.78073116
H	-4.07634543	4.11271417	-1.66173237
N	-6.45495721	2.63121060	-0.25657453
C	-6.72334872	1.57556270	0.56607411
H	-7.73721999	1.42580907	0.90711955
N	-5.88065125	0.67623736	1.04336667
C	-4.57191396	0.91477807	0.57807180
H	-3.55884190	-1.90643083	1.12672511
H	-2.77703998	-0.96185689	2.40076887
H	-0.00528902	1.81722129	3.79676061
H	0.61905501	3.41702968	4.07617739

1JID-991 “Stacking-2”

C	-3.56431491	-0.98856340	1.67662459
H	-4.54368225	-1.05465994	2.14988566
N	-3.50530729	0.21598323	0.84569510
C	-2.46565342	0.81957437	0.19953195
H	-1.48368111	0.37565949	0.13317582
N	-2.79382460	1.91406767	-0.41183236
C	-4.17473749	2.01932722	-0.14774657
C	-5.21625771	2.84997958	-0.61719472
N	-5.03006113	3.92978160	-1.38419647
H	-5.74599337	4.52164514	-1.78073116
H	-4.07634543	4.11271417	-1.66173237
N	-6.45495721	2.63121060	-0.25657453
C	-6.72334872	1.57556270	0.56607411
H	-7.73721999	1.42580907	0.90711955
N	-5.88065125	0.67623736	1.04336667
C	-4.57191396	0.91477807	0.57807180
C	-7.05211723	-3.37953404	-1.43150609
H	-8.00202569	-3.01057043	-1.04366087
N	-6.34164391	-2.23396452	-2.02429650
C	-5.02371482	-1.96973443	-2.44195140
H	-4.16731623	-2.61585731	-2.31187897
N	-4.97222557	-0.82055927	-2.90208784
C	-6.22845882	-0.17479279	-2.81323985
C	-6.68482244	1.10287436	-3.33466501
O	-6.12421519	1.99903282	-4.01421543
N	-8.02346705	1.26347094	-3.01728566
H	-8.43747644	2.15584034	-3.24557980

C	-8.73759254	0.32021857	-2.36743879
N	-9.93071621	0.80413541	-1.90342830
H	-10.25693837	1.70171702	-2.23293626
H	-10.42819531	0.33132564	-1.16167860
N	-8.34554337	-0.85792823	-1.94988546
C	-7.06531073	-1.08259692	-2.25370719
H	-6.48214891	-3.87732217	-0.67503858
H	-7.22403423	-4.05468005	-2.24361595
H	-3.55884190	-1.90643083	1.12672511
H	-2.77703998	-0.96185689	2.40076887

1JID-991 "Hydrogen bond"

C	-0.01413648	3.52486785	-2.83678887
H	-1.03059864	3.13040548	-2.84937027
N	0.83608289	2.41721282	-3.32159365
C	2.16044339	2.36831073	-3.72688379
H	2.76208656	3.25329820	-3.87564489
N	2.71873007	1.17204575	-3.79227753
C	1.64956493	0.39108178	-3.39855246
C	1.58274889	-1.05532097	-3.26563136
O	2.38350387	-1.88843727	-3.48977394
N	0.31606527	-1.52546163	-2.97873482
H	0.22724380	-2.52563998	-2.87228226
C	-0.80641552	-0.77351909	-2.97141554
N	-1.90573546	-1.39573441	-2.59805107
H	-1.69458326	-2.28280494	-2.16409972
H	-2.71292864	-0.78992100	-2.54442815
N	-0.81192745	0.55051704	-3.09792715
C	0.47635784	1.06801216	-3.29292742
C	-7.05211723	-3.37953404	-1.43150609
H	-8.00202569	-3.01057043	-1.04366087
N	-6.34164391	-2.23396452	-2.02429650
C	-5.02371482	-1.96973443	-2.44195140
H	-4.16731623	-2.61585731	-2.31187897
N	-4.97222557	-0.82055927	-2.90208784
C	-6.22845882	-0.17479279	-2.81323985
C	-6.68482244	1.10287436	-3.33466501
O	-6.12421519	1.99903282	-4.01421543
N	-8.02346705	1.26347094	-3.01728566
H	-8.43747644	2.15584034	-3.24557980
C	-8.73759254	0.32021857	-2.36743879
N	-9.93071621	0.80413541	-1.90342830
H	-10.25693837	1.70171702	-2.23293626
H	-10.42819531	0.33132564	-1.16167860
N	-8.34554337	-0.85792823	-1.94988546
C	-7.06531073	-1.08259692	-2.25370719
H	-6.48214891	-3.87732217	-0.67503858
H	-7.22403423	-4.05468005	-2.24361595
H	0.23843323	3.74417129	-1.82041564
H	0.02414816	4.40930172	-3.43779721

1JID-NP-1145 "Stacking-1"

C	2.00472739	-2.81036165	-3.69032776
H	1.60863629	-2.99345333	-4.68856945
N	1.44925444	-3.81251768	-2.71495735
C	2.08608238	-4.38902119	-1.72420001
H	3.10926394	-4.08513563	-1.55377929
N	1.29173945	-5.03708166	-0.89874790
C	0.03737774	-5.13724470	-1.61272996

C	-1.19672385	-5.87396049	-1.43328532
O	-1.52475965	-6.67599275	-0.53094113
N	-2.23841090	-5.44771864	-2.29249964
H	-3.13790591	-5.90346604	-2.24307125
C	-1.95311605	-4.64815805	-3.26924881
N	-2.78334453	-4.58240652	-4.31905435
H	-3.71474600	-4.97100916	-4.28978921
H	-2.47145118	-3.97166663	-5.06017612
N	-0.83831332	-4.02853968	-3.55651729
C	0.16979095	-4.27542229	-2.66831233
C	-1.97244030	0.02026863	-2.13113625
H	-2.64413767	-0.63508632	-2.68467730
N	-1.54821342	-0.89123019	-0.94625533
C	-0.42829252	-0.79634669	-0.11944242
H	0.35918378	-0.10333276	-0.37710488
N	-0.37353322	-1.60333816	0.84808868
C	-1.65095627	-2.26304042	0.74678935
C	-2.23864810	-3.27074665	1.46343761
N	-1.74485105	-3.83745657	2.56202470
H	-2.30498306	-4.54896976	3.00929560
H	-0.92785651	-3.45447169	3.01607058
N	-3.35546970	-3.87465242	1.14447586
C	-3.94643947	-3.41207608	0.00811758
H	-4.89732390	-3.90361118	-0.13341051
N	-3.50498512	-2.49540485	-0.85109727
C	-2.31710354	-1.88209693	-0.35711243
H	-2.56531093	0.86844316	-1.85909876
H	-1.21166214	0.36941309	-2.79763062
H	1.64385298	-1.84433668	-3.40490629
H	3.07348672	-2.79321680	-3.64175259

1JID-NP-1145 “Stacking-2”

C	-1.97244030	0.02026863	-2.13113625
H	-2.64413767	-0.63508632	-2.68467730
N	-1.54821342	-0.89123019	-0.94625533
C	-0.42829252	-0.79634669	-0.11944242
H	0.35918378	-0.10333276	-0.37710488
N	-0.37353322	-1.60333816	0.84808868
C	-1.65095627	-2.26304042	0.74678935
C	-2.23864810	-3.27074665	1.46343761
N	-1.74485105	-3.83745657	2.56202470
H	-2.30498306	-4.54896976	3.00929560
H	-0.92785651	-3.45447169	3.01607058
N	-3.35546970	-3.87465242	1.14447586
C	-3.94643947	-3.41207608	0.00811758
H	-4.89732390	-3.90361118	-0.13341051
N	-3.50498512	-2.49540485	-0.85109727
C	-2.31710354	-1.88209693	-0.35711243
C	-6.54246530	0.90198899	0.17599308
H	-6.94179340	-0.03448907	-0.21360042
N	-5.52711179	0.48429157	1.16697817
C	-4.60193619	1.18699078	1.73576124
H	-4.49448158	2.22073508	1.44077075
N	-3.89365862	0.56508641	2.67131247
C	-4.59683952	-0.59561996	2.75967314
C	-4.42266155	-1.73109670	3.61500518
O	-3.69553407	-1.78354699	4.63424501
N	-5.19146607	-2.82074514	3.32480499
H	-4.80045859	-3.70024832	3.63280572
C	-6.12258156	-2.84150407	2.34831216
N	-6.85598678	-3.88320081	2.17422624

H	-6.70382917	-4.68873574	2.76389961
H	-7.28153657	-3.99209749	1.26484565
N	-6.38735725	-1.75468219	1.60812682
C	-5.60354208	-0.69906909	1.83294906
H	-7.36538091	1.43184297	0.60838718
H	-6.11377775	1.46832694	-0.49002493
H	-2.56531093	0.86844316	-1.85909876
H	-1.21166214	0.36941309	-2.79763062

1JID-NP-1145 "Hydrogen Bond"

C	2.38375716	-1.05787641	3.27024645
H	1.32301572	-1.13937963	3.50686427
N	2.74536767	0.40650918	3.33115610
C	3.98860853	0.90652884	3.33761847
H	4.90743600	0.36384762	3.50203967
N	4.01675957	2.15557907	2.95275968
C	2.66038754	2.50238153	2.70716398
C	2.02523769	3.61944277	2.07622408
O	2.63544314	4.58502112	1.61504086
N	0.63649507	3.51453930	1.90849960
H	0.24484902	4.18518244	1.26223551
C	-0.08419013	2.41324113	2.30615832
N	-1.28897012	2.31733446	1.84297009
H	-1.69082927	3.11598091	1.37329550
H	-1.90946252	1.56434310	2.10554072
N	0.50736283	1.29742833	2.80219175
C	1.85890249	1.41839372	2.98104976
C	-6.54246530	0.90198899	0.17599308
H	-6.94179340	-0.03448907	-0.21360042
N	-5.52711179	0.48429157	1.16697817
C	-4.60193619	1.18699078	1.73576124
H	-4.49448158	2.22073508	1.44077075
N	-3.89365862	0.56508641	2.67131247
C	-4.59683952	-0.59561996	2.75967314
C	-4.42266155	-1.73109670	3.61500518
O	-3.69553407	-1.78354699	4.63424501
N	-5.19146607	-2.82074514	3.32480499
H	-4.80045859	-3.70024832	3.63280572
C	-6.12258156	-2.84150407	2.34831216
N	-6.85598678	-3.88320081	2.17422624
H	-6.70382917	-4.68873574	2.76389961
H	-7.28153657	-3.99209749	1.26484565
N	-6.38735725	-1.75468219	1.60812682
C	-5.60354208	-0.69906909	1.83294906
H	-7.36538091	1.43184297	0.60838718
H	-6.11377775	1.46832694	-0.49002493
H	2.67292612	-1.47541922	2.32847189
H	3.01342919	-1.54282886	3.98665014
